# Peptide backbone modifications of amyloid β (1–40) impact fibrillation behavior and neuronal toxicity

**DOI:** 10.1038/s41598-021-03091-4

**Published:** 2021-12-09

**Authors:** Benedikt Schwarze, Alexander Korn, Corinna Höfling, Ulrike Zeitschel, Martin Krueger, Steffen Roßner, Daniel Huster

**Affiliations:** 1 Institute for Medical Physics and Biophysics, Leipzig University, Härtelstr. 16/18, 04107 Leipzig, Germany; 2 Paul Flechsig Institute for Brain Research, Leipzig University, Liebigstr. 19, 04103 Leipzig, Germany; 3 Institute of Anatomy, Leipzig University, Liebigstr. 13, 04103 Leipzig, Germany

**Keywords:** Alzheimer's disease, Intrinsically disordered proteins

## Abstract

Fibril formation of amyloid β (Aβ) peptides is one of the key molecular events connected to Alzheimer’s disease. The pathway of formation and mechanism of action of Aβ aggregates in biological systems is still object of very active research. To this end, systematic modifications of the Phe_19_–Leu_34_ hydrophobic contact, which has been reported in almost all structural studies of Aβ_40_ fibrils, helps understanding Aβ folding pathways and the underlying free energy landscape of the amyloid formation process. In our approach, a series of Aβ_40_ peptide variants with two types of backbone modifications, namely incorporation of (*i*) a methylene or an ethylene spacer group and (*ii*) a *N*-methylation at the amide functional group, of the amino acids at positions 19 or 34 was applied. These mutations are expected to challenge the inter-β-strand side chain contacts as well as intermolecular backbone β-sheet hydrogen bridges. Using a multitude of biophysical methods, it is shown that these backbone modifications lead, in most of the cases, to alterations in the fibril formation kinetics, a higher local structural heterogeneity, and a somewhat modified fibril morphology without generally impairing the fibril formation capacity of the peptides. The toxicological profile found for the variants depend on the type and extent of the modification.

## Introduction

Alzheimer’s disease (AD), being one of over 40 maladies caused by protein or peptide aggregation and misfolding, is a progressive and multifactorial disease leading to age-related changes in the brain. The etiology of AD is complex and influenced by genetic, environmental, and/or lifestyle factors^1^. In spite of intense research efforts invested into different aspects of this devastating form of dementia, AD is currently still incurable^2^. Progress in developing therapeutic treatment options is, among others, hampered by the long progression of the disease without any clinical symptoms, while moderate to severe levels of amyloid aggregates and neurofibrillary tangles in the brain result in the loss of neurons, disturbance of neuronal connectivity and in brain atrophy. The key molecular events underlying AD are fibril formation of amyloid β (Aβ) peptides and posttranslational modifications of the tau protein^3,4^.

However, amyloid fibril formation does not follow only a single pathway, but occurs through several routes in the energy landscape^5,6^. Intermediates that are formed on the way include soluble oligomers, to which strong neurotoxicity is ascribed via different molecular mechanisms such as membrane disruption, receptor-binding followed by activation of signaling pathways or glutamatergic neuronal hyperactivation^7–9^.

Details of Aβ fibril formation have been intensively investigated in the last two decades predominantly using solid state NMR and cryo-EM approaches in combination with molecular modeling, Structural models of the transient Aβ oligomers/protofibrils^10–12^ and structures of Aβ fibrils^13–17^ have been determined extensively.

**Figure 1:**
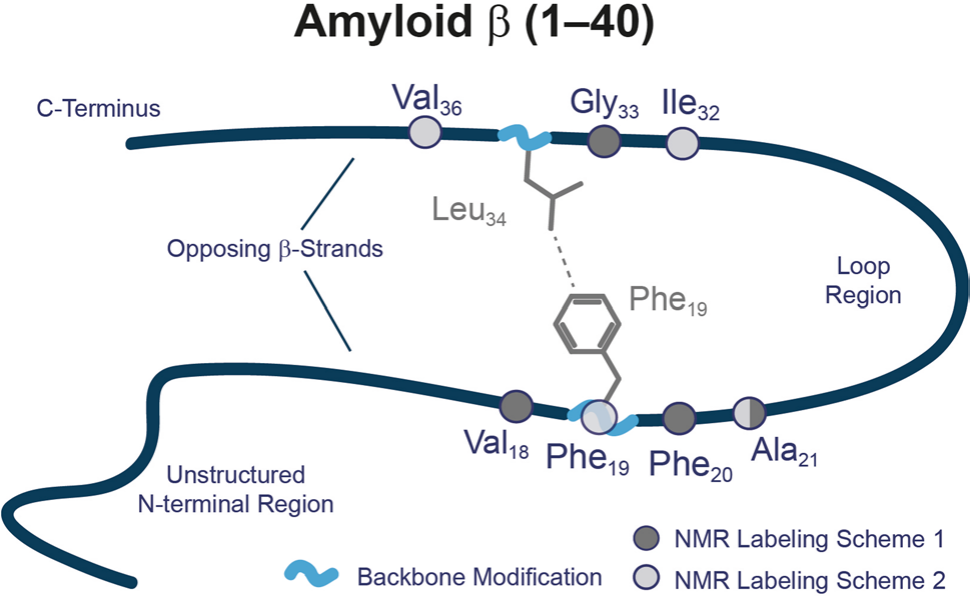
Schematic depiction of the Aβ_40_ structure visualizing the backbone modifications (light blue waves) introduced and the NMR labeling schemes applied. From the N-terminus, the peptide fibrils feature an unstructured N-terminus continue with β-strand 1 which ends at the beginning of the loop region, followed by β-strand 2 and the C-terminus. Both β-strands oppose each other with the side chains of the respective amino acids interacting in a steric-zipper motif. We targeted the hydrophobic contact between Phe_19_ and Leu_34_ by introduction of backbone modifications, namely methylene or ethylene spacer or *N*-methylation of the amide, in both residues in different combinations (see Fig. 2). Two isotopic labeling schemes (light and dark gray spheres) were applied for the investigations of local structure and dynamics of Aβ_40_ fibrils by solid-state NMR.

What remains highly enigmatic in the field of protein misfolding in general is the observation that peptides and proteins of no or of very diverse three-dimensional structure can form amyloids, in which the cross-β structure is the only common conformational motif^18,19^. This suggests that very general physical interactions cause these peptides or proteins to aggregate and form fibrils. To investigate the contributions of specific physical interactions systematically, targeted perturbations induced by individual mutations in the Aβ sequence represent a useful strategy^20–23^. Each amino acid carries specific properties such that exchange of a given residue introduces a new physical force and the response of the system to such a perturbation can be analyzed in detail. Such knowledge can be useful for the development of small molecular weight drugs that feature exactly that property for preventing formation of toxic Aβ structures. In our previous work, a rational library of relatively minor modifications was introduced to modify one of the most crucial molecular contacts in Aβ_40_ fibrils between the two opposing β-strands, which provided important insights. In a series of studies, we systematically modified the Phe_19_–Leu_34_ hydrophobic contact (Fig. 1), which has been found in almost all structural studies of Aβ_40_ fibrils^13–16^. Overall, these studies showed that the Aβ_40_ structure is very robust against almost any of the introduced modifications in the side chains of either Phe_19_ or Leu_34_. For example, a double mutation of the hydrophobic contact between Phe_19_ and Leu_34_ into two Lys residues (Phe_19_Lys and Leu_34_Lys) introduced two positive charges, electrostatically repelling each other, which forced these side chains to move out of the hydrophobic core of the β-cross structure^24^. However, the general morphology of the fibrils was only marginally influenced. An introduction of a proline into position Phe_19_ prolonged the fibrillation times (based on ThT fluorescence assay) dramatically^25^, but left the final fibril morphology largely unchanged. The only remarkable exception to the high tolerance of Aβ_40_ against any modification of the Phe_19_/Leu_34_ contact was that fibrillation was completely abrogated when a salt-bridge (Lys_19_–Glu_34_) was introduced to replace the hydrophobic contact between Phe_19_ and Leu_34_^25,26^. Taken together, while local alterations in structure and dynamics of Aβ_40_ have been observed, these studies showed that the Aβ_40_ cross-β structure is very robust, extremely stable and can tolerate and accommodate many local alterations in the physical interaction fields. However, any specific mutation influenced the toxicity of the Aβ_40_ fibrils suggesting that the Phe_19_ and Leu_34_ is of crucial importance as an early folding contact in Aβ_40_ structure formation^27^.

**Figure 2:**
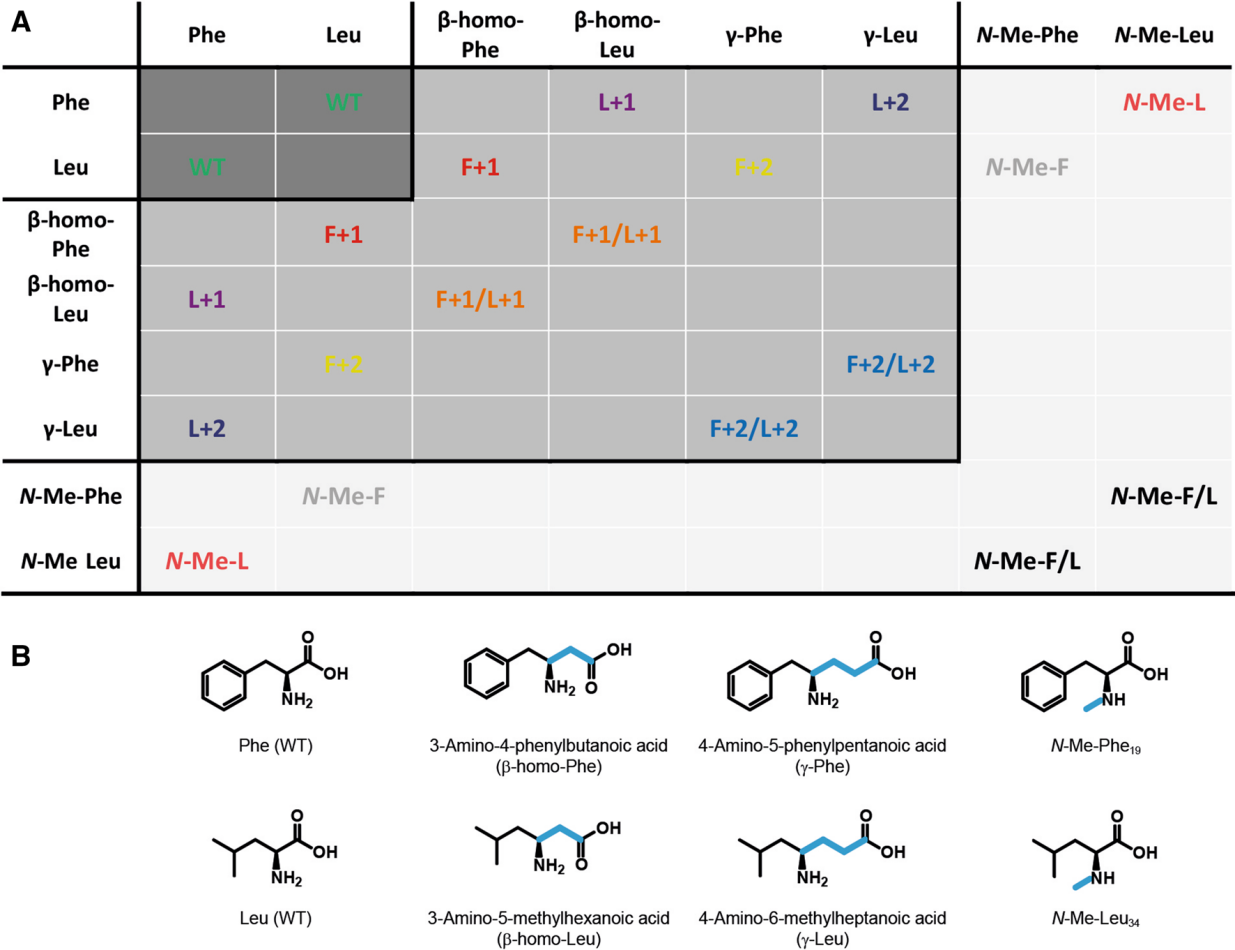
(**A**) Summary of the backbone mutation scheme applied to Aβ_40_ peptides used in this study. The color code is used throughout the manuscript in the respective data. (**B**) Chemical structure of the backbone modified amino acids introduced into positions 19 and 34, respectively.

In the current study, we turn our attention to the backbone of Aβ_40_ peptides and investigate the impact of two modifications of the Aβ_40_ peptide backbone at and around the important positions Phe_19_ and Leu_34_. These modifications correspond to (*i*) an extended backbone structure (by the introduction of a methylene (**X+1**) or ethylene (**X+2**) spacer) and (*ii*) a peptide backbone *N*-methylation***-Me-X***) (*N*-Me-X) pattern (see Fig. 2). The rationale was that an extended backbone should induce a register shift to the standard in-register parallel β-sheet organization of the well-known steric zipper motif^13,28^ of Aβ_40_ fibrils. Such a shift in register would challenge the inter-β-strand side chain contacts as well as backbone β-sheet hydrogen bridges. The *N*-methylation of the peptide backbone would disturb the intermolecular hydrogen bonds between neighboring β-sheets^29–31^. To achieve that, we used combinations of Phe, Leu, 3-amino-4-phenylbutanoic acid (β-homo-Phe), 3-amino-5-methylhexanoic acid (β-homo-Leu), 4-amino-5-phenylpentanoic acid (γ-Phe), 4-amino-5-methylheptanoic acid (γ-Leu), *N*-methyl-phenylalanine (*N*-Me-Phe) and *N*-methyl-leucine (*N*-Me-Leu) as summarized in Figs. 1 and 2. With these modifications, the amyloid peptides receive more degrees of freedom by either (*i*) a more flexible backbone or (*ii*) a steric constraint, and the mutations are expected to introduce more structural variability into the system. This would (*i*) influence the register between the two opposing β-strands in the Aβ_40_ structure^15,32–34^ and (*ii*) interfere with the hydrogen bonding networks within the parallel intermolecular β-sheet arrangement^29–31^.

We used a broad repertoire of analytical techniques for the investigation of the amyloid fibrillation kinetics (thioflavin T (ThT) and crystal violet (CV) fluorescence spectroscopy), fibril morphology (transmission electron microscopy (TEM)), mesoscopic fibril packing parameters (X-ray diffraction), and fibril local structure and dynamics (solid-state nuclear magnetic resonance (NMR) spectroscopy). Further analyses include cellular toxicity studies (lactate dehydrogenase (LDH) release assay, 3-(4,5-dimethylthiazol-2-yl)-2,5-diphenyltetrazolium bromide (MTT) conversion assay, activated caspase-3 and neurite length measurements) to determine the biological impact of the modifications and, thus, the relevance for pathologies observed in AD.

## Results

### Fibrillation kinetics using the ThT fluorescence assays

**Figure 3:**
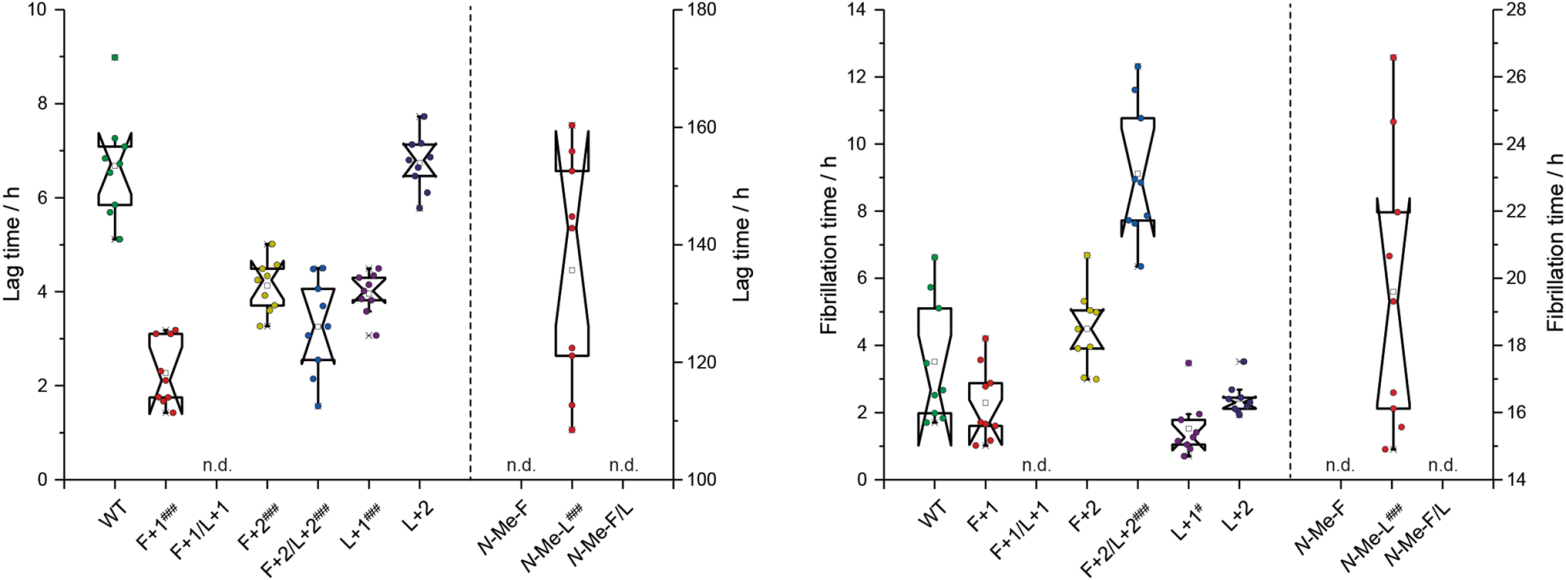
Box plot representation of the two characteristic parameters lag time (left) and fibrillation time (right) determined from ThT fluorescence fibrillation kinetics experiments. The peptide variants ***N*-Me-F** and ***N*-Me-F/L** do not fibrillate, while data for **F+1/L+1** could not be fitted with the applied function (n.d. means not determined). Data from three independent experiments with three replicates each are shown. Peptide concentration was 0.125 mg mL^−1^ in an aqueous 25 mM sodium phosphate buffered solution (pH 7.4) containing 150 mM NaCl and 0.01%(w/v) NaN_3_. All normalized fluorescence intensity vs. time plots are given in the supporting information (supplementary Fig. S1). Significance was tested applying a heteroscedastic Student's t-Test using two-tailed distributions, ^#^p ≤ 0.05; ^##^p ≤ 0.01; ^###^p ≤ 0.001 vs **WT**.

The fibrillation kinetics of the investigated mutant and wild type Aβ_40_ peptides were followed by recording the thioflavin T (ThT) fluorescence intensity over time which indicates formation of amyloid β fibril structures^35^. The characteristic parameters that can be determined from a plot of the fluorescence intensity vs. time (see supplementary Fig. S1) are the lag time (*t*_lag_) and fibrillation time (*t*_fib_), which are presented in Fig. 3 for all peptide variants.

All Aβ_40_ variants with extended backbone modifications, except **F+1/L+1**, show the typical sigmoidal fluorescence intensity curves as a function of time (see Fig. S1, SI) consisting of the three characteristic phases: the lag phase, the fibrillation phase, and the plateau phase. Based on the results, **F+2/L+2** (*t*_lag_ = 3.3 ± 1.0 h; *t*_fib_ = 9.1 ± 1.9 h) shows a lag time shorter than the **WT** (*t*_lag_ = 6.7 ± 1.1 h; *t*_fib_ = 3.5 ± 1.7 h), but a significantly longer fibrillation time until approaching the plateau phase, indicating that the increased degrees of freedom in the peptide backbone alter the energy landscape of the fibrillation process. The lag times for **F+1** (*t*_lag_ = 2.3 ± 0.7 h; *t*_fib_ = 2.3 ± 1.1 h), **F+2** (*t*_lag_ = 4.1 ± 0.5 h; *t*_fib_ = 4.5 ± 1.1 h) and **L+1** (*t*_lag_ = 4.0 ± 0.4 h; *t*_fib_ = 1.5 ± 0.8 h) are shorter than for the **WT**, and **L+2** (*t*_lag_ = 6.7 ± 0.6 h; *t*_fib_ = 2.4 ± 0.5 h) is in the same order of magnitude, showing that most of the mutations provide a stimulus for an earlier start of the fibrillation process. On the other hand, the fibrillation times are very similar.

Interestingly, the double mutations seem to have a rather strong impact on the fibrillation kinetics, as the **F+1/L+1** variant shows two fibrillation phases, the first one starting immediately after dissolution in the fibrillation buffer reaching the plateau phase relatively fast and a second slower one starting after about 20 h (see supplementary Fig. S1). This behavior is reminiscent of the studies by Muschol and co-workers who concluded that sigmoidal kinetics indicate basically “oligomer-free” fibril formation, whereas in a biphasic growth the initial phase is dominated by globular oligomers^36,37^. For that purpose, they screened for an oligomer-specific dye and identified crystal violet (CV) as such. Therefore, we conducted fibril formation assays with CV for **WT**, **F+1/L+1** and **F+2/L+2** and found that for **F+1/L+1** the CV fluorescence responses are indicative of fast oligomer formation with a constant concentration level up to 20 h which is not the case for the other two investigated peptides (Fig. S2, SI).

Noticeably, the backbone methyl modified ***N*-Me-F** and ***N*-Me-F/L** variants do not fibrillate at all under the applied conditions and ***N*-Me-L** has a very extensive lag time of 135.7 ± 18.5 h and a somewhat longer fibrillation time of 19.4 ± 4.0 h. This reveals that methylation of the amide group in the peptide backbone has a tremendous effect on the formation of fibrils, where disturbances at Phe_19_ seem to be more severe. This effect was also observed earlier by Meredith and co-workers^30,31^ and was related to inhibition of amyloid β toxicity of some short peptides^38,39^, from which a whole strategy of aggregation inhibitors in medicinal chemistry was developed^40,41^.

### Aβ_40_ fibril morphology investigated by transmission electron microscopy

**Figure 4:**
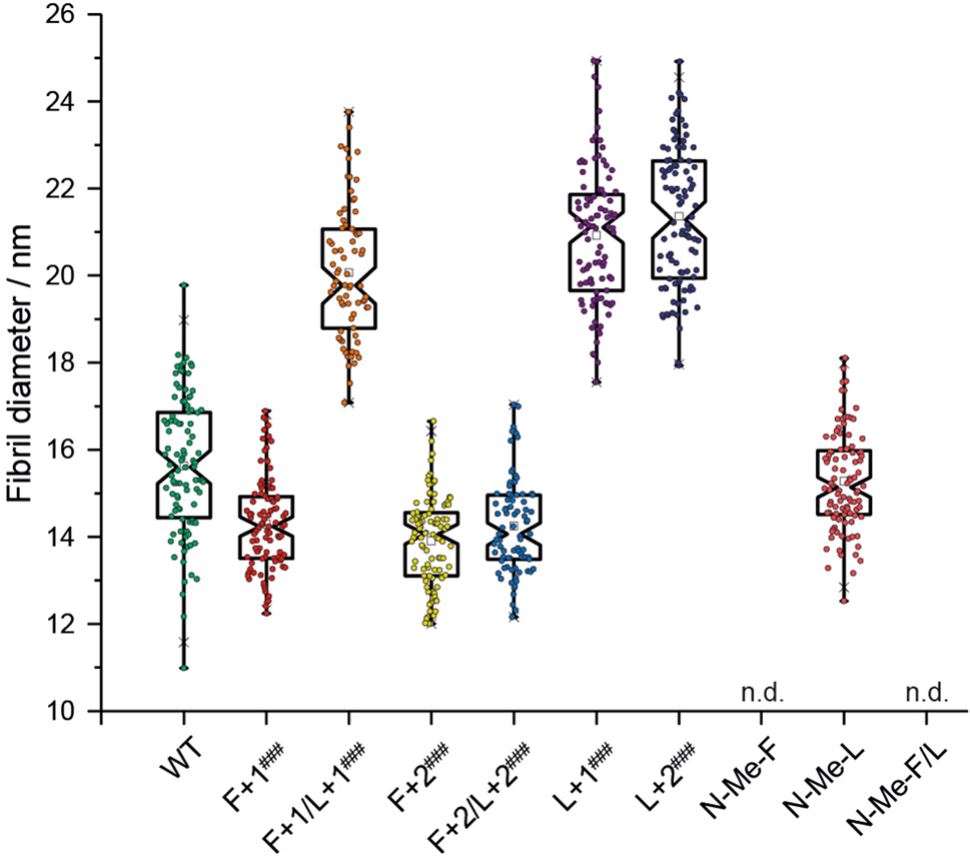
Box plot showing the diameters of fibrils formed from all Aβ_40_ peptide variants determined based on TEM images. ***N*-Me-F** and ***N*-Me-F/L** do not fibrillate (n.d. means not determined); minor second fibril population of **F+1/L+1** and **F+2/L+2** not shown. Significance was tested applying a heteroscedastic Student's t-Test using two-tailed distributions, ^#^p ≤ 0.05; ^##^p ≤ 0.01; ^###^p ≤ 0.001 vs **WT**.

To study the morphology of the Aβ_40_ fibrils, transmission electron microscopy (TEM) experiments were performed on fibrils formed during the ThT fluorescence fibrillation assay (see supplementary Fig. S3 for TEM images). All mutants with extended backbones form fibrils, while only the ***N*-Me-L** mutant among the *N*-methylated ones fibrillated (Fig. 3). The fibril diameter, determined from the TEM images, of **F+1** (14.3 ± 1.1 nm), **F+2** (13.9 ± 1.0 nm), **F+2/L+2** (14.3 ± 1.1 nm) are in the same order of magnitude and slightly smaller than ***N*-Me-L** (15.3 ± 1.1 nm) and the **WT** (15.6 ± 1.6 nm), while **F+1/L+1** (20.1 ± 1.5 nm), **L+1** (20.9 ± 1.6 nm) and **L+2** (21.4 ± 1.6 nm) form fibrils with a larger diameter (about + 5 nm) (Fig. 4). Only the double mutants **F+1/L+1** and **F+2/L+2** also show additionally a minor population with larger diameter (about + 5 nm, respectively; data not shown).

Furthermore, topological differences among the mutants were analyzed showing that the **WT**, **F+1**, **F+2**, **F+2/L+2** and the larger population of **F+1/L+1** form fine and distinct fibrils, where the other mutants form fibrils reminding of crystalline needles grown together along the long side. Notably, some mutants are shorter in length (**F+1**, minor population of **F+1/L+1**, **F+2**, **L+1** and ***N*-Me-L**), than others (major population of **F+1/L+1**, **F+2/L+2** and **L+2**).

### Mesoscopic cross-β structure investigated by X-ray diffraction

**Figure 5:**
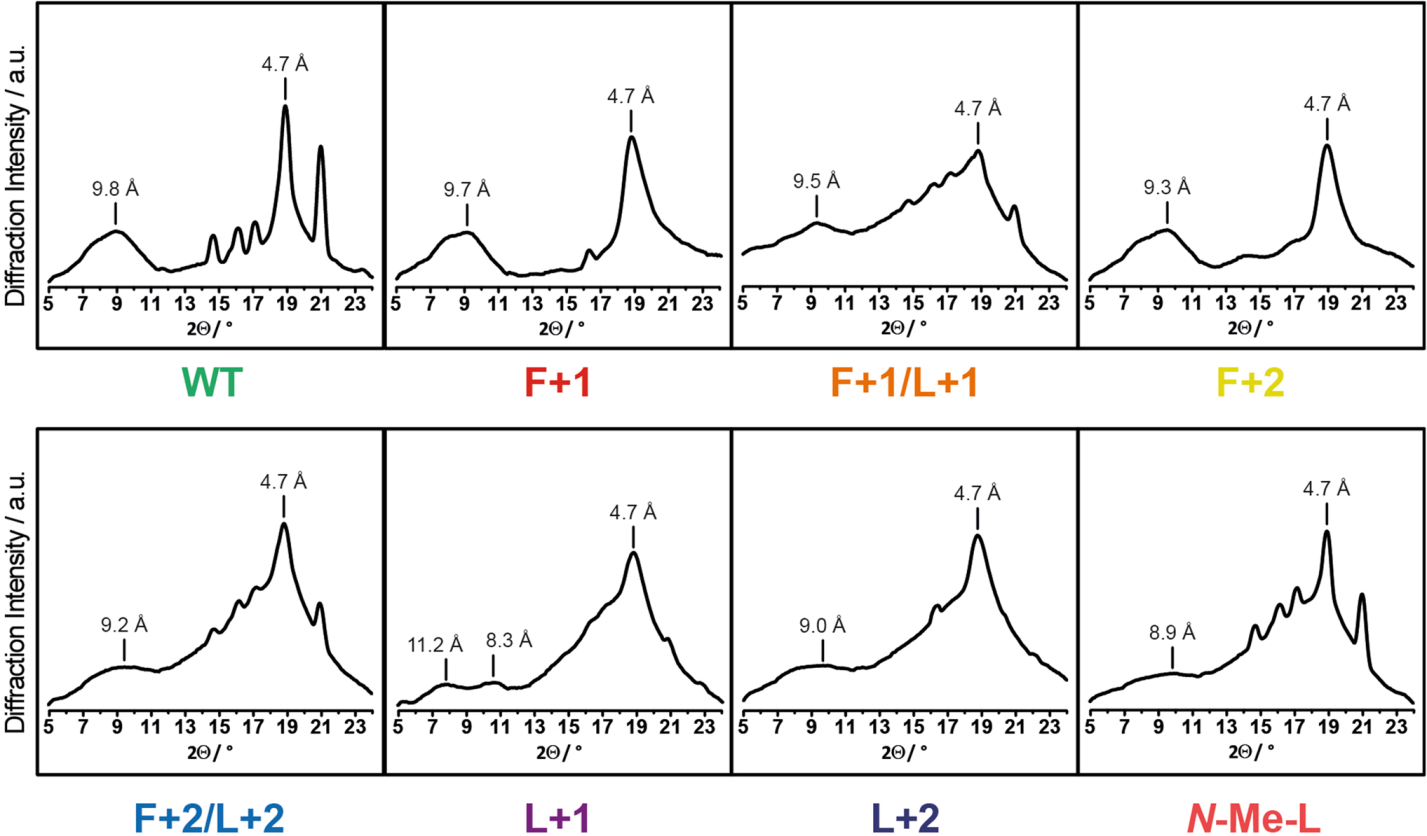
X-ray diffraction patterns of fibrils formed from the Aβ_40_ variants. Characteristic inter-sheet (8.3–11.2 Å) and inter-strand (4.7 Å) distances are assigned. Other reflections in the range of 2θ = 14–18° and at 21° result from salts present in the amyloid samples and are not of significance.

For X-ray powder diffraction measurements, samples of the respective Aβ_40_ fibrils were mounted to nylon loops using oil and measured using Cu K_α_ X-ray radiation (Cu K_α_, λ = 1.5406 Å). By applying Braggs law, the detected Debye cones, represented in the diffraction intensity vs 2θ plot (Fig. 5), could be transformed into the inter-sheet and inter-strand distances^42^. Relatively sharp reflections in the range of 2θ = 14–18° and at 21° result from salts present in the amyloid samples.

All the mutants show an intense and relatively well-defined reflection assigned to inter-strand spacing (4.7 Å) which is characteristic for the cross-β structure of amyloid fibrils (along growing direction)^42,43^. For **WT**, **F+1** and **F+2** this peak is relatively sharp, while for all the mutants, having a mutation at the Leu_34_ site, the peak is particularly broadened indicating more structural heterogeneity. Additionally, the signals for the inter-sheet distances are even more diffuse and less intense compared to **WT**, **F+1** and **F+2**. Both observations are a hint towards higher structural heterogeneity in the packing when the Leu_34_ is mutated. Within the series **WT** (9.8 Å), **F+1** (9.7 Å), and **F+2** (9.3 Å) the packing becomes slightly tighter while the overall diffraction pattern remains similar. Interestingly, **L+1** shows two separated reflections (11.2 and 8.3 Å), which is unique for this mutant with this particular clarity. In summary, especially Leu_34_ site mutations introduce a type of perturbation into the fibril structure, which leads to shorter inter-sheet distances on an average, but with rather diffuse distributions.

### Investigation of the local molecular structure of the mutated Aβ_40_ fibrils by solid-state NMR spectroscopy

**Figure 6:**
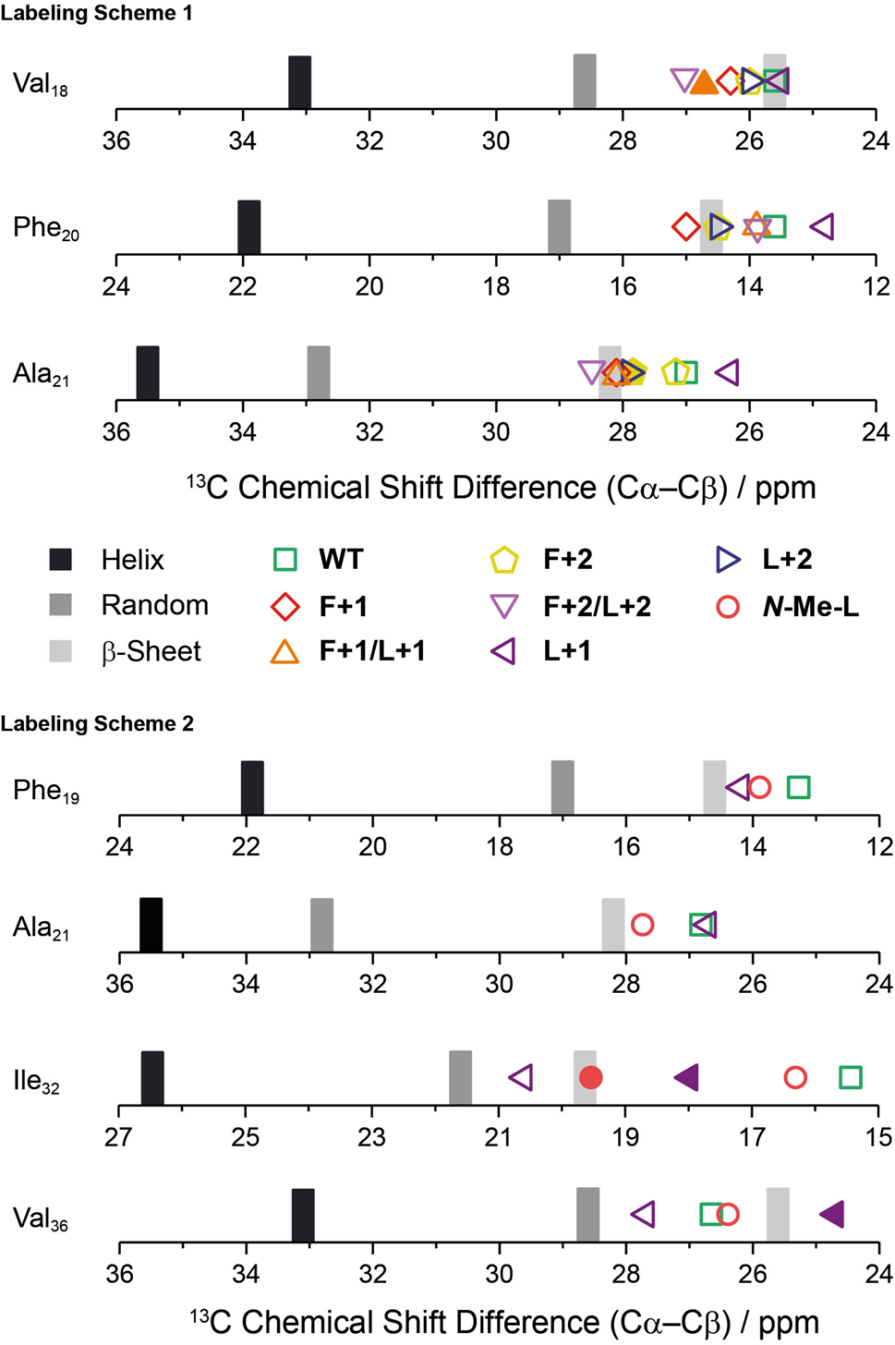
Overview of the ^13^Cα –^13^Cβ chemical shift differences of the labeled amino acids subdivided into the two applied labeling schemes: Val_18_, Phe_20_, Ala_21_, Gly_33_ (labeling scheme 1) and Phe_19_, Ala_21_, Ile_32_, Val_36_ (labeling scheme 2) derived from ^13^C–^13^C DARR NMR experiments. Gly_33_ is not shown because it is lacking a Cβ atom. The bars represent the reference values of α-helix (black), random coil (gray) and β-sheet (light gray) secondary structure as reported in the literature^44^. Experimental data is given as varying symbols in the usual color code according to the legend. The standard error for determination of chemical shifts from ^13^C–^13^C DARR NMR experiments is ± 0.5 ppm. Other conformations, if applicable, are shown as filled forms in the respective color and shape (polymorphs of Val_18_ in **F+1/L+1** (labeling scheme 1) are identical).

To obtain insights into the secondary structure on the level of individual amino acids, solid-state NMR spectra of the peptide variants that formed fibrils were recorded using two different isotopic (^13^C/^15^N) labeling schemes (see Fig. 1). One of them was chosen for investigating the local influence of the mutations in the vicinity of the Phe_19_ mutation site (labeling scheme 1: Val_18_, Phe_20_, Ala_21_, Gly_33_). In the other case, the labeling scheme involving Phe_19_, Ala_21_, Ile_32_, Val_36_ (labeling scheme 2) concentrates on the mutations on the Leu_34_ site. It was designed to identify potential inter-β-strand contacts caused by peptide backbone register shifts (**L+1**) or peptide backbone *N*-methylation (***N*-Me-L**). The assignments of the signals of the aforementioned uniformly ^13^C/^15^N labeled amino acids were done by means of either co-acquisition of ^13^C–^13^C DARR and ^13^Cα–^15^N correlation NMR experiments^22^ or ^13^C–^13^C DARR experiments. A summary of all ^13^C and ^15^N chemical shifts of the labeled nuclei are listed in supplementary Tables S1 and S2. Calibration-independent differences of the ^13^Cα and ^13^Cβ signals are reported for both labeling schemes, which are characteristic for the local secondary structure of the respective amino acids (Fig. 6)^44^. Some of the mutants with labeling scheme 1, namely **L+1** (Gly_33_), **F+2** (Ala_21_), **F+1/L+1** (Ala_21_, Gly_33_) and **F+2/L+2** (Gly_33_), as well as with labeling scheme 2, **L+1** (Ile_32_, Val_36_) and ***N*-Me-L** (Ile_32_), show polymorphisms while **WT**, **L+2** and **F+1** do not (supplementary Tables S1 and S2). This highlights again the higher degree of freedom in some modified Aβ peptides allowing diverse modification and possibly multiple interactions^45,46^. However, no conclusive pattern among the investigated mutants could be found in this regard.

In almost all the mutants, except for **L+1**, the signal for the carbonyl group (CO) could not be detected in the ^13^C NMR spectra for Gly_33_ (supplementary Table S1), so that it cannot be used as a parameter for the local impact of the mutation on Gly_33_. The ^15^N NMR signals for Ala_21_ in the double mutants, **F+1/L+1** and **F+2/L+2**, are relatively strongly upfield shifted (119.4 and 118.7 ppm, respectively), which indicates effects on hydrogen-bonding between β-strands (supplementary Table S1). In labeling scheme 1, all investigated mutants show β-sheet secondary structures, suggesting that the induced perturbation by extended backbones does not influence the local secondary structure around the Phe_19_ mutation site compared to the **WT**. However, **L+1** seems to be special in the sense that its ^13^Cα–^13^Cβ chemical shift differences, at least for Phe_20_ and Ala_21_, is significantly smaller than for **WT**. Therefore, **L+1** was chosen to be investigated in labeling scheme 2 together with ***N*-Me-L**. Their ^13^Cα–^13^Cβ chemical shift difference values for amino acids Phe_19_ (**L+1**: 13.9 ppm; ***N*-Me-L**: 14.1 ppm) and Ala_21_ (**L+1**: 26.6 ppm; ***N*-Me-L**: 27.4 ppm) are similar as for **WT** (13.2 and 26.7 ppm, respectively) but differ relevantly for Ile_32_ and Val_36_ (Fig. 6). Interestingly, the two conformations of Ile_32_ (**L+1**: 18.0 and 20.7 ppm; ***N*-Me-L**: 16.3 and 19.6 ppm) or Val_36_ (**L+1**: 24.8 and 27.7 ppm) differ from each other and from the **WT**. For **L+1** both conformers even vary between predominantly random coil and β-sheet structure. Polymorphism or structural variation on the molecular level occurs frequently in amyloid fibril formation and may even correlate with diverging progress of the diseases among patients^47–49^. In conclusion, the local secondary structure around the Leu_34_ mutation site is more sensitive towards backbone mutations of these types than Phe_19_.

In addition to amyloid structure determination, NMR spectroscopy is a very sensitive tool to obtain information on molecular dynamics. The order parameter (*S*) derived from the measurement of motional averaged ^1^H–^13^C dipolar couplings using DipShift experiments^50,51^ yields information on the motional amplitude of a given C–H bond vector. The molecular order parameters of the labeled residues in the Aβ_40_ variants are shown in supplementary Fig. S4. Overall, no drastic changes in the order parameters throughout the variants using labeling scheme 1 were observed. For amino acid Gly_33_ the Cα–Hα bond vector became a bit more rigid compared to the **WT**, when any type of mutation was introduced, but interestingly the conformers for Gly_33_ for **F+1/L+1** or **L+1** differ in mobility. Worth noticing, in **F+1/L+1** the phenyl ring of Phe_20_ is slightly more rigid compared to **WT** and the other mutants. For labeling scheme 2, the observations are relatively similar, no drastic changes were observed. The only detail worth highlighting is that for **L+1** all carbons show a tendency for higher rigidity, except for the phenyl ring in Phe_19_, where the amplitude for the ring motions increased a bit, which might give a hint towards the influence of a mutation at Leu_34_ onto its crucial contact partner, the Phe_19_.

**Figure 7:**
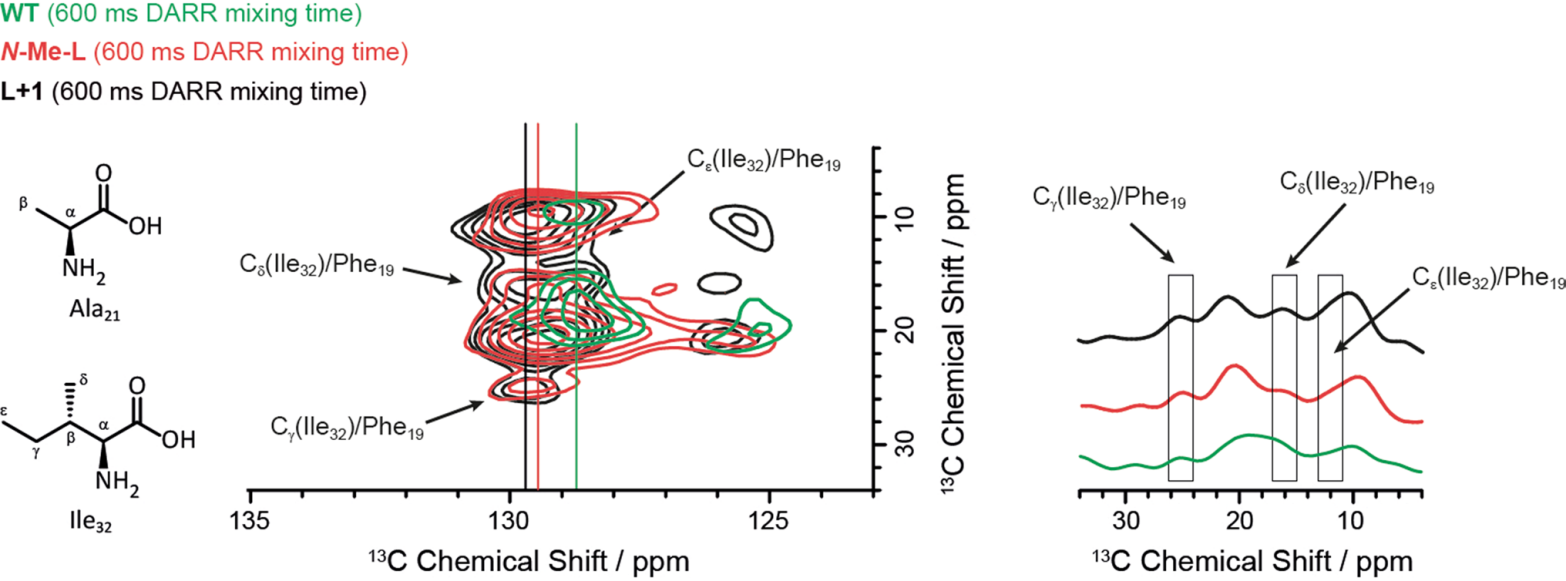
Sections from the contour plot of ^13^C–^13^C DARR NMR spectra (30 °C, MAS frequency of 11,777 Hz) of **L+1** (129.6 ppm), ***N*-Me-L** (129.4 ppm) and **WT** (128.7 ppm) using labeling scheme 2 showing a structural change upon mutation at Leu_34_. Left: schematic molecular structure of Ala_21_ and Ile_32_ including labeling; center: contour plot of ^13^C-^13^C DARR NMR spectra for ** +1** (black), ***N*-Me-L** (red) and **WT** (green); right: projection of ^13^C-^13^C DARR NMR spectra for **L+1** (black), ***N*-Me-L** (red) and **WT** (green) along y-axis.

In order to examine a potential register-shift between the Phe_19_ and Leu_34_ contact in the opposing β-strands (Fig. 1) induced by either backbone extension or backbone *N*-methylation, we exemplarily chose **L+1** and ***N*-Me-L** for further NMR analysis. To this end, the amino acids Phe_19_, Ala_21_, Ile_32_, Val_36_ (labeling scheme 2) were ^13^C and ^15^N isotopically labeled, which only allows for mutations at Leu_34_. ^13^C–^13^C DARR NMR experiments were performed with mixing times of 600 ms to probe the long distance and 50 ms for short distance contacts. For **L+1** the cross peaks at around 129.6/12–25 ppm, which are assigned to side chain contacts between the labeled side chains of Ile_32_ and Phe_19_, are only detected in the DARR NMR spectrum recorded with long 600 ms mixing time (Fig. 7, center). The **WT**, on the other hand, also shows this contact (Fig. 7, right), although the chemical shift of the Phe ring system is slightly lower. However, comparing the recorded NMR spectra for **L+1** and ***N*-Me-L**, the same contact between Ile_32_ and Phe_19_ was detected also for ***N*-Me-L** (Fig. 7, center). This leads to the conclusion that no significant alterations in the sidechain zipper motif or a register shift are induced by the backbone modifications. But we note that the ring of Phe_19_ is also in contact with the side chain of Ile_32_ which was not reported before.

### Cellular toxicity assays of mutated Aβ fibrils

**Figure 8:**
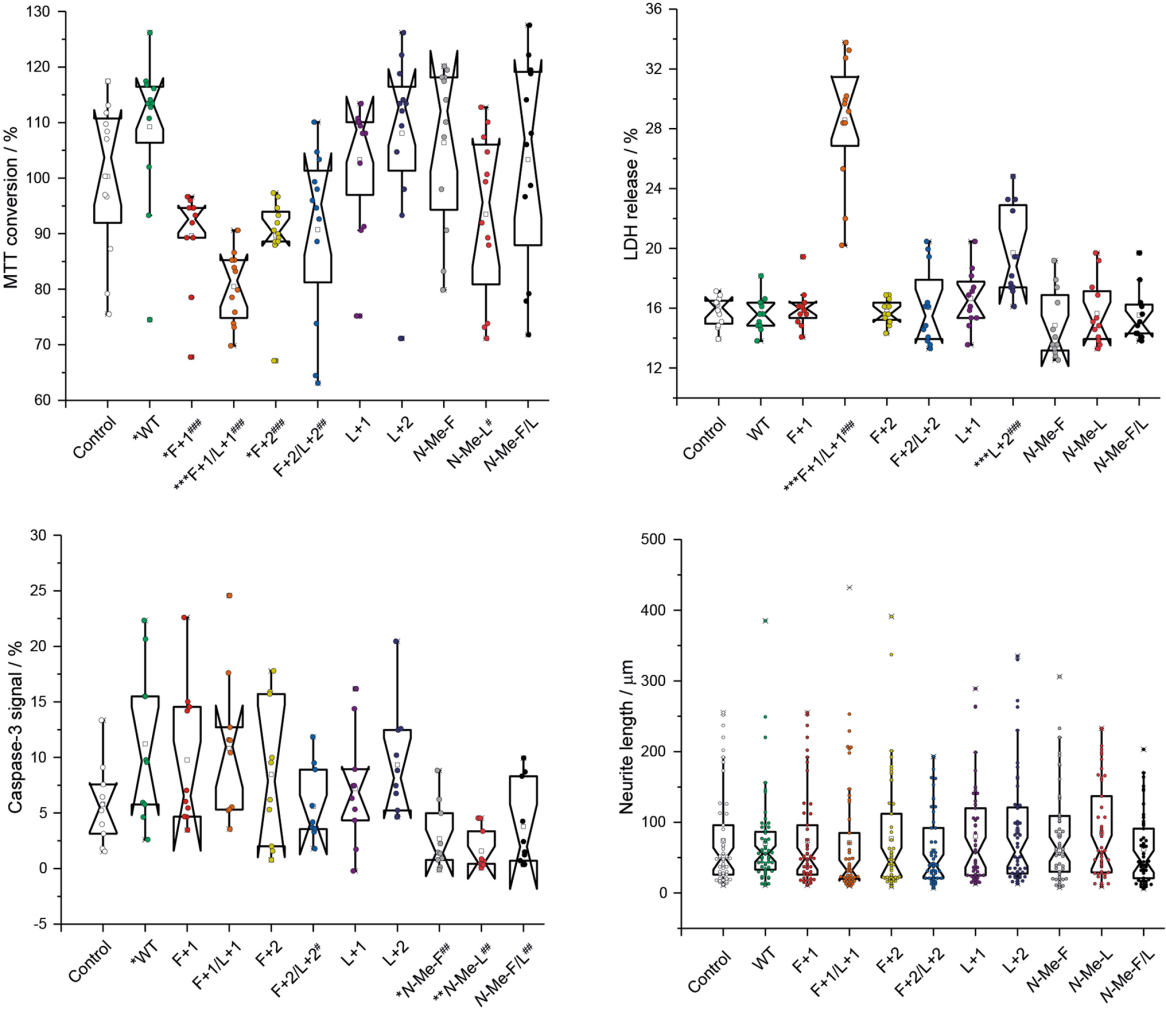
Box plots showing results from toxicity studies of all Aβ_40_ peptide variants. The MTT conversion assay (upper left) represents the proportion of metabolically active cells (alive) upon exposure to Aβ_40_ peptide variants; the LDH release (upper right) is indicative for plasma membrane disruption (dead cells) under the same conditions; caspase-3 staining (bottom left) is used to investigate one of the possible pathways towards cell apoptosis; neurite length (bottom right) measurements are used to examine the effect of all Aβ_40_ peptide variants on the outgrowth of neurites and thus the wellbeing under the applied conditions. Mutant **F+1/L+1** stands out in MTT and LDH, while in the caspase-3 staining the *N*-methylated variants show protective effects. The neurite outgrowth behavior is not significantly impacted by any of the mutants. Significance was tested applying a heteroscedastic Student's t-Test using two-tailed distributions, *p ≤ 0.05; **p ≤ 0.01; ***p ≤ 0.001 vs control; ^#^ vs **WT**.

To probe the biological activity of the individual Aβ_40_ variants, four different cell culture-based experiments were conducted (Fig. 8). In a first assay, the MTT (3-(4,5-dimethylthiazol-2-yl)-2,5-diphenyltetrazolium bromide) conversion for mitochondrial activity was performed which provides information about the proportion of metabolically active cells (upper left). Noticeably, **F+1/L+1** has the clearest negative impact on the cell viability compared to control and **WT** among all the mutants. Same is true for the second type of cell viability testing, being the lactate dehydrogenase (LDH) assay. It is based on the release of the cytosolic enzyme LDH into the cell culture medium upon damage to the plasma membrane (indicating cell death). For that, **F+1/L+1** exhibit a considerably destructive effect on the cell membrane integrity, while only **L+2** is also significantly different to the control and **WT**, but not as pronounced compared to **F+1/L+1**.

The outcome of the immunocytochemical staining for activated caspase-3, which is a protein involved in apoptotic neuronal cell death program, is interesting due to the apparently protective activity of the *N*-methylated peptides, namely ***N*-Me-F** (supplementary Table S1), ***N*-Me-L** (supplementary Table S1) and ***N*-Me-F/L** (supplementary Table S1), by suppressing the caspase-3 activation compared to control.

Neurite length (in µm) as a marker of neuronal viability was measured after incubation with the mutant peptides. However, no significant differences were detected (Fig. 8) indicating no impact of the individual Aβ_40_ variants on this aspect of neuronal wellbeing of the surviving neurons.

## Discussion

The formation of amyloid fibrils, which is the process of conversion of soluble peptide monomers into insoluble aggregates with fibrillar structure, follows the widely accepted nucleation-dependent polymerization model^18,19,52,53^. Recent research has shown that the individual aggregation pathways of proteins and peptides can be very complex and there are several detours in addition to the sigmoidal kinetic fibrillation behavior, indicating that local structural minima exist on the free energy map^36,54^. This is an intrinsic property of the funnel-shaped free energy landscape of amyloid formation^55,56^. Nevertheless, various folding pathways lead to a highly stable low energy structure that is dominated by the cross-β motif. In our approach, we probe how the folding of Aβ_40_ peptides is influenced by point mutations at Phe_19_ and Leu_34_, which form an important early hydrophobic contact in the Aβ_40_ folding process. These rational modifications can provide information on key points on the energy landscape of the fibril formation process. Our former activities involved, among others, alterations of the Phe_19_ and/or Leu_34_ side chains^25,57–59^. In the current study, we focus our attention to the peptide backbone, which we modified by (*i*) extension in the backbone structure by introduction of methylene (**X+1**) or ethylene (**X+2**) spacers and (*ii*) *N*-methylation of the backbone amide groups (***N*-Me-X** (supplementary Table S1))^60^ (Fig. 2). In these peptide variants, the backbone extension increases the degrees of freedom in the peptide backbone and could perturb the ordered in-register hydrophobic zipper structure motif. The *N*-methylation of the amide abolishes the formation of the intermolecular hydrogen bond between neighboring β-strands at the respective mutation sites.

Our previous work on fibril formation of side chain modified Aβ_40_ peptides led us to conclude that the amyloid structure is extremely robust against a multitude of perturbations introduced by individual natural and non-natural side chain modifications^25,57–59^. This conclusion also holds for the Aβ_40_ peptides with backbone extensions as applied here. While moderate alterations in the fibrillation kinetics (Fig. 3), fibril morphology (Fig. 4) and some heterogeneity of the cross-β structure (Fig. 5) are observed, the general β-sheet backbone secondary structure and the cross-β conformation are essentially not influenced by these mutations. The only exception is the **L+1** variant, where Ile_32_ shows some local random coil structure. Overall, the steric-zipper motif of the side chains^33,60^ that describes the interlocking of the amino acid residues along with the intermolecular hydrogen bond patterns remain the dominating structure forming principle also in fibrils of backbone extended Aβ_40_ peptides. Thereby, the side chains remain in a tightly packed conformation as confirmed by high order parameters determined in DipShift NMR experiments indicating low conformational flexibility. Although backbone extensions provide additional degrees of freedom, the interlocking of the side chains is the dominating motif that remains largely unchanged. There are some alterations worth mentioning in this context, though: first, there is an increasing amount of structural polymorphism observed in the ^13^C NMR spectra of some of the Aβ_40_ mutants, which demonstrates increased structural heterogeneity of the fibrils of mutated Aβ_40_ peptides^46,49^. This is confirmed by noticeably highfield-shifted ^15^N NMR signals of Ala_21_ in the double-mutants **F+1/L+1** and **F+2/L+2** (119.4 ppm, 118.7 ppm, respectively, vs. 126.1 ppm in **WT**; NMR labeling scheme 1). Also, variant **L+1** showed some structural alterations towards random coil structure of Ile_32_. For this variant, also a somewhat altered side chain packing was observed. Inversely, the lag times for the backbone extension variants in the ThT fibrillation kinetics are overall shorter as compared to the **WT**, while the fibrillation times are in the same order of magnitude. The backbone extension modification might thus even lower the activation energy barrier for early nucleation. In the fluorescence fibrillation plots, the double mutants, **F+1/L +1** and **F+2/L+2**, show fibrillation kinetics diverging from normal sigmoidal curves under the applied conditions. For **F+2/L+2**, it is reminiscent of kinetics based on catalyzed nucleation (e.g. seeding)^61^, where the “seed” is formed during the lag time made from **F+2/L+2** itself, which is apparently attributed to this specific combination of backbone modifications. ThT fibrillation curves of **F+1/L+1** look like two step fibrillation kinetics resembling curves from the literature on oligomer and fibril formations^37^. Kinetic fluorescence assays with an oligomer-selective dye (crystal violet, CV) revealed the presence of oligomers in the first 20 h of fibrillation^37^ (Fig. S2, SI). A hint towards understanding this abnormal behavior has been noticed in the difference of the ^15^N chemical shift for Ala_21_ for both the mutants with respect to the other variants and the **WT**. The potential existence of oligomeric species could be associated with the very effective membrane disruption ability of this mutant (Fig. 8), as oligomeric amyloid species are discussed as being the villains in cell toxicity^62–66^. Indirectly, an attenuating effect of **F+1/L+1** on the MTT conversion by mitochondria is probably also related to the membrane disruption ability rather than mechanisms activating mitochondrial apoptotic pathways such as caspase-3 activation.

In contrast to the moderate influence of the backbone extensions on Aβ_40_ fibril formation and structure, the *N*-methylation modification of the peptide backbone causes a more severe interference with Aβ_40_ structure formation. *N*-methylation is one of the strategies to stabilize peptides against proteolysis and makes them more water soluble^31,40^. Likewise, methylation that interferes with intermolecular hydrogen bond formation is used for the design of amyloid-β aggregation inhibitors^67,68^. There are at least three identified ways how *N*-methylation impairs protein aggregation: (*i*) replacement of the amide proton cuts the hydrogen-bonding possibility between two neighboring β-strands; (*ii*) prevention of regular packing through steric hindrance (higher steric demand) and (*iii*) tertiary amides favoring *trans*-conformation leading to β-strand conformation, which, in this case, does not disturb the secondary structure^69^. Thus, the current results fit into the picture that fibrillation kinetics are either completely blocked (***N*-Me-F**, ***N*-Me-F/L**) or greatly slowed down (***N*-Me-L**). In the literature, examples by Meredith and co-workers, who used two times two *N*-methylated amino acids (2NMe(NTerm): residues 17 and 19; 2NMe(CTerm): residues 37 and 39), fibrils could still be formed, although significantly slower than in absence of the modification^29^. Only four *N*-methylated amino acids in the same peptide could prevent the fibrils from their formation. Noticeably, they could show that the β-sheet secondary structure for the respective strands is disrupted independently from each other according to their modification pattern, while *N*-methylation at position 17 and 19 has a higher impact on the fibril formation process, as it is true also for ***N*-Me-F** and ***N*-Me-F/L** compared to ***N*-Me-L**. However, the striking difference is that in our case *N*-methylation at only one (Phe_19_) or two residues (Phe_19_/Leu_34_), which were identified to be a critical early folding contact in fibril extension^27^, are sufficient to prevent from fibril formation. The protective behavior of ***N*-Me-F**, ***N*-Me-L** and ***N*-Me-F/L** counteracting cytotoxicity is expressed in the assay measuring caspase-3 activity, where they significantly decrease caspase-3 activation compared to the **WT** and partly to the control. This is in alignment with other *N*-methylated peptides in the literature^70^.

With the experimental techniques applied, no conclusive statement about the underlying Aβ_40_ structural model, be it the U-shape (β arcades)^32^, C-shape (e.g. ex vivo Aβ_40_ fibril, PDB: 6SHS)^71^, or the recently found extended shape model^72^, can be made. The Aβ_40_ Glu_22_Δ (Iowa mutant)^73^ structure (PDB: 2MVX) and the threefold symmetric Aβ_40_ structure (PDB: 2M4J) by Lu et al.^74^, however, can be excluded, since Ala_21_ and Phe_20_ show Cα–Cβ chemical shift differences characteristic for β-sheet structure. Nevertheless, for **L+1** and ***N*-Me-L** contacts between Phe_19_ and Ile_36_ (see NMR labeling scheme 2 and Fig. 7) could be found, which points towards a U-shape model, which we assume also for the other variants. In this context, it seems likely that the smallest fibril unit is not significantly impaired by the register-shift mutations at Phe_19_ or Leu_34_ and the *N*-methylation at Leu_34_.

Regardless of the individual backbone modifications, parallel hydrogen-bonded β-strands are formed along the fibrillation axis (4.8 Å) verified by X-ray diffraction measurements^33.^ Interestingly, when in general a mutation is introduced at Leu_34_ the inter-β-sheet distance (around the distinct reflection for 4.8 Å) is more diffuse compared to **WT**, **F+1** and **F+2**. This implies that the 3D space, required by the side chain of Leu_34_, introduces more structural inhomogeneity during packing along the fibrillation axis in the mutants, than the planar phenyl ring does. This is also true for the *N*-methylated variant meaning that the extended backbone modification and the *N*-methylation are in terms of local packing density/arrangement more similar than initially expected. This hypothesis is supported by the findings from ^13^C–^13^C DARR NMR experiments (NMR labeling scheme 2) on **L+1**, ***N*-Me-L** and **WT**, where the inter-β-strand contact between Phe_19_ and Ile_32_ could be found for both the mutants due to the same steric demand of the modification (see Fig. 7). All these observations lead to the interpretation that backbone extensions have more impact on Leu_34_, while *N*-methylations do on Phe_19_.

## Conclusion

In summary, in this study, a series of two types of Aβ_40_ peptide backbone modification, namely incorporation of (*i*) a methylene or ethylene spacer group and (*ii*) a *N*-methylation at the amide functional group, at the amino acids at positions 19 or 34 were investigated. This approach was designed to probe the response of the Aβ_40_ system to the introduced backbone perturbations to identify key points in the free energy landscape of fibril formation. In all peptide variants that formed fibrils, both types of modification at either position 19 and 34 do not disturb the cross-β structure and the hydrophobic zipper interdigitation of the side chains and their dynamic behavior in the β-sheet parts of the putative U-shaped fibrils. However, the modifications lead, in most of the cases, to alterations in fibril formation kinetics, higher local structural heterogeneity, and somewhat modified fibril morphology without generally impairing the fibril formation capacities of the peptides. Both backbone extensions provide additional degrees of freedom, allowing for a more efficient structure formation process as seen in faster fibrillation kinetics. In contrast, *N*-methylation significantly delays or even completely abolishes fibrillation of the Aβ_40_ variants. This concludes a series of systematic studies where the influence of local perturbations in the Aβ_40_ sequence were introduced. While most modifications only lead to moderate alterations in the fibril formation process and structure, inter-sheet hydrogen bond formation turns out to be the most important contribution to Aβ_40_ fibril formation. Even the lack of only one or two inter-strand hydrogen bonds can completely abolish fibril formation stressing its importance for Aβ40 fibril formation. However, essentially all modifications introduced in the Aβ_40_ sequence led to alterations in one way or another in biological activity profiles as concluded from toxicity assays. This hints towards a more pronounced impact of these modifications on the transient oligomeric states of the Aβ_40_ peptide variants.

## Methods

### Peptide synthesis

Standard Fmoc solid phase synthesis was used to produce the Aβ_40_ peptides with the WT sequence DAEFRHDSGY EVHHQKLV**F**F AEDVGSNKGA IIG**L**MVGGVV and six variants with the extended backbone modifications, namely β-homo-Phe_19_ (**F + 1**), β-homo-Leu_34_ (**L+1**), β-homo-Phe_19_/β-homo-Leu_34_ (**F+1/F+1**), γ-Phe_19_ (**F+2**), γ-Leu_34_ (**L+2**), γ-Phe_19_/γ-Leu_34_ (**F+2/L+2**) and three mutants with *N*-methylated amide groups, namely *N*-Me-Phe_19_ (***N*-Me-F**), *N*-Me-Leu_34_ (***N*-Me-L**), *N*-Me-Phe_19_/*N*-Me-Leu_34_ (***N*-Me-F**/***N*-Me-L**) (the mutated residues are highlighted in bold in the peptide sequence). For NMR measurements, uniform ^13^C/^15^N labeled amino acids were introduced at positions Val_18_, Phe_20_, Ala_21_, and Gly_33_ (labeling scheme 1) or Phe_19_, Ala_21_, Ile_32_, Val_36_ (labeling scheme 2) (underlined in the peptide sequence). Peptide synthesis was performed by the Peptide Synthesis Core Unit of Leipzig University (https://home.uni-leipzig.de/izkf/indexpeptide.html). The peptide purity level determined by HPLC analysis and MALDI mass spectrometry was ≥ 97% depending on the peptide (see supporting information).

### Aβ_40_ fibril preparation

Peptide powder was dissolved in dimethyl sulfoxide (DMSO) (ca. 8.3–8.6 mg peptide in 80 μL DMSO), incubated at room temperature for ca. 30 min and then diluted with aqueous buffer (25 mM sodium phosphate, 150 mM NaCl, 0.01%(w/v) NaN_3_, pH 7.4) at a concentration of 1 mg mL^−1^. For fibrillation, the sample was transferred into 1.5 mL reaction tubes and incubated for 14 days at 37 °C and 450 rpm in a thermoshaker. Fibrils prepared by this procedure were used for X-ray diffraction and NMR measurements. For transmission electron microscopy measurements, fibrils were taken out of the 96-well plate from thioflavin T (ThT) fluorescence fibrillation assay after completion of the fibrillation process.

### Data representation

Experimental data derived from thioflavin T (ThT) fluorescence, transmission electron microscopy (TEM) and toxicological assays and stainings are summarized in box plot representations. The box marks the interquartile range (IQR) delimiting 50% of the data; the empty square is the mean, while the notch represents the 95% confidence interval (CI) of the median; the whiskers show 1.5 times IQR (not the standard deviation) demarcating statistical outliers and crosses indicate 1% or 99% of the data, respectively. Significances were tested applying a heteroscedastic Student's t-Test using two-tailed distributions, *p $\le$ 0.05; **p $\le$ 0.01; ***p $\le$ 0.001 vs control; # vs **WT**.

### Thioflavin T (ThT) fluorescence measurements

Peptide powder was dissolved in dimethyl sulfoxide (DMSO) (ca. 0.5–0.8 mg peptide in 10 μL DMSO), incubated at room temperature for ca. 30 min and diluted at a concentration of 0.125 mg mL^−1^ (in two steps: first 1 mg mL^−1^, then 0.125 mg mL^−1^) with aqueous buffer (25 mM sodium phosphate, 150 mM NaCl, 0.01%(w/v) NaN_3_, pH 7.4) containing 20 µM ThT (26.7 μL mL^−1^ of a 0.75 mM ThT stock solution in ddH_2_O). Three times, 125 μL of this solution was transferred into a 96-well plate (Corning^®^ 96-well half-area microplate, polystyrene with nonbinding surface coating, black, flat bottom clear). The dead time between preparation of the measuring solution and first measurement was < 5 min. Triplicates of **WT** and mutant Aβ_40_ peptides were measured. ThT fluorescence was measured with a microplate reader (Tecan Infinite M200, Tecan Group AG, Mannedorf, Switzerland) at 37 °C, 440 nm excitation wavelength, 482 nm emission detection, and shaking/5 min waiting cycle. Measurements were performed every 5 min for 48 h or every 30 min for 192 h (***N*-Me-L**).

### Crystal violet (CV) fluorescence measurements

Same procedure as for CV fluorescence measurements but using 5 µM CV (6.68 μL mL^−1^ of a 0.75 mM ThT stock solution in ddH_2_O) and 584 nm excitation wavelength, as well as 620 nm emission detection instead. Triplicates of **WT**, **F + 1/L + 1** and **F + 2/L + 2** were measured, but only qualitatively evaluated.

### Fluorescence intensity data evaluation

Fluorescence intensity data were normalized by $I= \frac{\left({I}_{t}-{I}_{min}\right)}{{I}_{max}}$ where $I$ is the normalized intensity, ${I}_{t}$ is the measured intensity value at the corresponding time point, and ${I}_{min}$ and ${I}_{max}$ are the minimal and maximal intensities measured for the corresponding well, respectively. Normalized data were fitted using a simple sigmoidal curve model^75^, $I= {y}_{i}+\frac{{y}_{f}}{1+{e}^{-\frac{\left(t-{t}_{0}\right)}{\tau }}}$, for the ThT fluorescence intensities, where $I$ is the normalized intensity, $t$ is the time, ${t}_{0}$ is the time at half maximal intensity, and $\tau$ is a measure for the fibrillation time. The lag time was derived by *t*_lag_ = *t*_0_ − 2τ and the fibrillation time by *t*_fib_ = 4τ. Reported mean values and standard deviations were calculated from the different fit values. Values discussed in the text for fibril formation kinetics are given as mean ± one standard deviation.

### Transmission electron microscopy (TEM)

A volume of 2 μL of a diluted fibril solution from the final state of ThT measurements (1:20 (v/v) with ddH_2_O) was transferred onto a formvar film-coated copper grid. After evaporation of the solvent, the sample was stained with 1% uranyl acetate. Images were recorded on an electron microscope (Zeiss SIGMA) equipped with a STEM detector and operated with Atlas software (Zeiss NTS, Oberkochen, Germany). The diameters of the fibrils were measured (n = 100) with the free of charge program ImageJ 1.53e^76^. Values discussed in the text for fibril diameters are given as mean ± one standard deviation.

### X-ray diffraction measurements

Mature fibrils from solid-state NMR samples were taken out of the MAS rotor after measurement. Oil was used to stick the powdered/crystalline samples to nylon loops which were mounted on a Rigaku XtaLAB Synergy Custom X-ray crystallography system (Rigaku, Tokyo, Japan) equipped with a copper MicroMax-007 HF microfocus rotating anode source (Cu K_α_ radiation) and a Hybrid Photon Counting (HPC) X-ray detector (HyPix-Arc 150°). Diffraction images were recorded with 120 s exposure time at room temperature (+ 18 °C). The collected data was analyzed with the powder diffraction tool of the CrysAlis Pro software package. Inter-β-strand and inter-β-sheet distances were calculated using Bragg’s law (λ = 1.5406 Å): $d=\frac{n \cdot \lambda }{2 \cdot {\sin}\theta }$.

### Solid-state NMR measurements

After fibrillation, the peptide solution was centrifuged at 63,000×*g* for 2 h at 4 °C. After removal of the supernatant, the pellets were frozen in liquid nitrogen and lyophilized for 72 h using a VaCo 2 lyophilizer (Zirbus technology GmbH, Bad Grund, Germany). For NMR measurements, the fibril pellets were rehydrated to 50% (w/w) ddH_2_O. For homogenization, at least 10 freeze/thaw cycles were applied (liquid nitrogen − 37 °C water bath/centrifugation at 16,000×*g* for 3 s) and the sample was transferred into 3.2 mm MAS rotors. Solid-state MAS NMR spectra were recorded on a 600 MHz Avance III NMR spectrometer (Bruker BioSpin GmbH, Rheinstetten, Germany) operated at resonance frequencies of 600.1, 150.9, and 60.8 MHz for ^1^H, ^13^C, and ^15^N, respectively. Chemical shifts were given for ^13^C relative to tetramethylsilane (TMS) and for ^15^N relative to liquid NH_3_. A triple channel 3.2 mm MAS probe operated at a temperature of 30 °C was used for all experiments. The 90° pulse length was set to 4 μs for ^1^H in ^13^C or ^15^N experiments. For cross-polarization (CP), a contact time of 1 ms and a spin lock field of ca. 50 kHz were used. Relaxation delay was set to 2.5 s and the Spinal64^77^ (small phase incremental alternation with 64 steps) sequence with a radio frequency (rf) amplitude of 62.5 kHz was applied for ^1^H decoupling. Two-dimensional NMR spectra were recorded using either a dual acquisition pulse sequence^78^, where one ^13^C–^13^C DARR^79^ and four to eight ^15^N–^13^Cα correlation spectra were acquired simultaneously, or a single ^13^C–^13^C DARR experiment. The mixing time for the DARR experiments was 500 ms (labeling scheme 1), 50 or 600 ms (labeling scheme 2), MAS frequency was set to 11,777 Hz, and double CP contact times were 1 ms for ^1^H–^15^N and 4 ms for ^13^C–^15^N transfer steps. To determine the motional averaged ^1^H–^13^C dipolar couplings, constant time DipShift experiments with 5 kHz MAS frequency and ca. 80 kHz effective radio frequency field frequency switched Lee–Goldburg homonuclear decoupling^80^ were performed^81^. Using numerical simulation of the experimental dephasing curves over one rotor period, motional averaged dipolar couplings were determined. For DipShift time domain data simulation a C++ program was used. Euler angles for powder averaging were incremented in 1° steps in the simulations. The best fit of the dephasing curve to the experimental data was used to determine motional averaged dipolar coupling values ⟨*δ*_CH_⟩, from which the order parameters *S*_CH_ were calculated according to *S*_CH_ = ⟨*δ*_CH_⟩/*δ*_CH_, where *δ*_CH_ is the dipolar coupling rigid limit value derived frozen amino acid samples^82,83^.

### Cell culture

Primary neuronal cultures were established from fetal mouse brains of C57Bl/6 mice at gestation day 16 and grown under standard conditions^84,85^. Briefly, fetal brains were prepared, and neurons were dissociated into single cells by triturating the brains by means of pipets and passing the cell suspensions through sterile nylon meshes (150 µm and 20 μm). Suspensions were then grown in seeding medium (DMEM/Ham’s F-12 supplemented with 5% (v/v) fetal horse serum (FHS) and 1% (v/v) penicillin–streptomycin–neomycin (PSN) antibiotic mixture) in 96-well culture plates (for MTT and LDH assays) or on poly-l-lysine-coated glass coverslips in 24-well culture plates (for immunocytochemistry), respectively. The cells were cultured at 37 °C in a humidified atmosphere containing 5% (v/v) CO_2_. On the following day, the seeding medium was exchanged by neuronal medium (Dulbecco’s Modified Eagle Medium/Nutrient Mixture F-12 (DMEM/Ham’s F-12) supplemented with 1% (v/v) PSN, 1% (v/v) N-2 supplement and 20% (v/v) astrocyte conditioned medium). After 3 days in vitro, cultures were stimulated with Aβ_40_ peptides at concentrations of 10 μM for 18 h.

Neuronal survival was analyzed using the 3-(4,5-dimethylthiazol-2-yl)-2,5-diphenyltetrazolium bromide (MTT) assay which measures glycolytic activity. Briefly, MTT was dissolved in cell culture media without serum and added to the primary neurons for 2 h. After addition of dimethyl sulfoxide (DMSO)/EtOH (1:1, v/v) and shaking for 10 min, the absorbance was measured at 592 nm (Mithras LB 940, Berthold Technologies).

Neuronal cell death was measured in cell culture supernatant by lactate dehydrogenase (LDH) assay (Promega, CytoTox96) according to the manufacturer’s protocol. LDH is a cytosolic protein, which is not detectable extracellularly under normal conditions, but only in the case of membrane leakage.

### Immunocytochemistry

Cells grown on coverslips were stained with primary antibodies against activated caspase-3 (1:100, AF835, R&D Systems) and neurofilament M and H (1:1000, 171,104, 171,204, Synaptic Systems) in Tris-buffered saline (TBS) (0.1 M, pH 7.4) containing 0.1% (v/v) Triton X-100 and 5% (v/v) normal donkey serum at 4 °C overnight. On the next day, coverslips were washed thrice with TBS and then incubated with the respective secondary antibodies donkey anti-rabbit-Cy3 (1:400 (v/v), 711-225-152, Dianova), donkey anti-guinea pig-Cy2 (1:400 (v/v), Dianova) in TBS containing 2% (v/v) bovine serum albumin (BSA, Serva Electrophoresis GmbH) for 60 min at room temperature. After rinsing with TBS, cell nuclei were stained with Hoechst 33342 in TBS (1:10,000 (v/v)) for 10 min at room temperature. The coverslips were washed, air-dried, embedded with Entellan^®^/toluene on glass slides, and stored at 4 °C in the dark.

Stained coverslips were examined with an AxioScan.Z1 microscope (Carl Zeiss, Göttingen, Germany). Emission filters were selected for Cy2 (524 nm), for Cy3 (595 nm) and Hoechst 33342 (425 nm). Images were taken by means of Axiocam 506 and a Plan-Apochromat objective (20×/0.8) and digitized using ZEN 3.4 software. For quantification of neurite lengths, regions of interest were digitized by means of Keyence microscope BZ9000 and lengths were measured using BZ II Analyzer 2.1 software.

Control experiments were performed by omitting the primary antibody. All controls were negative, indicating that the observed fluorescence signals were specific. For quantification, ten regions of interest from each coverslip were randomly selected and mean intensity values of activated caspase-3 and Hoechst 33342 signals were determined.

## Supplementary Information

Supplementary Information.
