# Supplementary material for: Peptide backbone modifications of amyloid β (1–40) impact fibrillation behavior and neuronal toxicity
Source: Sci Rep. 2021 Dec 9;11:23767. doi: 10.1038/s41598-021-03091-4 (PMC8660793; doi:10.1038/s41598-021-03091-4)
Supplement: Supplementary file 1 — Supplementary Information. [file 41598_2021_3091_MOESM1_ESM.pdf]

Supporting Information

Peptide Backbone Modifications of Amyloid β (1-40) Impact Fibrillation Behavior and Neuronal Toxicity

Benedikt Schwarze[1], Alexander Korn[1], Corinna Höfling[2], Ulrike Zeitschel[2], Martin Krueger[3], Steffen Roßner[2], and Daniel Huster[1,*]

1 Institute for Medical Physics and Biophysics, Leipzig University, Härtelstr. 16/18, 04107 Leipzig, Germany

2 Paul Flechsig Institute for Brain Research, Leipzig University, Liebigstr. 19, 04103 Leipzig, Germany

3 Institute of Anatomy, Leipzig University, Liebigstr. 13, 04103 Leipzig, Germany

Correspondence: daniel.huster@medizin.uni-leipzig.de

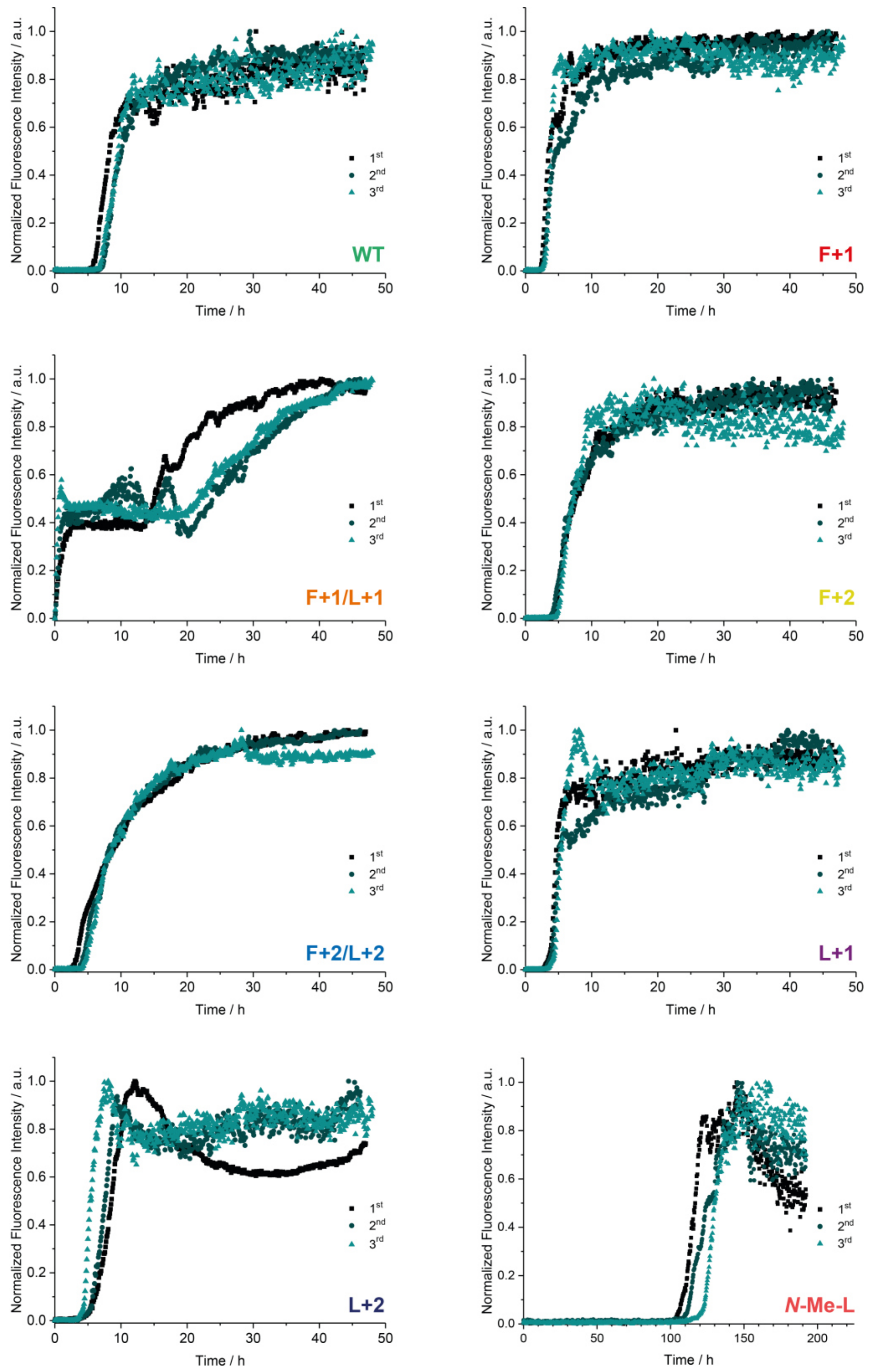

Figure S1. *(Normalized) ThT fluorescence intensities as a function of fibrillation time in h of each Aβ peptide mutant. Shown are exemplarily the fibrillation curves of three samples in one of the three repeated experiments.*

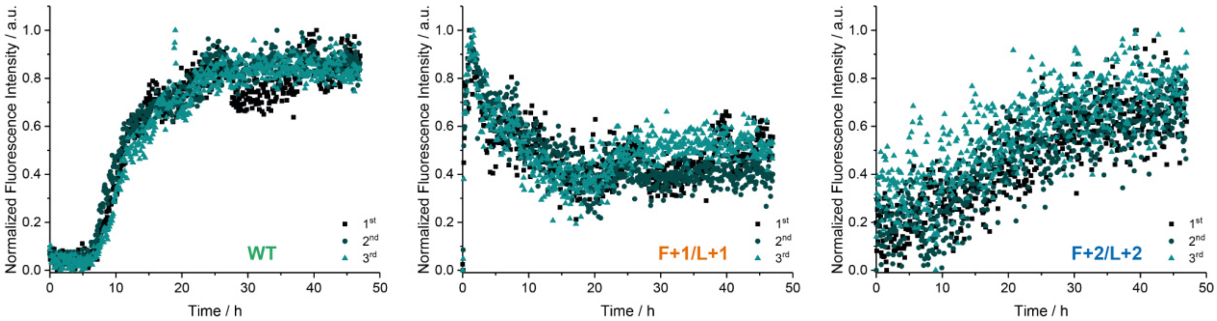

***Figure S2**. (Normalized) CV fluorescence intensities as a function of fibrillation time in h of **WT**, **F+1/L+1** and **F+2/L+2**. Shown are exemplarily the fibrillation curves of three samples in one of the three repeated experiments.*

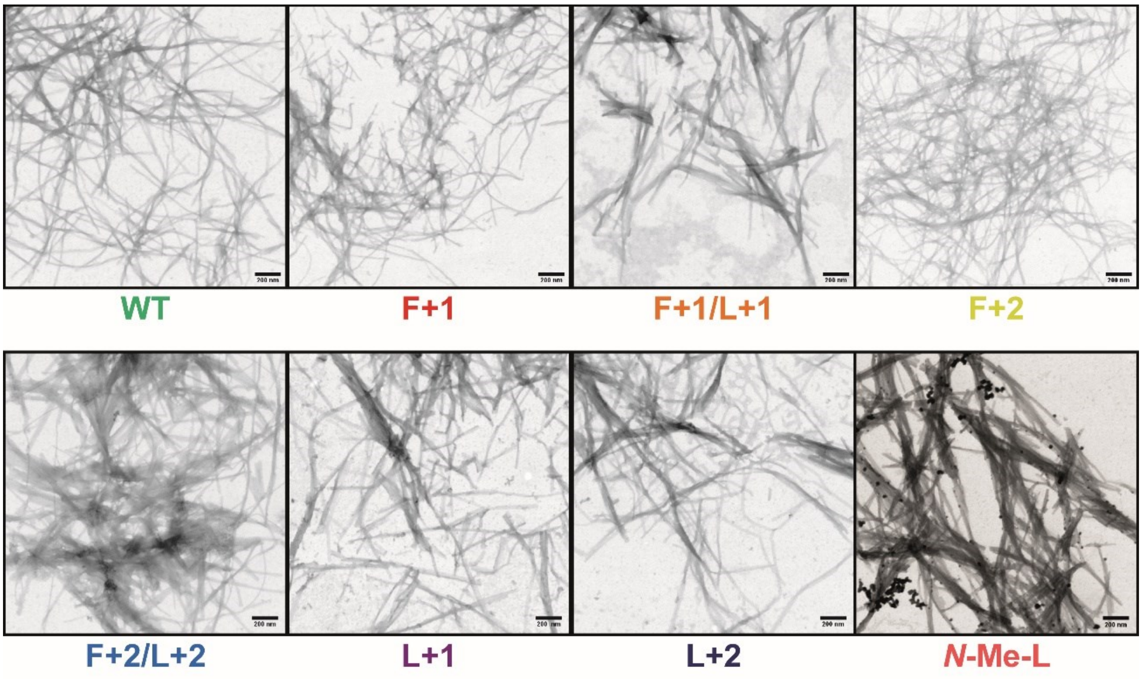

Figure S3. *TEM images of all the mutants which fibrillated according ThT fluorescence assay. For* ***N-Me-F*** *and* ***N-Me-F/L*** *no fibrils were found in the TEM images. The scale bar represents 200 nm.*

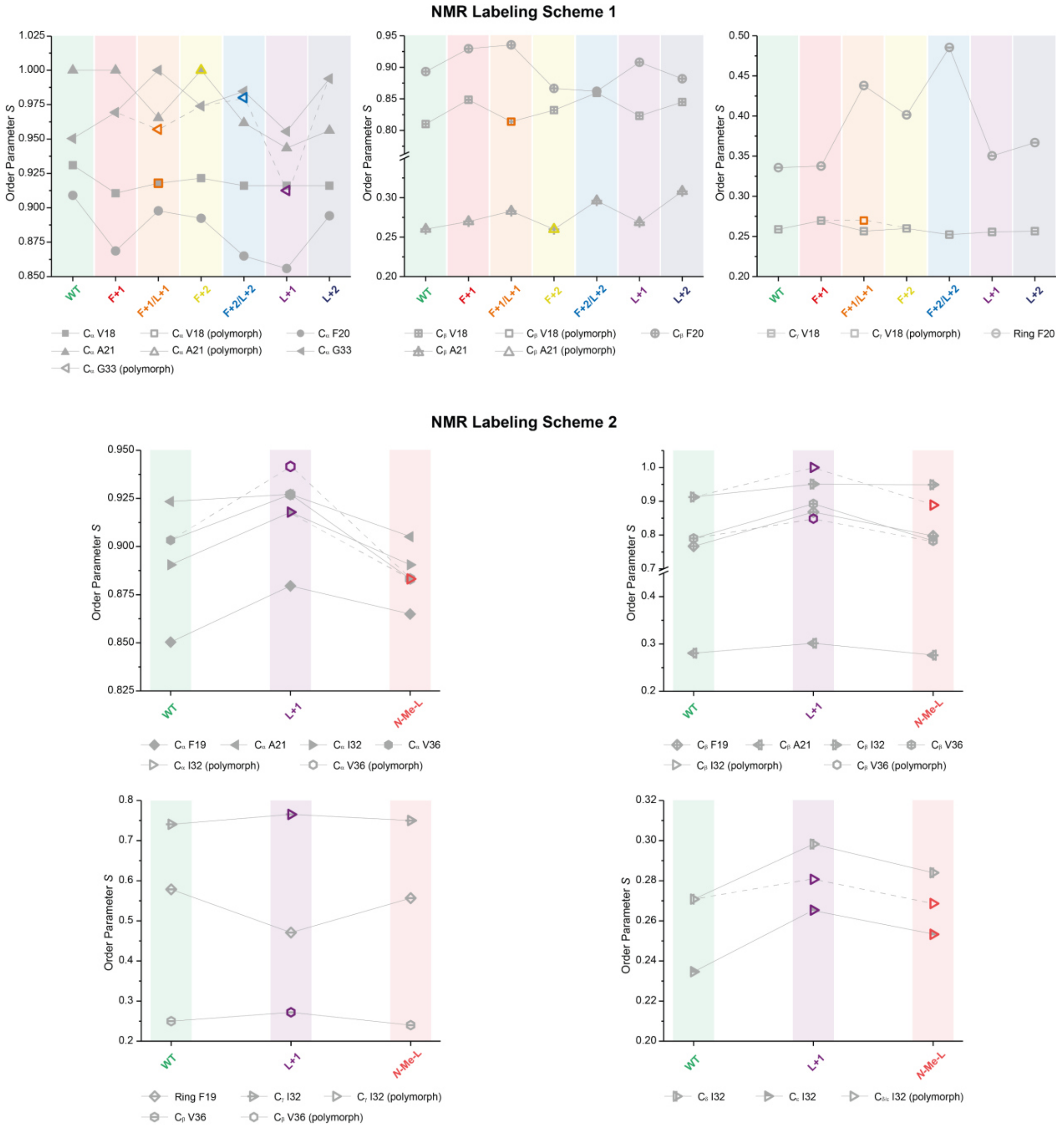

Figure S4. *Order parameters derived from solid-state NMR DipShift experiments representing the amplitude of the molecular motions of the respective bond vector. A value of 1 connotes absolute rigidity, where 0 means isotropic motion. Values for* C_α*,* C_β*, and other side chain carbons are plotted separately for clarity. Error bars were estimated as 10% of (1−S) but omitted in the representation for clarity. Polymorphs, when present, are highlighted in the color of the type of* $A\beta_{40}$*.*

Table S1. *Summary for ^{13}C (relative to TMS) and ^{15}N (relative to liquid NH_3) chemical shifts and order parameters S for all investigated fibrils determined MAS NMR spectroscopy at 303 K for labeling scheme 1. The standard deviation for order parameter S determination is assumed to be 10%. Some signals could not be determined (n.d.). Values for* ***WT*** *derived from evaluation of the raw data in Korn et al.* [1].

Scheme 1	**Residue**	**C_α / ppm**	***S***	**C_β / ppm**	***S***	**C_γ / ppm**	***S***	**C_ε / ppm**	***S***	**CO / ppm**	**N /ppm**
**WT**	V18	58.9	0.93	33.3	0.81	19.2	0.26	---	---	170.7	121.4
(Korn *et al.*[1])	F20	54.2	0.91	40.6	0.89	---	---	129.3	0.33	171.3	123.5
	A21	47.7	1.00	20.7	0.26	---	---	---	---	173.2	126.1
	G33	42.6	0.95	---	---	---	---	---	---	168.6	114.8
**F+1**	V18	58.9	0.91	32.6	0.85	19.2	0.27	---	---	171.2	121.6
	F20	55.3	0.87	40.0	0.93	---	---	129.4	0.34	172.7	123.5
	A21	48.0	1.00	19.9	0.27	---	---	---	---	173.3	126.7
	G33	43.4	0.97	---	---	---	---	---	---	n.d.	114.8
**F+1/L+1**	V18_1	59.2	0.92	32.5	0.81	19.2	0.26	---	---	171.5	121.3
	V18_2	59.2	0.92	32.5	0.81	22.5	0.27	---	---	171.5	121.3
	F20	56.9	0.90	39.1	0.94	---	---	129.9	0.44	172.6	119.4
	A21	48.2	0.97	19.6	0.28	---	---	---	---	173	126.6
	G33_1	43.8	1.00	---	---	---	---	---	---	n.d.	113.0
	G33_2	42.0	0.96	---	---	---	---	---	---	n.d.	113.0
**F+2**	V18	58.9	0.92	32.9	0.83	19.0	0.26	---	---	171.2	121.8
	F20	54.3	0.89	39.8	0.87	---	---	129.5	0.40	173.4	124.6
	A21_1	47.0	1.00	19.9	0.26	---	---	---	---	173.1	124.6
	A21_2	48.0	1.00	20.1	0.26	---	---	---	---	173.1	124.6
	G33	42.9	0.97	---	---	---	---	---	---	n.d.	114.9
**F+2/L+2**	V18	59.0	0.92	32.0	0.86	18.6	0.25	---	---	171.4	122.7
	F20	53.8	0.86	39.9	0.86	---	---	129.5	0.49	172	114.0
	A21	48.3	0.96	21.6	0.30	---	---	---	---	173.3	118.7
	G33_1	43.6	0.98	---	---	---	---	---	---	n.d.	112.3
	G33_2	46.3	0.98	---	---	---	---	---	---	n.d.	115.7
**L+1**	V18	58.9	0.92	32.1	0.82	19.4	0.26	---		171.5	123.0
	F20	54.3	0.86	33.4	0.91	---	---	129.3	0.35	173.9	125.4
	A21	47.9	0.94	21.6	0.27	---	---	---	---	173.3	125.0
	G33_1	43.9	0.96	---	---	---	---	---	---	n.d.	111.8

	G33_2	41.7	0.91	---	---	---	---	---	---	n.d.	111.8
**L+2**	V18	59.1	0.92	33.1	0.84	19.0	0.26	---	---	170.9	n.d.
	F20	54.1	0.89	39.0	0.88	---	---	129.3	0.37	172.3	n.d.
	Ala 21	48.1	0.96	20.0	0.31	---	---	---	---	173.2	n.d.
	Gly 33	43.3	0.99	---	---	---	---	---	---	170.4	n.d.

Table S2. *Summary for ^{13}C (relative to TMS) and ^{15}N (relative to liquid NH_3) chemical shifts and order parameters S for all investigated fibrils determined MAS NMR spectroscopy at 303 K for labeling scheme 2. The standard deviation for order parameter S determination is assumed to be 10%.*

Scheme 2	**Residue**	**C_α / ppm**	***S***	**C_β / ppm**	***S***	**C_γ / ppm**	***S***	**C_δ / ppm**	***S***	**C_ε / ppm**	***S***	**CO / ppm**
**WT**	F19	53.1	0.85	39.9	0.77	---	---	---	---	128.8	0.58	171.2
	A21	48	0.92	21.3	0.28	---	---	---	---	---	---	172.9
	I32	55.7	0.89	40.3	0.91	25.1	0.74	16.3	0.27	12.7	0.23	174.2
	V36	58.4	0.90	31.7	0.79	19.2	0.25	---	---	---	---	172.4
**L+1**	F19	54.1	0.88	40.2	0.87	---	---	---	---	129.6	0.47	171.3
	A21	48.1	0.93	21.5	0.30	---	---	---	---	---	---	173.4
	I32_1	57.9	0.92	37.2	0.95	25.9	0.77	13.6	0.30	11.9	0.27	172.2
	I32_2	57.9	0.92	39.9	1.03	25.9	0.77	16.3	0.28	12.4	0.27	172.2
	V36_1	58.4	0.93	33.6	0.89	19.5	0.27	---	---	---	---	173
	V36_2	59.3	0.94	31.6	0.85	19.5	0.27	---	---	---	---	173
***N*-Me-L**	F19	54.8	0.86	40.7	0.80	---	---	---	---	129.3	0.56	170.2
	A21	48.2	0.91	20.7	0.28	---	---	---	---	---	---	173.5
	I32_1	57.1	0.89	40.8	0.95	25.2	0.75	15.4	0.28	12.1	0.25	172.4
	I32_2	58.1	0.88	38.5	0.89	25.6	0.75	16	0.27	11.8	0.25	172.4
	V36	58.6	0.88	32.2	0.78	19	0.24	---	---	---	---	173.8

Assignment of sample names in chromatograms from analytical HPLC measurements and MALDI mass spectra to numbering in this manuscript

**Sample name**	**Numbering**
BS20	WT
BB20	F+1
CH20 (labeled)	F+1
BC20	F+2
CI20 (labeled	F+2
BF20	L+1
CJ20 (labeled)	L+1
BG20	L+2
CK20 (labeled)	L+2
CC20	F+1/L+1
DL20 (labeled)	F+1/L+1
CD20	F+2/L+2
DM20 (labeled)	F+2/L+2
A20 (labeled)	*N*-Me-F
B20 (labeled)	*N*-Me-F/L
CB20	*N*-Me-L
M21 (labeled, scheme 2)	WT
EC20 (labeled, scheme 2)	L+1
N21 (labeled, scheme 2)	*N*-Me-L

353 IZKF Leipzig, Core Unit Peptid-Technologien
Liebigstraße 21, 04103 Leipzig, e-mail: sven_r@yahoo.com, Tel.: 0341 - 9715898 / 897

Comment:

Sample Name:	**BS20 purified**	*Injection Volume:*	**10.0**
Vial Number:	**BE3**	*Channel:*	**UV_VIS_1**
Sample Type:	**unknown**	*Wavelength:*	**220.0**
Control Program:	**Peptide_3D_basic_short**	*Bandwidth:*	**4**
Quantif. Method:	**Peptide_3D_basic**	*Dilution Factor:*	**1.0000**
Recording Time:	**20/5/2020 11:45**	*Operator:*	**KEYUSER**
Sample ID:		*Sample Amount:*	**1.0000**

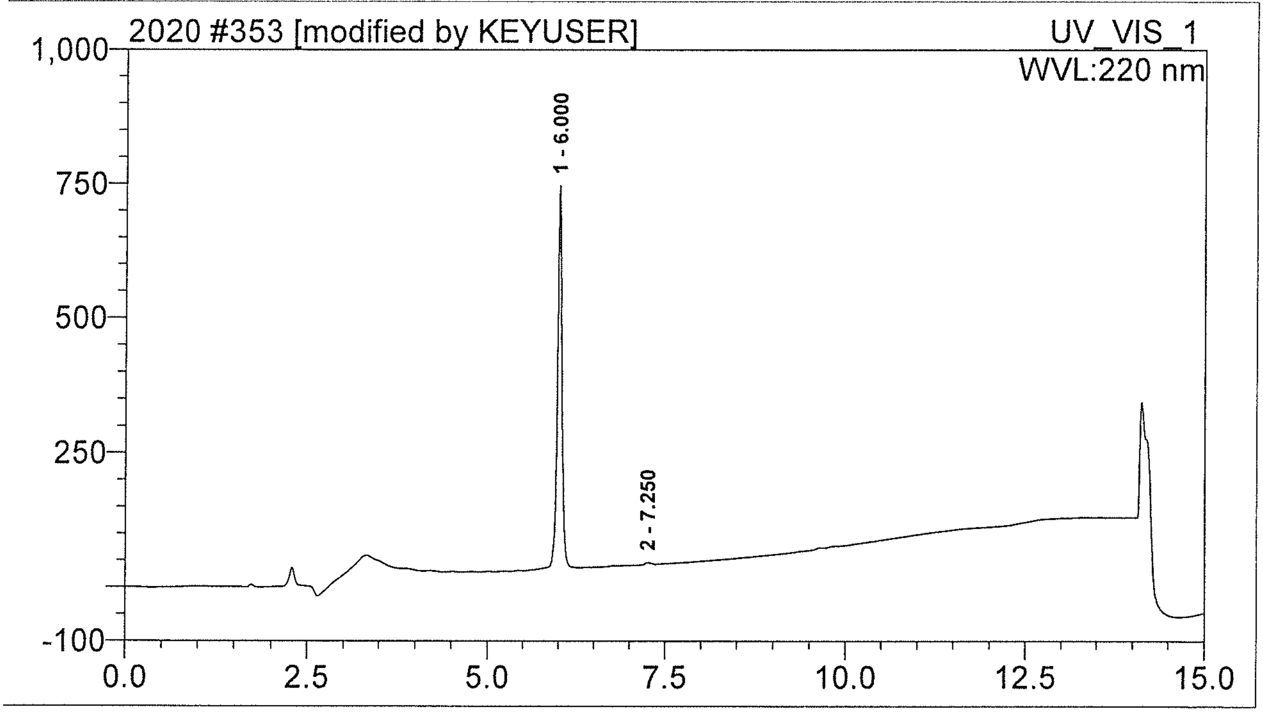

No.	Ret.Time min	Peak Name	Height mAU	Rel.Area %	Area mAU*min	Amount	Type
1	6.00	n.a.	711.2	99.47	53.18	n.a.	BMB*
2	7.25	n.a.	3.8	0.53	0.28	n.a.	BMB*
Total:			715.016	100.000	53.47	0.000	

Dr. Sven Rothemund, IZKF Leipzig, Liebigstrasse 21, 04103 Leipzig, Tel.: 0341-9715898, sven_r@yahoo.com

Comment 1 BS20_purified
Comment 2 MW:av4329,9(M+H)+

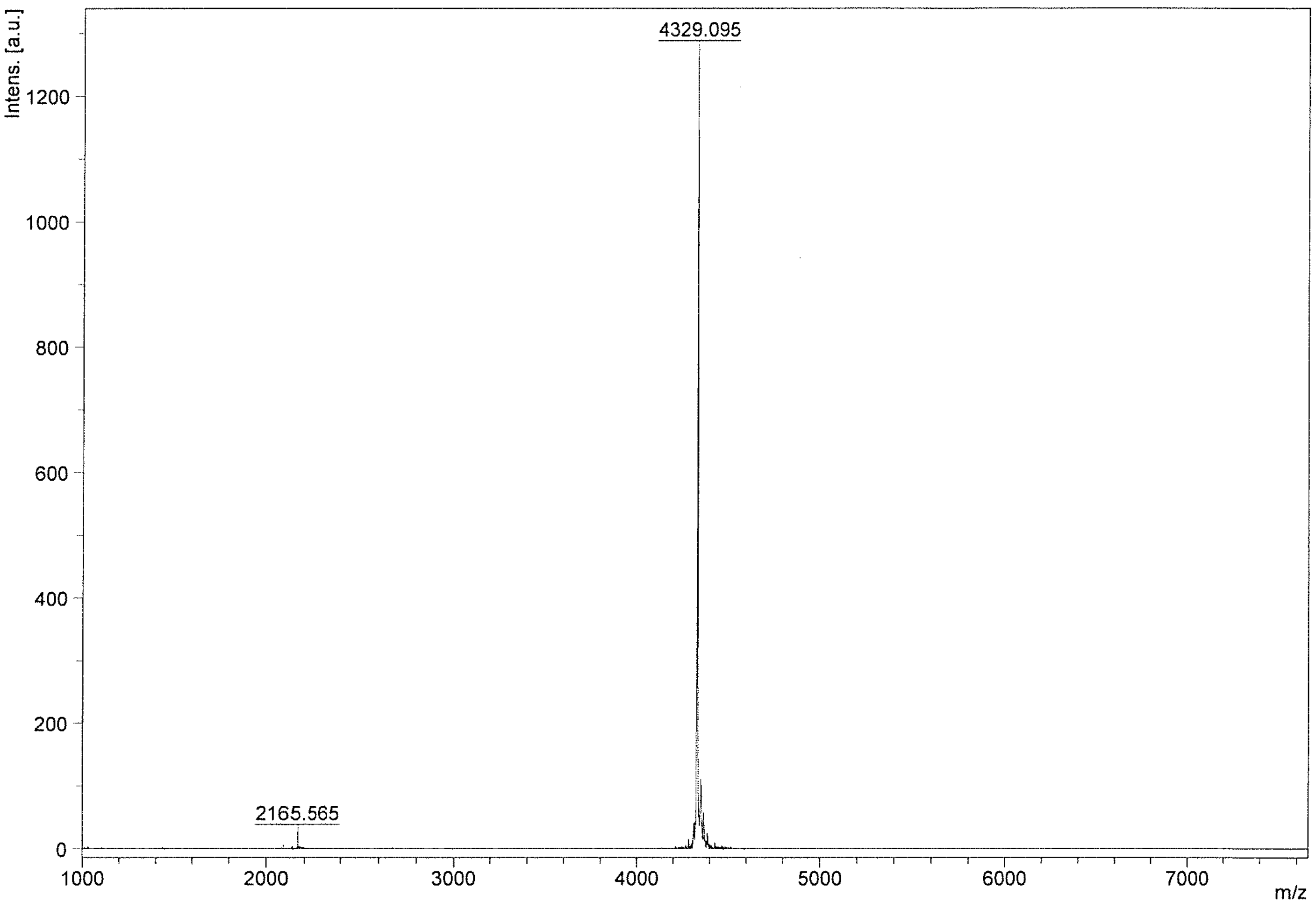

Acquisition Parameter

Date of acquisition	2020-05-20T13:21:31.222+02:00
Acquisition method name	D:\Methods\flexControlMethods\LP_PepMix.par
Aquisition operation mode	Linear
Voltage polarity	POS
Number of shots	44
Name of spectrum used for calibration	
Calibration reference list used	PeptideCalibStandardInsulin2 monoAv

Instrument Info

User	IZKF
Instrument	FLEX-PC

287 IZKF Leipzig, Core Unit Peptid-Technologien
Liebigstraße 21, 04103 Leipzig, e-mail: sven_r@yahoo.com, Tel.: 0341 - 9715898 / 897

Comment:

Sample Name:	**BB20 purif 2**	*Injection Volume:*	**20.0**
Vial Number:	**BE2**	*Channel:*	**UV_VIS_1**
Sample Type:	**unknown**	*Wavelength:*	**220.0**
Control Program:	**Peptide_3D_basic_short**	*Bandwidth:*	**4**
Quantif. Method:	**peptide_izkf**	*Dilution Factor:*	**1.0000**
Recording Time:	**28/3/2020 14:20**	*Operator:*	**KEYUSER**
Sample ID:		*Sample Amount:*	**1.0000**

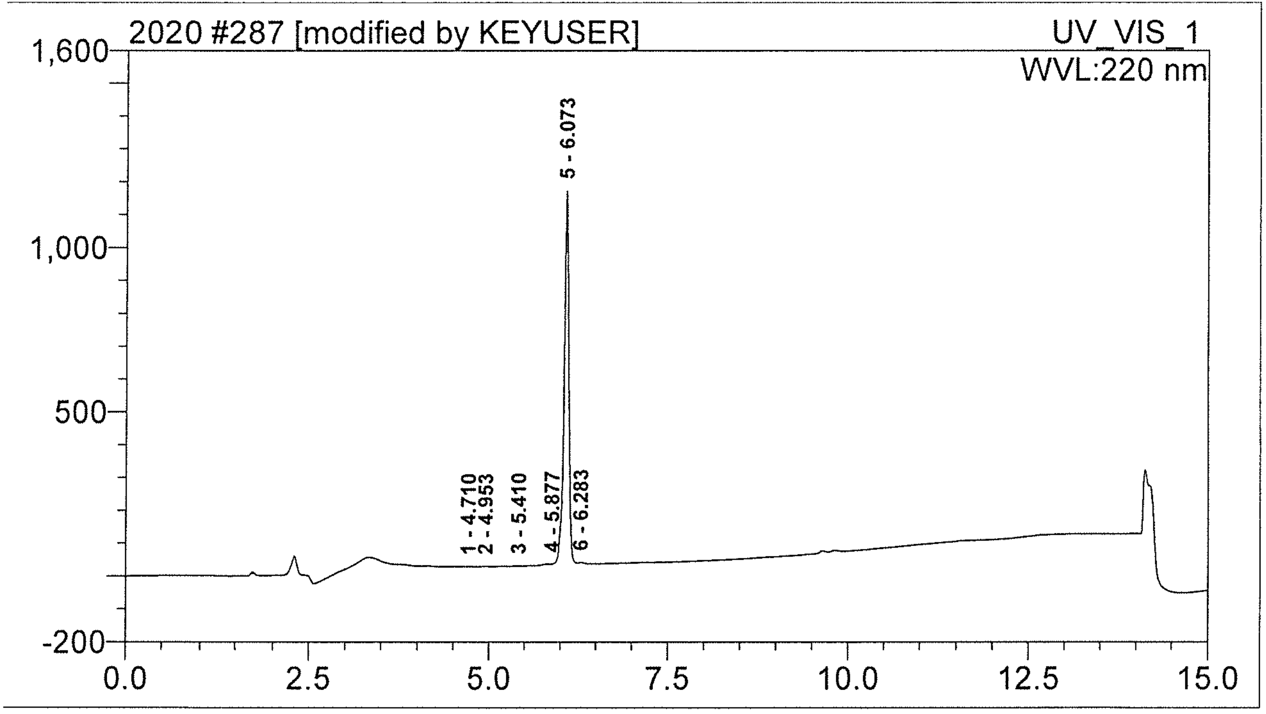

No.	Ret.Time min	Peak Name	Height mAU	Rel.Area %	Area mAU*min	Amount	Type
1	4.71	n.a.	0.8	0.10	0.09	n.a.	BMB
2	4.95	n.a.	0.8	0.10	0.09	n.a.	BMB
3	5.41	n.a.	0.6	0.08	0.07	n.a.	BMB
4	5.88	n.a.	3.3	0.51	0.46	n.a.	BM
5	6.07	n.a.	1138.1	98.52	88.65	n.a.	M
6	6.28	n.a.	5.9	0.69	0.62	n.a.	MB
**Total:**			1149.518	100.000	89.98	0.000	

Chromeleon (c) Dionex 1996-2006
Version 6.80 SR15 Build 4656 (243203)

Dr. Sven Rothemund, IZKF Leipzig, Liebigstrasse 21, 04103 Leipzig, Tel.: 0341-9715898, sven_r@yahoo.com

Comment 1	BB20 purif
Comment 2	MW: 4343,2 (M+H)+

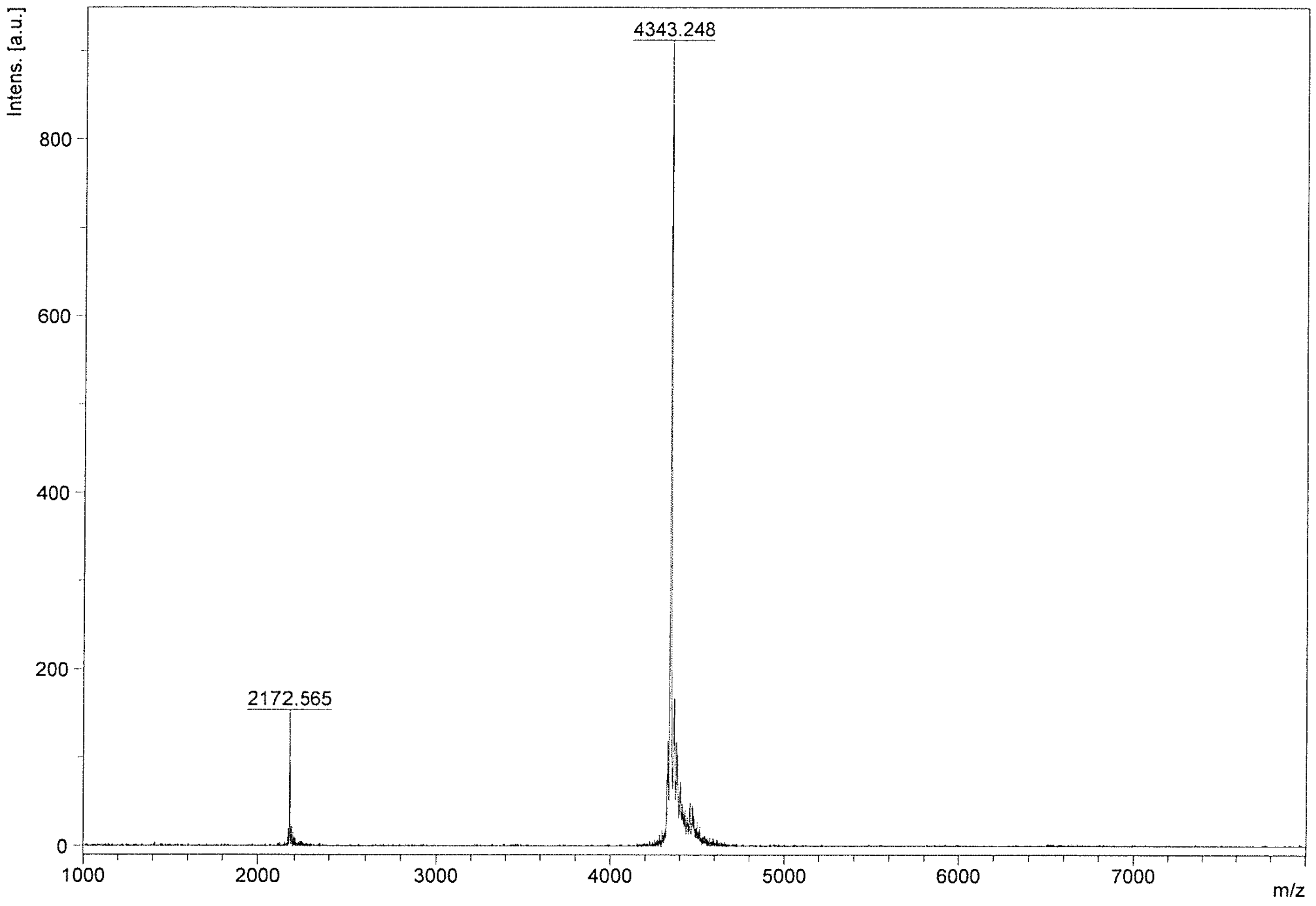

Acquisition Parameter

Date of acquisition	2020-03-28T14:24:04.131+01:00
Acquisition method name	D:\Methods\flexControlMethods\LP_PepMix.par
Aquisition operation mode	Linear
Voltage polarity	POS
Number of shots	35
Name of spectrum used for calibration	
Calibration reference list used	PeptideCalibStandardInsulin2 monoAv

Instrument Info

User	IZKF
Instrument	FLEX-PC

D:\Data\IZKF2020\BB20 purif\0_D3\1 printed: 28.03.2020 14:24:51

423 **IZKF Leipzig, Core Unit Peptid-Technologien**
Liebigstraße 21, 04103 Leipzig, e-mail: sven_r@yahoo.com, Tel.: 0341 - 9715898 / 897

Comment:

Sample Name:	**CH20_purified**	*Injection Volume:*	**20.0**
Vial Number:	**GE1**	*Channel:*	**UV_VIS_1**
Sample Type:	**unknown**	*Wavelength:*	**220.0**
Control Program:	**Peptide_3D_basic_short**	*Bandwidth:*	**4**
Quantif. Method:	**Peptide_3D_basic**	*Dilution Factor:*	**1.0000**
Recording Time:	**2/7/2020 11:51**	*Operator:*	**KEYUSER**
Sample ID:		*Sample Amount:*	**1.0000**

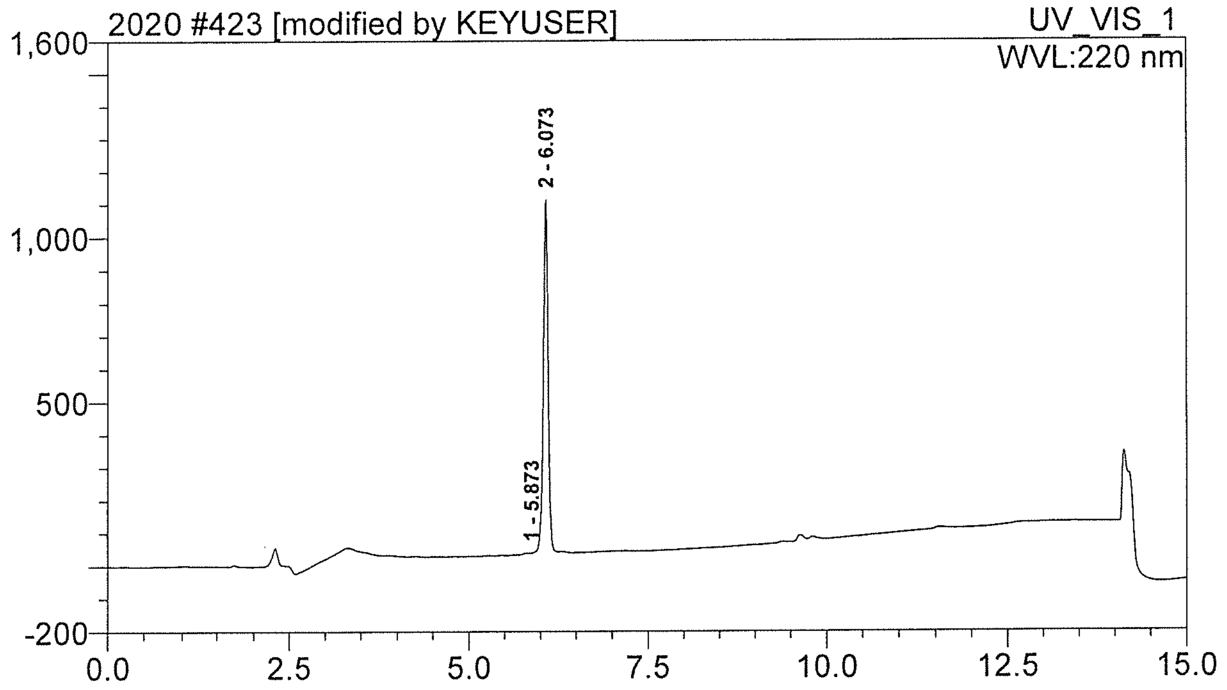

No.	Ret.Time min	Peak Name	Height mAU	Rel.Area %	Area mAU*min	Amount	Type
1	5.87	n.a.	1.9	0.37	0.31	n.a.	BM *
2	6.07	n.a.	1072.9	99.63	82.52	n.a.	MB*
**Total:**			1074.797	100.000	82.83	0.000	

Chromeleon (c) Dionex 1996-2006
Version 6.80 SR15 Build 4656 (243203)

Dr. Sven Rothemund, IZKF Leipzig, Liebigstrasse 21, 04103 Leipzig, Tel.: 0341-9715898, sven_r@yahoo.com

Comment 1 CH20_purified
Comment 2 MW:4366,2(M+H)+

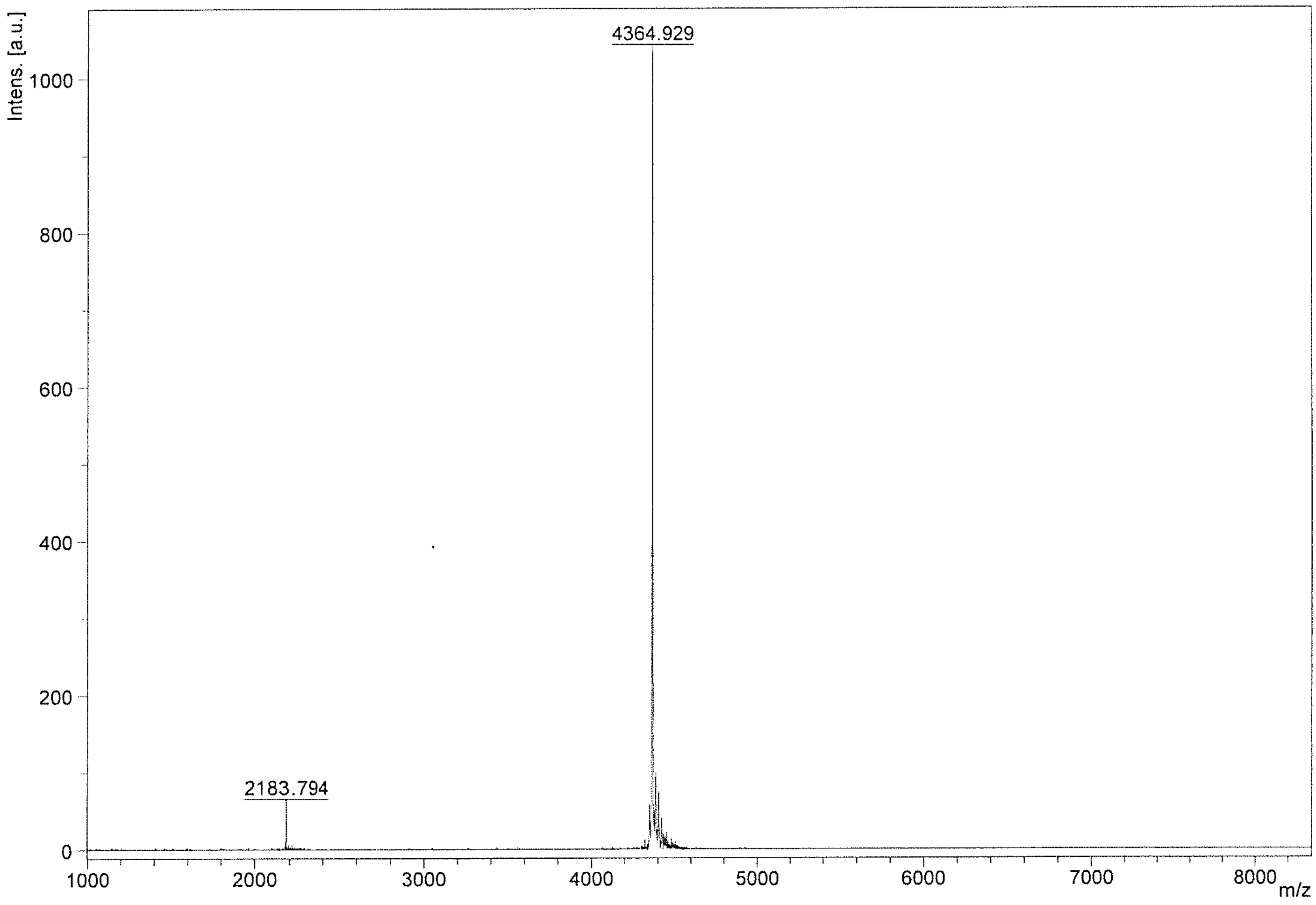

Acquisition Parameter

Date of acquisition	2020-07-02T11:53:29.503+02:00
Acquisition method name	D:\Methods\flexControlMethods\LP_PepMix.par
Aquisition operation mode	Linear
Voltage polarity	POS
Number of shots	67
Name of spectrum used for calibration	
Calibration reference list used	PeptideCalibStandardInsulin2 monoAv

Instrument Info

User	IZKF
Instrument	FLEX-PC

D:\Data\IZKF2020\CH20_purified\0_E4\1 printed: 02.07.2020 11:54:47

293 **IZKF Leipzig, Core Unit Peptid-Technologien**

Liebigstraße 21, 04103 Leipzig, e-mail: sven_r@yahoo.com, Tel.: 0341 - 9715898 / 897

Comment:

Sample Name:	**BC20 purif**	*Injection Volume:*	**20.0**
Vial Number:	**BE3**	*Channel:*	**UV_VIS_1**
Sample Type:	**unknown**	*Wavelength:*	**220.0**
Control Program:	**Peptide_3D_basic_short**	*Bandwidth:*	**4**
Quantif. Method:	**peptide_izkf**	*Dilution Factor:*	**1.0000**
Recording Time:	**31/3/2020 14:02**	*Operator:*	**KEYUSER**
Sample ID:		*Sample Amount:*	**1.0000**

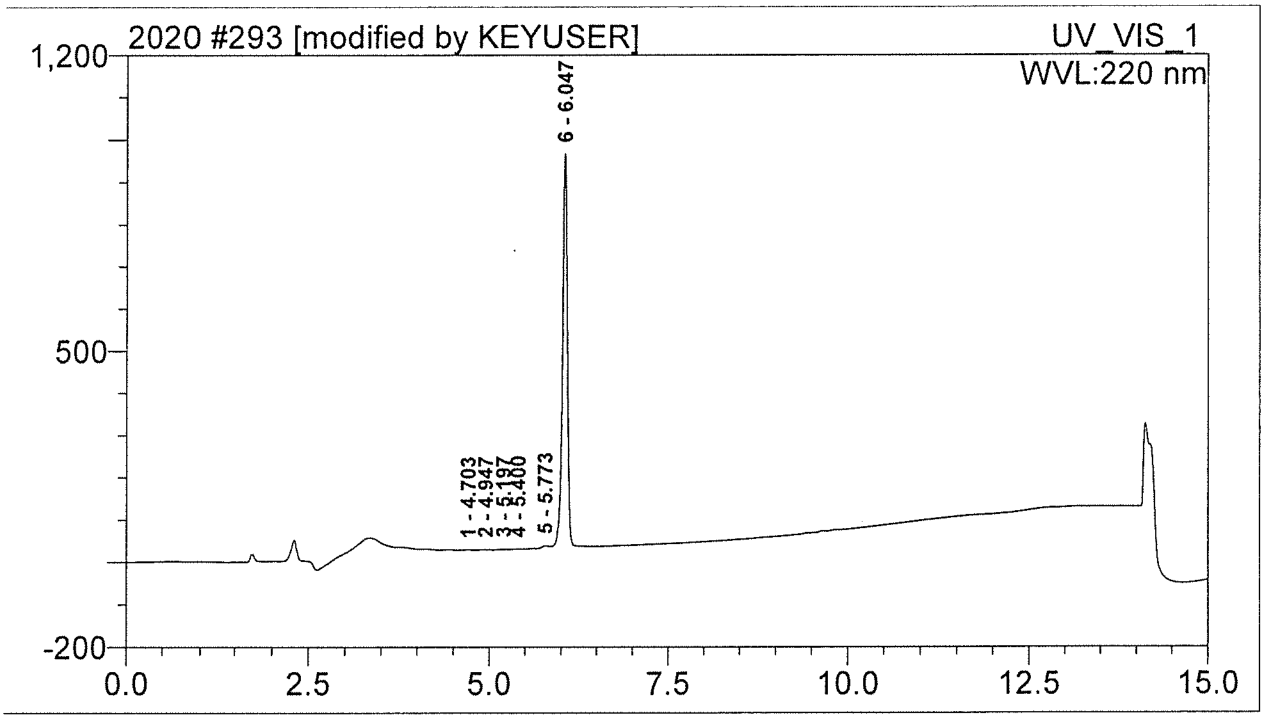

No.	Ret.Time min	Peak Name	Height mAU	Rel.Area %	Area mAU*min	Amount	Type
1	4.70	n.a.	0.8	0.12	0.09	n.a.	BMB
2	4.95	n.a.	0.8	0.13	0.09	n.a.	BMB
3	5.20	n.a.	0.7	0.10	0.08	n.a.	BMB
4	5.40	n.a.	0.6	0.10	0.08	n.a.	BMB
5	5.77	n.a.	6.1	0.86	0.63	n.a.	BM
6	6.05	n.a.	932.2	98.68	72.28	n.a.	MB
**Total:**			941.204	100.000	73.25	0.000	

Chromeleon (c) Dionex 1996-2006
Version 6.80 SR15 Build 4656 (243203)

Dr. Sven Rothemund, IZKF Leipzig, Liebigstrasse 21, 04103 Leipzig, Tel.: 0341-9715898, sven_r@yahoo.com

Comment 1	BC20 purif
Comment 2	MW: 4357 (M+H)+

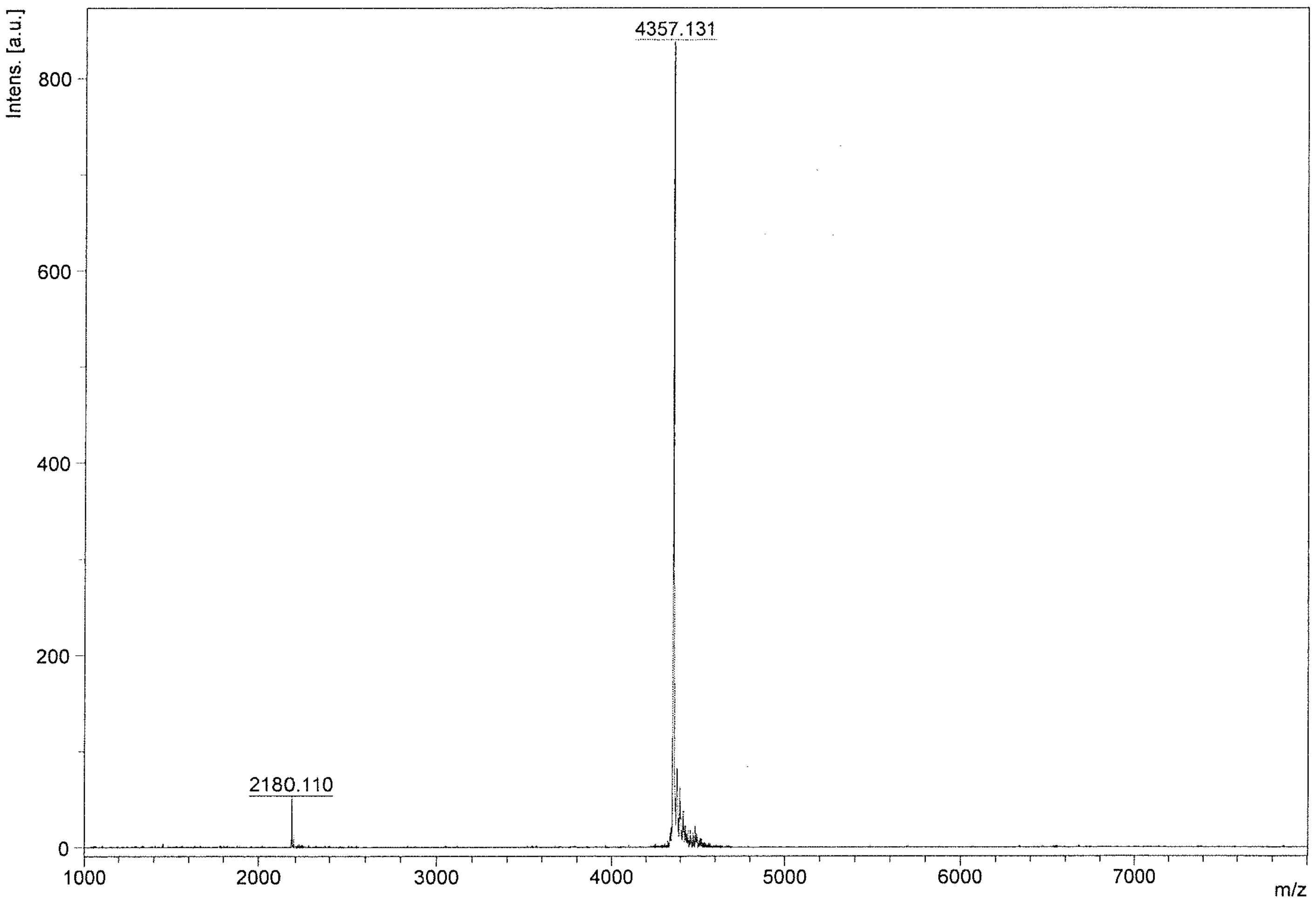

Acquisition Parameter

Date of acquisition	2020-03-31T13:38:55.742+02:00
Acquisition method name	D:\Methods\flexControlMethods\LP_PepMix.par
Aquisition operation mode	Linear
Voltage polarity	POS
Number of shots	24
Name of spectrum used for calibration	
Calibration reference list used	PeptideCalibStandardInsulin2 monoAv

Instrument Info

User	IZKF
Instrument	FLEX-PC

D:\Data\IZKF2020\BC20 purif\0_D4\1 printed: 31.03.2020 13:39:16

428 **IZKF Leipzig, Core Unit Peptid-Technologien**
Liebigstraße 21, 04103 Leipzig, e-mail: sven_r@yahoo.com, Tel.: 0341 - 9715898 / 897

Comment: Fr.21-27

Sample Name:	**Cl20_purified**	*Injection Volume:*	**20.0**
Vial Number:	**BA2**	*Channel:*	**UV_VIS_1**
Sample Type:	**unknown**	*Wavelength:*	**220.0**
Control Program:	**Peptide_3D_basic_short**	*Bandwidth:*	**4**
Quantif. Method:	**peptide_izkf**	*Dilution Factor:*	**1.0000**
Recording Time:	**6/7/2020 12:20**	*Operator:*	**KEYUSER**
Sample ID:		*Sample Amount:*	**1.0000**

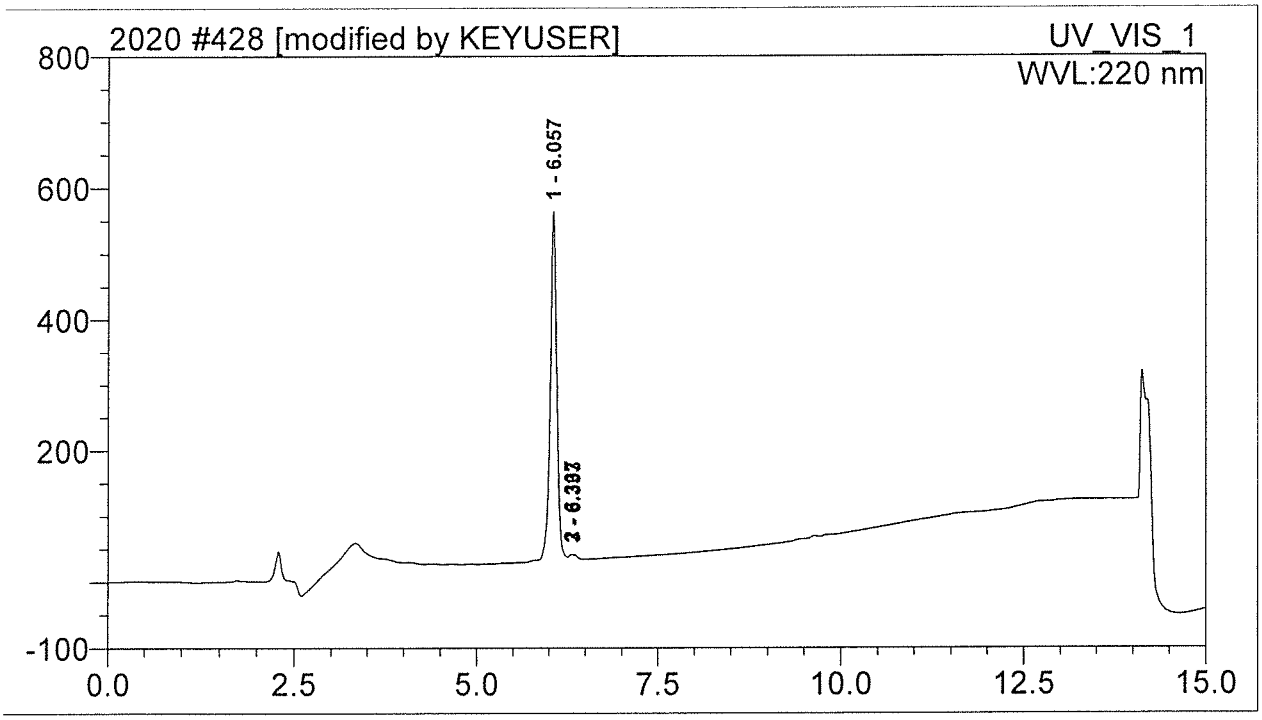

No.	Ret.Time min	Peak Name	Height mAU	Rel.Area %	Area mAU*min	Amount	Type
1	6.06	n.a.	530.1	98.27	55.32	n.a.	BM
2	6.31	n.a.	8.7	1.61	0.91	n.a.	MB
3	6.33	n.a.	0.1	0.12	0.07	n.a.	Rd
**Total:**			538.859	100.000	56.29	0.000	

Chromeleon (c) Dionex 1996-2006
Version 6.80 SR15 Build 4656 (243203)

Dr. Sven Rothemund, IZKF Leipzig, Liebigstrasse 21, 04103 Leipzig, Tel.: 0341-9715898, sven_r@yahoo.com

Comment 1 CI20_purified_Fr.21-27
Comment 2 MW:4380,2(M+H)+

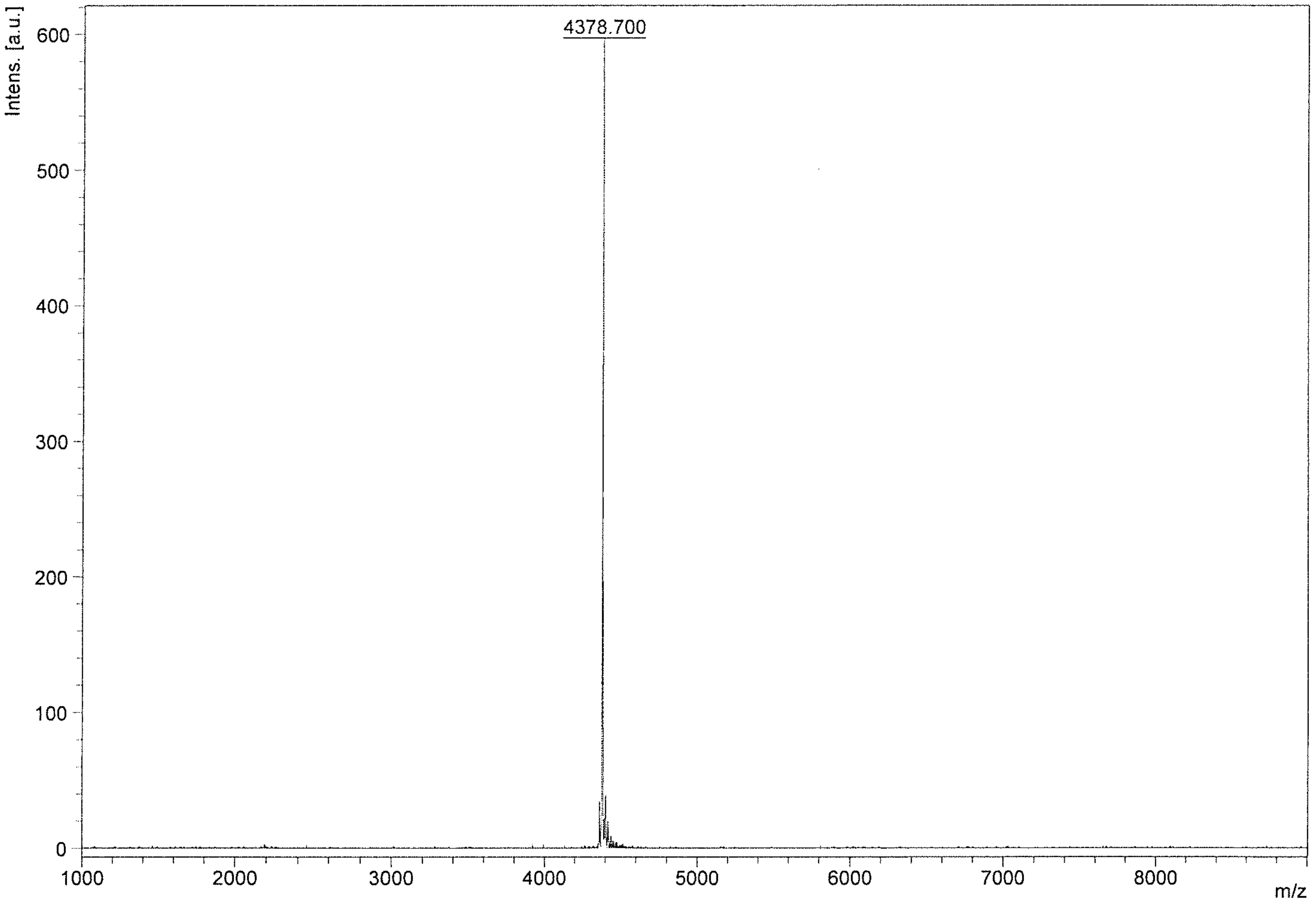

Acquisition Parameter

Date of acquisition	2020-07-06T12:40:30.881+02:00
Acquisition method name	D:\Methods\flexControlMethods\LP_PepMix.par
Aquisition operation mode	Linear
Voltage polarity	POS
Number of shots	100
Name of spectrum used for calibration	
Calibration reference list used	PeptideCalibStandardInsulin2 monoAv

Instrument Info

User	IZKF
Instrument	FLEX-PC

D:\Data\IZKF2020\CI20_purified_Fr.21-27\0_C7\1 printed: 06.07.2020 12:41:10

303 **IZKF Leipzig, Core Unit Peptid-Technologien**
Liebigstraße 21, 04103 Leipzig, e-mail: sven_r@yahoo.com, Tel.: 0341 - 9715898 / 897

Comment: Fr.10-15

Sample Name:	**BF20_purified**	*Injection Volume:*	**20.0**
Vial Number:	**BB2**	*Channel:*	**UV_VIS_1**
Sample Type:	**unknown**	*Wavelength:*	**220.0**
Control Program:	**Peptide_3D_basic_short**	*Bandwidth:*	**4**
Quantif. Method:	**peptide_izkf**	*Dilution Factor:*	**1.0000**
Recording Time:	**21/4/2020 11:59**	*Operator:*	**KEYUSER**
Sample ID:		*Sample Amount:*	**1.0000**

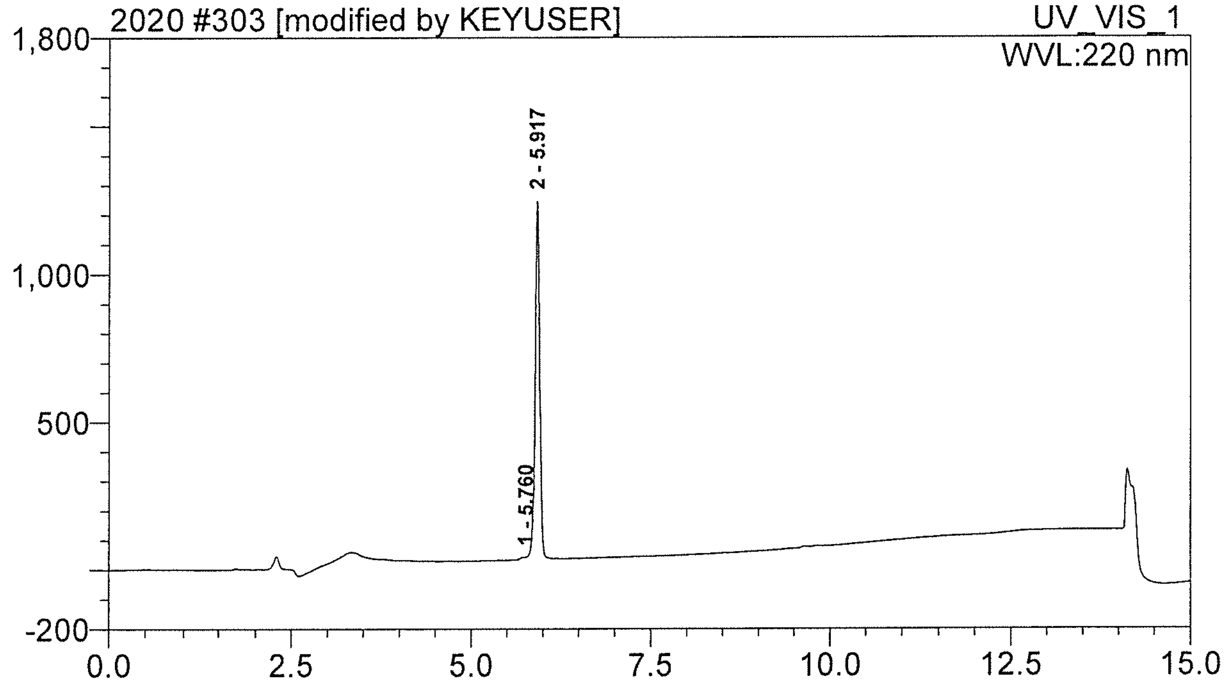

No.	Ret.Time min	Peak Name	Height mAU	Rel.Area %	Area mAU*min	Amount	Type
1	5.76	n.a.	8.0	0.72	0.65	n.a.	BM
2	5.92	n.a.	1210.3	99.28	90.07	n.a.	MB
Total:			1218.257	100.000	90.72	0.000	

Dr. Sven Rothemund, IZKF Leipzig, Liebigstrasse 21, 04103 Leipzig, Tel.: 0341-9715898, sven_r@yahoo.com

Comment 1	BF20_purified_Fr.10-15
Comment 2	MW:av.4343,1(M+H)+

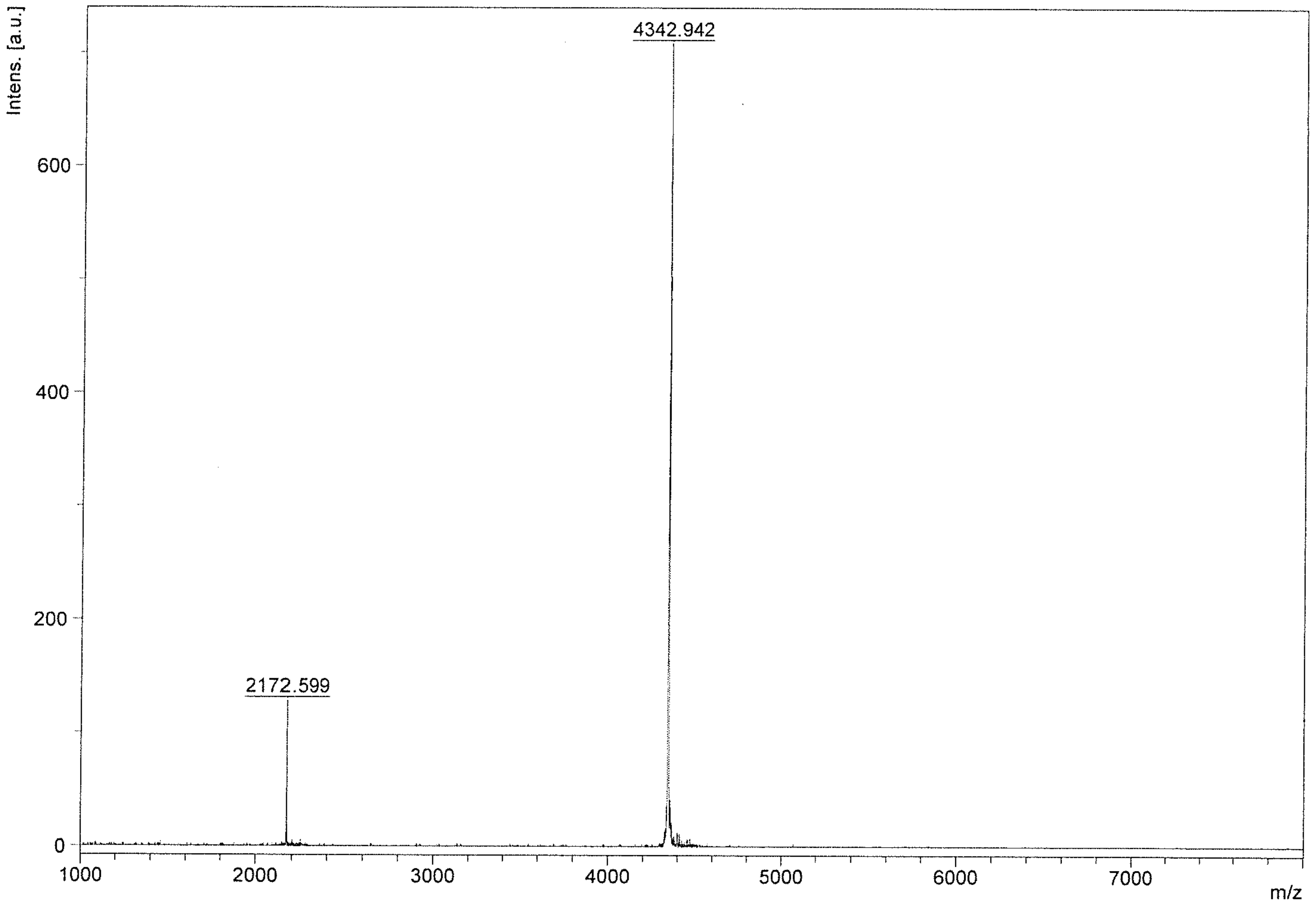

Acquisition Parameter

Date of acquisition	2020-04-21T12:28:28.336+02:00
Acquisition method name	D:\Methods\flexControlMethods\LP_PepMix.par
Aquisition operation mode	Linear
Voltage polarity	POS
Number of shots	29
Name of spectrum used for calibration	
Calibration reference list used	PeptideCalibStandardInsulin2 monoAv

Instrument Info

User	IZKF
Instrument	FLEX-PC

446 IZKF Leipzig, Core Unit Peptid-Technologien
Liebigstraße 21, 04103 Leipzig, e-mail: sven_r@yahoo.com, Tel.: 0341 - 9715898 / 897

Comment: Fr 12-19

Sample Name:	**CJ20 purified**	*Injection Volume:*	**20.0**
Vial Number:	**BD2**	*Channel:*	**UV_VIS_1**
Sample Type:	**unknown**	*Wavelength:*	**220.0**
Control Program:	**Peptide_3D_basic_short**	*Bandwidth:*	**4**
Quantif. Method:	**peptide_izkf**	*Dilution Factor:*	**1.0000**
Recording Time:	**7/7/2020 12:25**	*Operator:*	**KEYUSER**
Sample ID:		*Sample Amount:*	**1.0000**

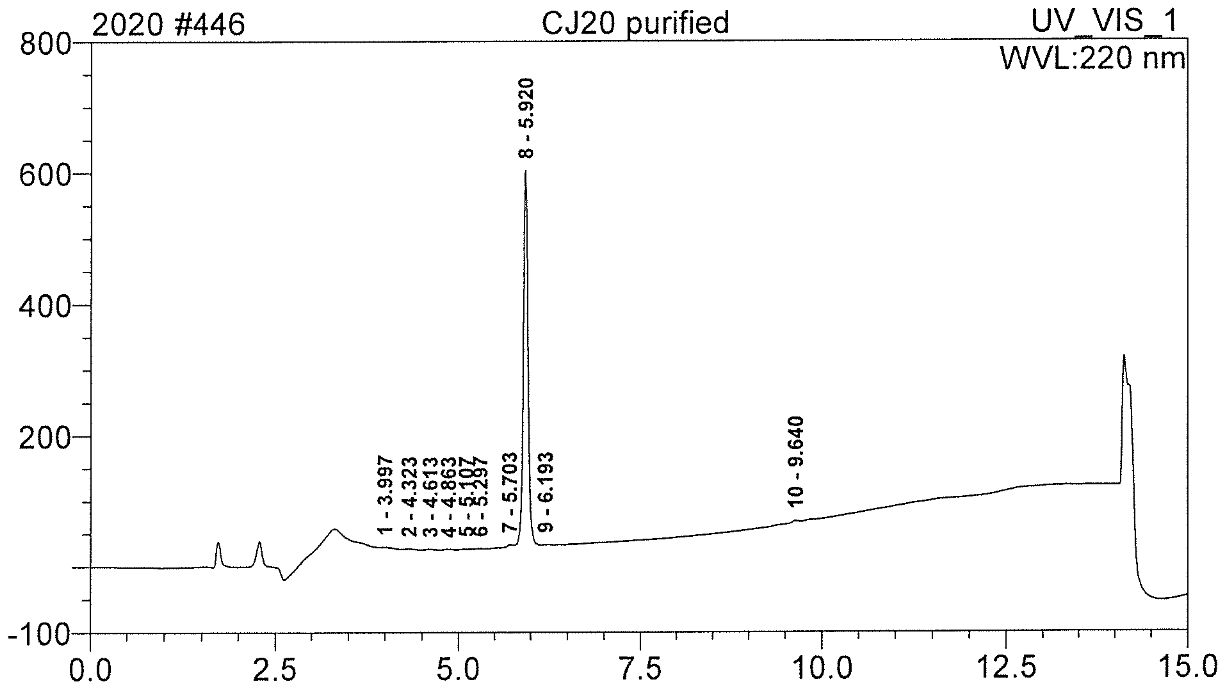

No.	Ret.Time min	Peak Name	Height mAU	Rel.Area %	Area mAU*min	Amount	Type
1	4.00	n.a.	1.1	0.40	0.18	n.a.	BMB
2	4.32	n.a.	0.8	0.25	0.11	n.a.	BMB
3	4.61	n.a.	1.0	0.27	0.12	n.a.	BMB
4	4.86	n.a.	0.8	0.20	0.09	n.a.	BMB
5	5.11	n.a.	0.7	0.16	0.07	n.a.	BMB
6	5.30	n.a.	0.9	0.20	0.09	n.a.	bMB
7	5.70	n.a.	4.0	0.76	0.34	n.a.	BM
8	5.92	n.a.	572.0	96.89	43.12	n.a.	M
9	6.19	n.a.	1.3	0.34	0.15	n.a.	MB
10	9.64	n.a.	2.6	0.52	0.23	n.a.	BMB
**Total:**			585.056	100.000	44.51	0.000	

Chromeleon (c) Dionex 1996-2006
Version 6.80 SR15 Build 4656 (243203)

Dr. Sven Rothemund, IZKF Leipzig, Liebigstrasse 21, 04103 Leipzig, Tel.: 0341-9715898, sven_r@yahoo.com

Comment 1 CJ20 purified Fr 12-19
Comment 2 MW:4366,1(M+H)+

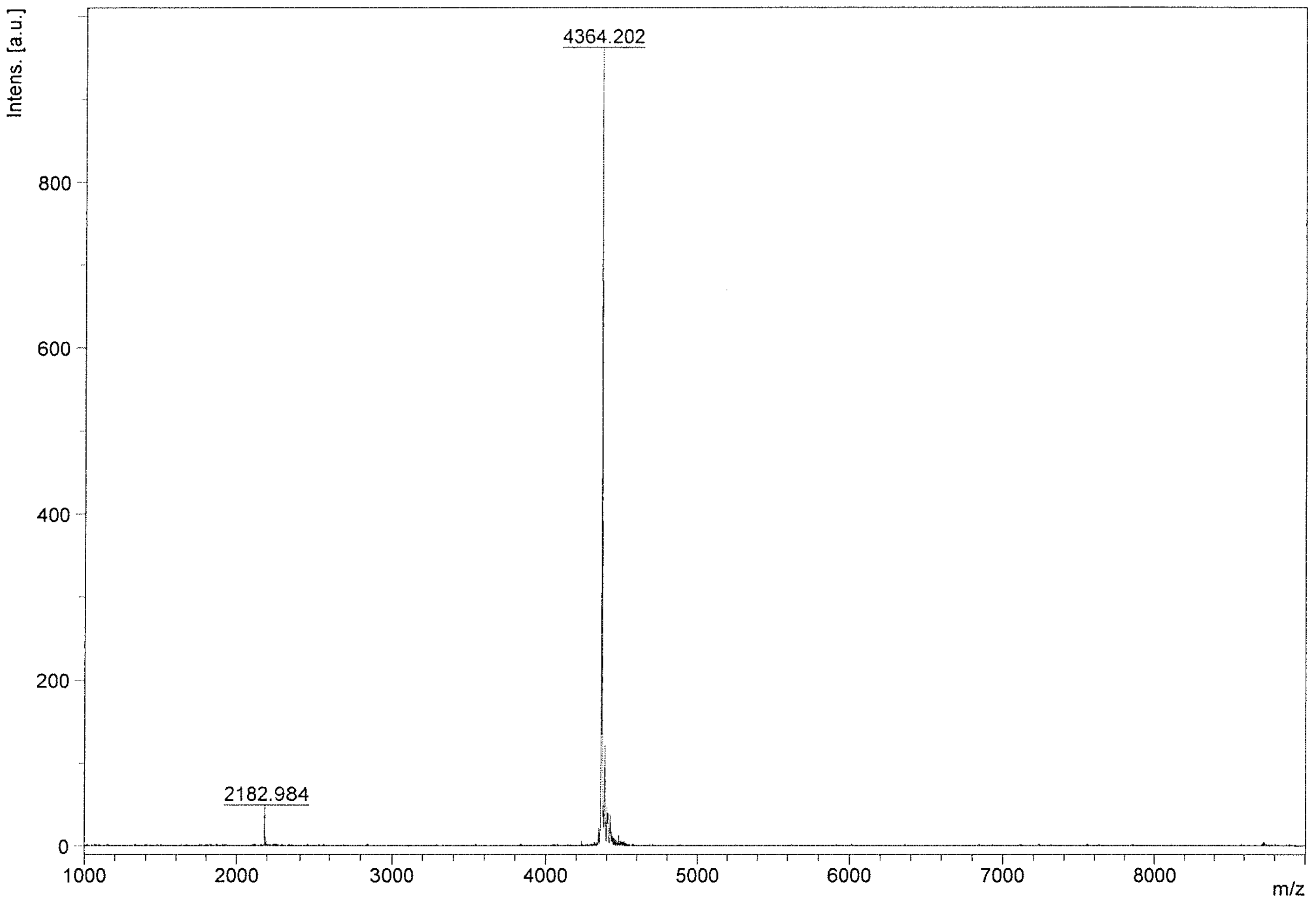

Acquisition Parameter

Date of acquisition	2020-07-07T12:07:05.533+02:00
Acquisition method name	D:\Methods\flexControlMethods\LP_PepMix.par
Aquisition operation mode	Linear
Voltage polarity	POS
Number of shots	100
Name of spectrum used for calibration	
Calibration reference list used	PeptideCalibStandardInsulin2 monoAv

Instrument Info

User	IZKF
Instrument	FLEX-PC

306 IZKF Leipzig, Core Unit Peptid-Technologien

Liebigstraße 21, 04103 Leipzig, e-mail: sven_r@yahoo.com, Tel.: 0341 - 9715898 / 897

Comment: Fr.38-42

Sample Name:	**BG20_purified**	*Injection Volume:*	**20.0**
Vial Number:	**BB4**	*Channel:*	**UV_VIS_1**
Sample Type:	**unknown**	*Wavelength:*	**220.0**
Control Program:	**Peptide_3D_basic_short**	*Bandwidth:*	**4**
Quantif. Method:	**Peptide_3D_basic**	*Dilution Factor:*	**1.0000**
Recording Time:	**21/4/2020 14:13**	*Operator:*	**KEYUSER**
Sample ID:		*Sample Amount:*	**1.0000**

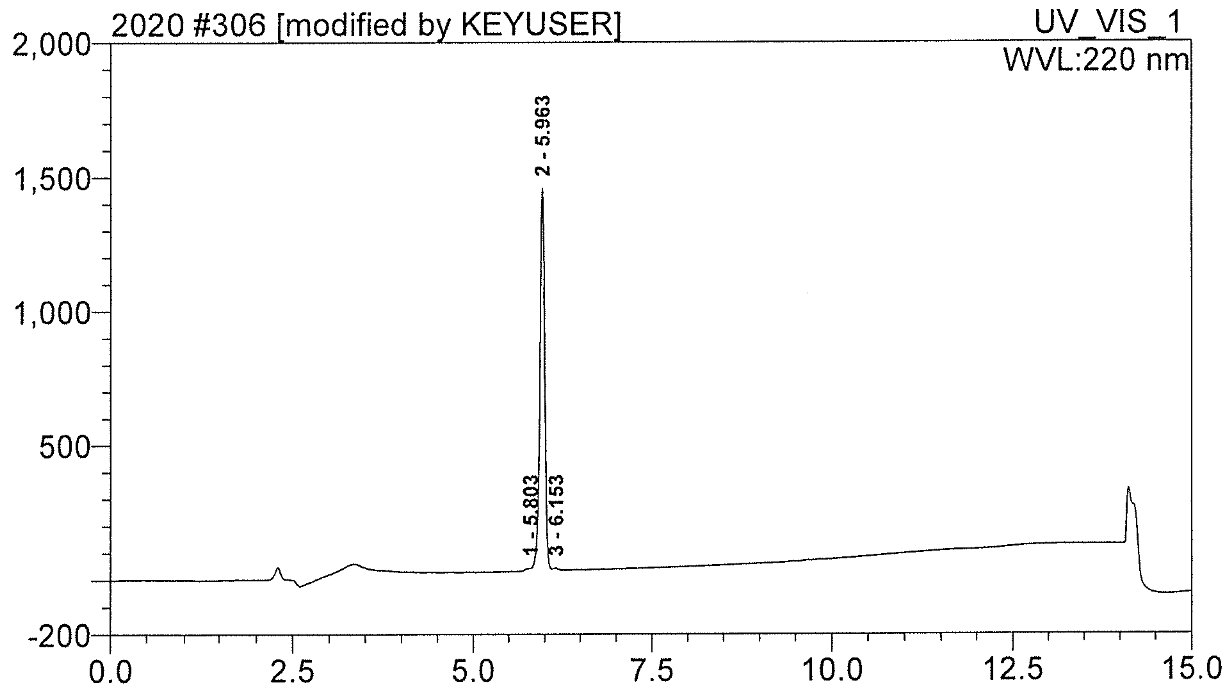

No.	Ret.Time min	Peak Name	Height mAU	Rel.Area %	Area mAU*min	Amount	Type
1	5.80	n.a.	9.9	0.67	0.75	n.a.	BM *
2	5.96	n.a.	1422.3	98.74	110.00	n.a.	M *
3	6.15	n.a.	8.7	0.58	0.65	n.a.	MB*
**Total:**			1440.903	100.000	111.40	0.000	

Chromeleon (c) Dionex 1996-2006
Version 6.80 SR15 Build 4656 (243203)

Dr. Sven Rothemund, IZKF Leipzig, Liebigstrasse 21, 04103 Leipzig, Tel.: 0341-9715898, sven_r@yahoo.com

Comment 1	BG20_purified_Fr.38-42
Comment 2	MW:av.4357,2(M+H)+

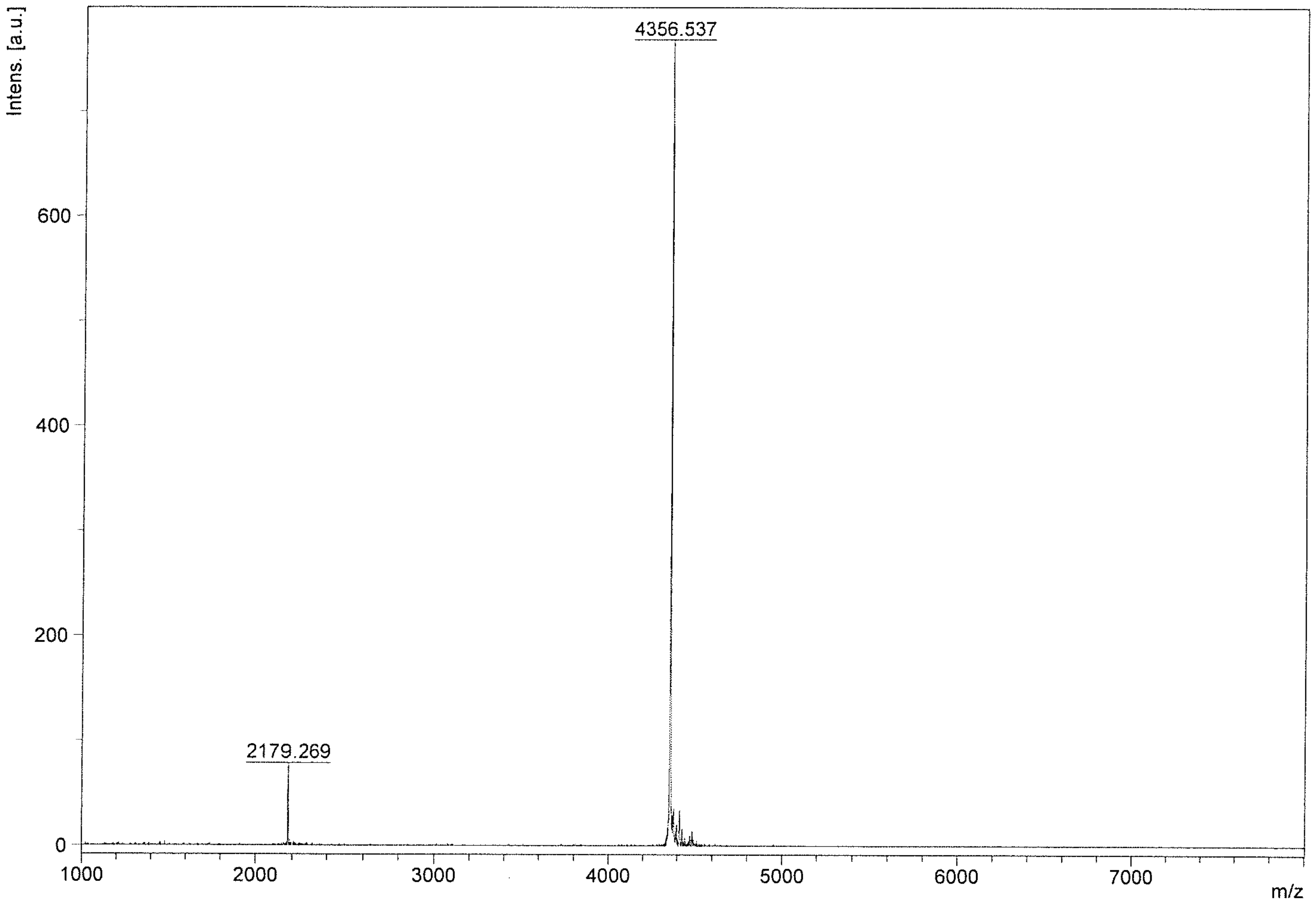

Acquisition Parameter

Date of acquisition	2020-04-21T14:17:37.069+02:00
Acquisition method name	D:\Methods\flexControlMethods\LP_PepMix.par
Aquisition operation mode	Linear
Voltage polarity	POS
Number of shots	36
Name of spectrum used for calibration	
Calibration reference list used	PeptideCalibStandardInsulin2 monoAv

Instrument Info

User	IZKF
Instrument	FLEX-PC

D:\Data\IZKF2020\BG20_purified_Fr.38-42\0_H2\1 printed: 21.04.2020 14:18:05

449 **IZKF Leipzig, Core Unit Peptid-Technologien**
Liebigstraße 21, 04103 Leipzig, e-mail: sven_r@yahoo.com, Tel.: 0341 - 9715898 / 897

Comment: Fr.50-58

Sample Name:	**CK20_purified**	*Injection Volume:*	**15.0**
Vial Number:	**BC2**	*Channel:*	**UV_VIS_1**
Sample Type:	**unknown**	*Wavelength:*	**220.0**
Control Program:	**Peptide_3D_basic_short**	*Bandwidth:*	**4**
Quantif. Method:	**peptide_izkf**	*Dilution Factor:*	**1.0000**
Recording Time:	**7/7/2020 13:58**	*Operator:*	**KEYUSER**
Sample ID:		*Sample Amount:*	**1.0000**

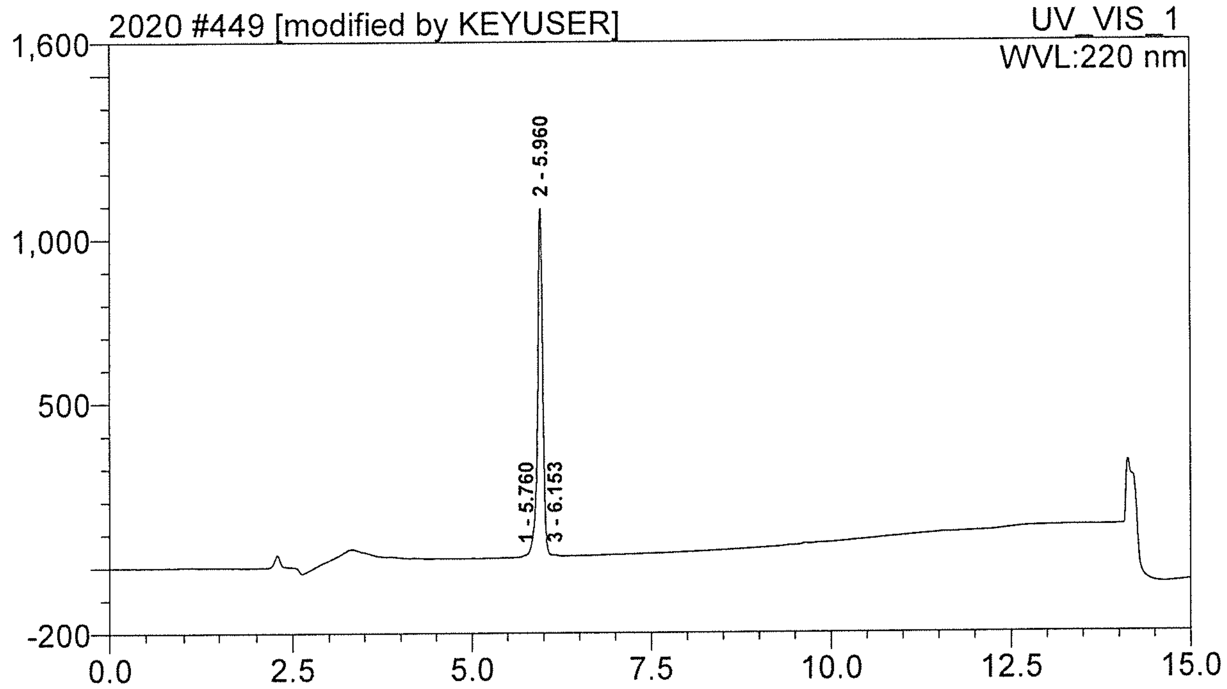

No.	Ret.Time min	Peak Name	Height mAU	Rel.Area %	Area mAU*min	Amount	Type
1	5.76	n.a.	7.2	0.50	0.44	n.a.	BM
2	5.96	n.a.	1059.6	99.05	87.42	n.a.	M
3	6.15	n.a.	5.4	0.44	0.39	n.a.	MB
Total:			1072.095	100.000	88.25	0.000	

Dr. Sven Rothemund, IZKF Leipzig, Liebigstrasse 21, 04103 Leipzig, Tel.: 0341-9715898, sven_r@yahoo.com

Comment 1 CK20_purified_Fr.50-58
Comment 2 MW:4380,2(M+H)+

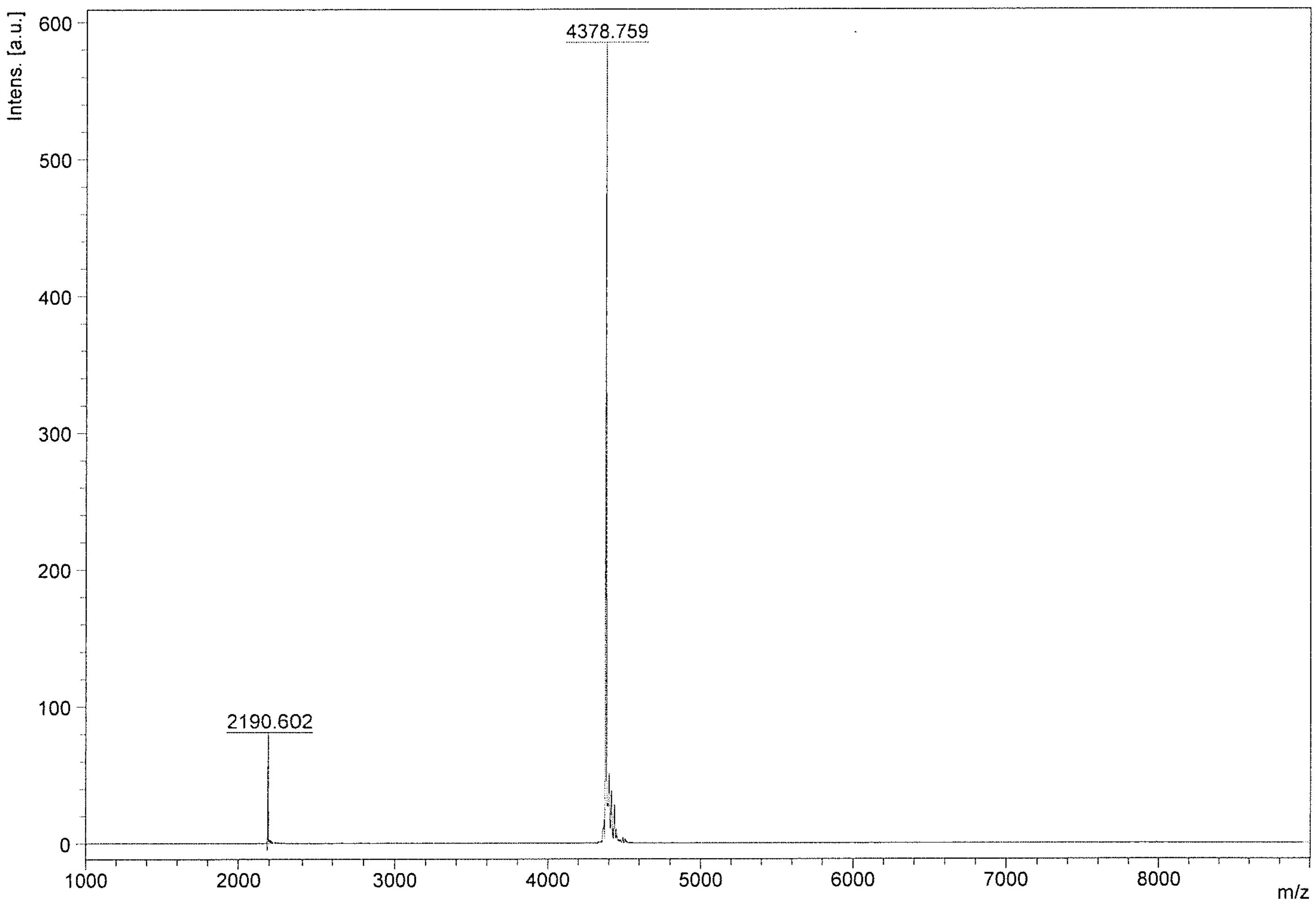

Acquisition Parameter

Date of acquisition	2020-07-07T14:06:17.646+02:00
Acquisition method name	D:\Methods\flexControlMethods\LP_PepMix.par
Aquisition operation mode	Linear
Voltage polarity	POS
Number of shots	46
Name of spectrum used for calibration	
Calibration reference list used	PeptideCalibStandardInsulin2 monoAv

Instrument Info

User	IZKF
Instrument	FLEX-PC

405 **IZKF Leipzig, Core Unit Peptid-Technologien**

Liebigstraße 21, 04103 Leipzig, e-mail: sven_r@yahoo.com, Tel.: 0341 - 9715898 / 897

Comment: Vial8-12

Sample Name:	**CC20_purified**	*Injection Volume:*	**20.0**
Vial Number:	**GE5**	*Channel:*	**UV_VIS_1**
Sample Type:	**unknown**	*Wavelength:*	**220.0**
Control Program:	**Peptide_3D_basic_short**	*Bandwidth:*	**4**
Quantif. Method:	**Peptide_3D_basic**	*Dilution Factor:*	**1.0000**
Recording Time:	**19/6/2020 11:02**	*Operator:*	**KEYUSER**
Sample ID:		*Sample Amount:*	**1.0000**

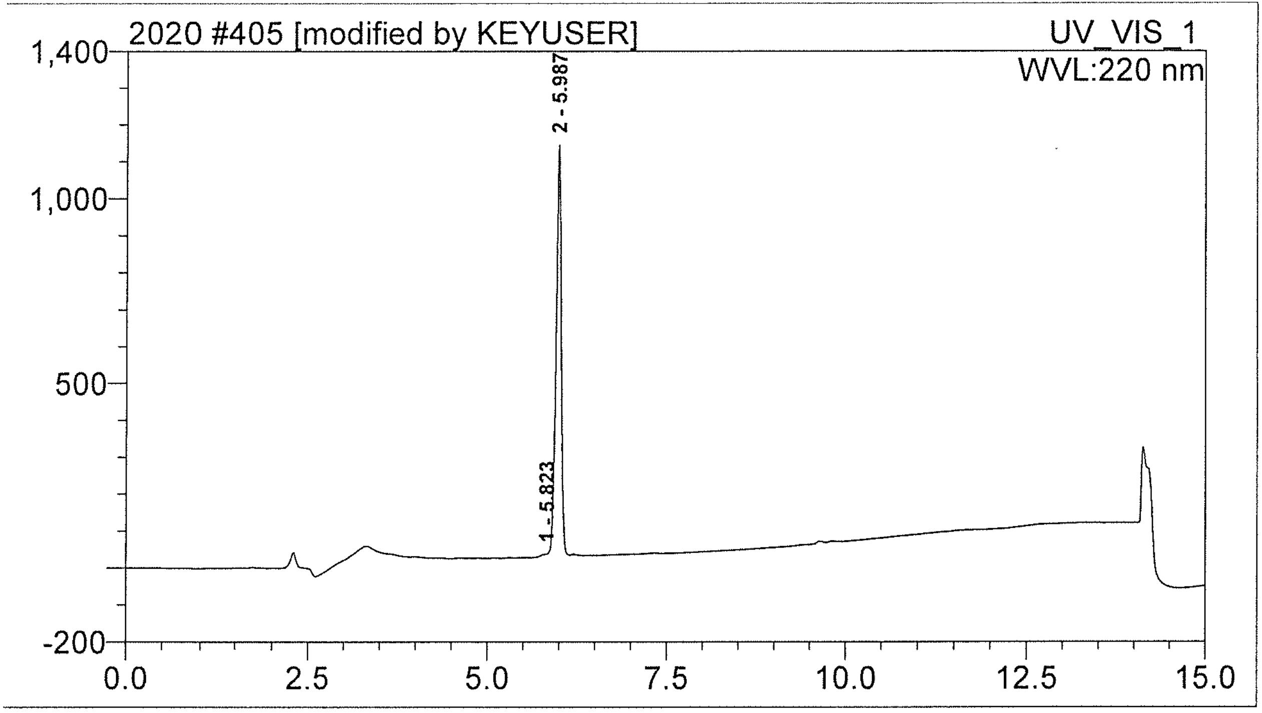

No.	Ret.Time min	Peak Name	Height mAU	Rel.Area %	Area mAU*min	Amount	Type
1	5.82	n.a.	5.5	0.41	0.38	n.a.	BM *
2	5.99	n.a.	1111.5	99.59	92.24	n.a.	MB*
**Total:**			1116.958	100.000	92.62	0.000	

Dr. Sven Rothemund, IZKF Leipzig, Liebigstrasse 21, 04103 Leipzig, Tel.: 0341-9715898, sven_r@yahoo.com

Comment 1 CC20_purified
Comment 2 MW:av.4356,46(M+H)+

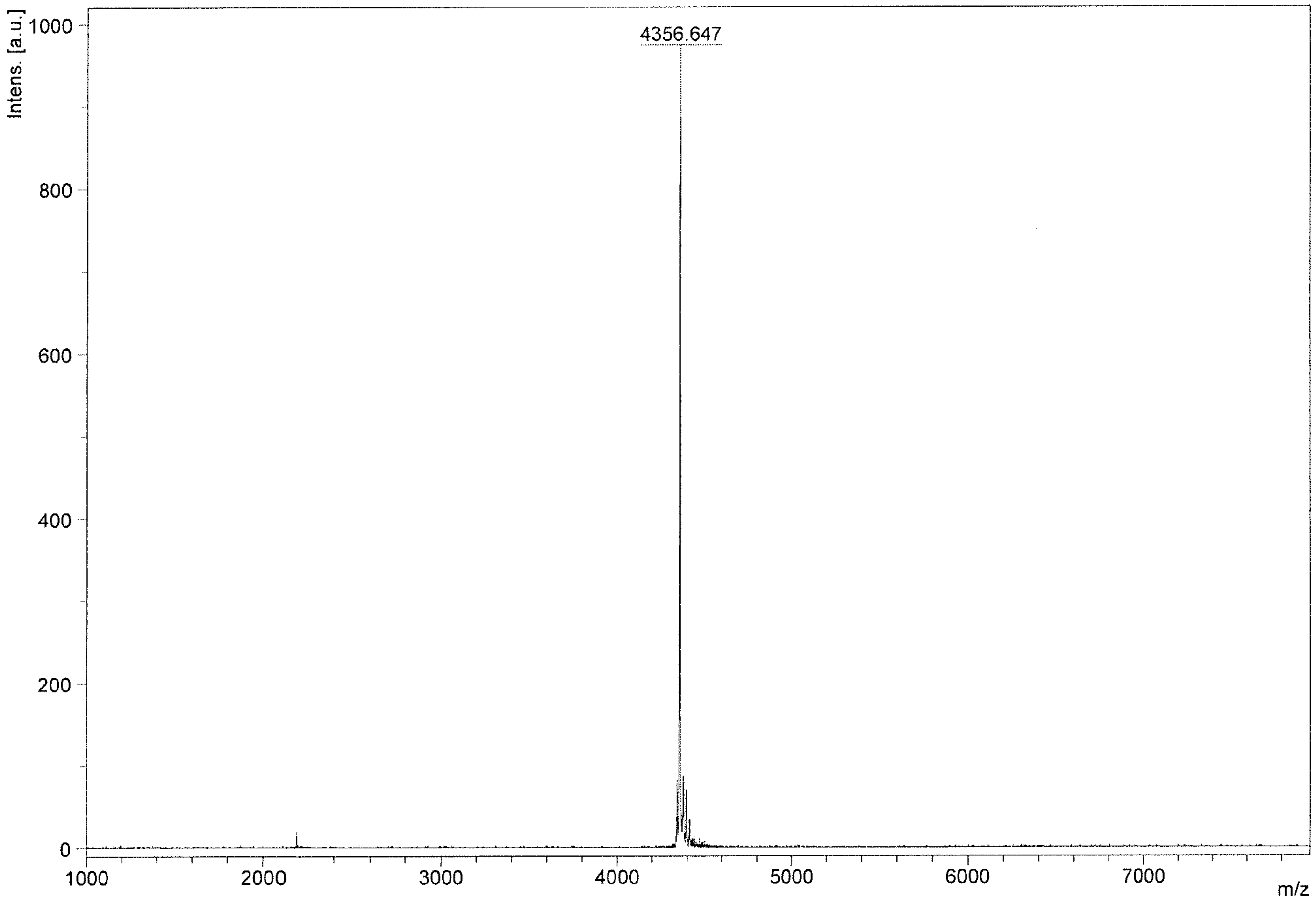

Acquisition Parameter

Date of acquisition	2020-06-19T11:13:25.005+02:00
Acquisition method name	D:\Methods\flexControlMethods\LP_PepMix.par
Aquisition operation mode	Linear
Voltage polarity	POS
Number of shots	100
Name of spectrum used for calibration	
Calibration reference list used	PeptideCalibStandardInsulin2 monoAv

Instrument Info

User	IZKF
Instrument	FLEX-PC

D:\Data\IZKF2020\CC20_purified\0_E10\1 printed: 19.06.2020 11:14:00

591 IZKF Leipzig, Core Unit Peptid-Technologien
Liebigstraße 21, 04103 Leipzig, e-mail: sven_r@yahoo.com, Tel.: 0341 - 9715898 / 897

Comment:

Sample Name:	**DL20_purified**	*Injection Volume:*	**20.0**
Vial Number:	**BB4**	*Channel:*	**UV_VIS_1**
Sample Type:	**unknown**	*Wavelength:*	**220.0**
Control Program:	**Peptide_3D_basic_short**	*Bandwidth:*	**4**
Quantif. Method:	**peptide_izkf**	*Dilution Factor:*	**1.0000**
Recording Time:	**15/9/2020 12:26**	*Operator:*	**KEYUSER**
Sample ID:		*Sample Amount:*	**1.0000**

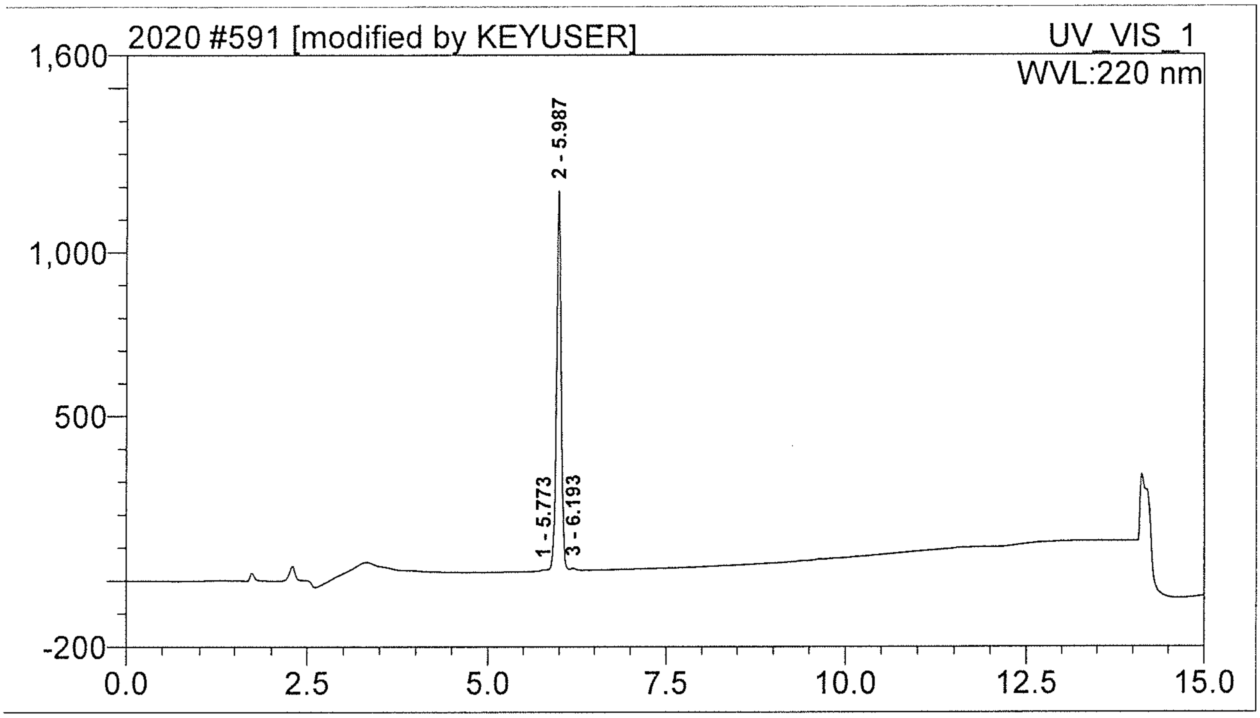

No.	Ret.Time min	Peak Name	Height mAU	Rel.Area %	Area mAU*min	Amount	Type
1	5.77	n.a.	4.5	0.41	0.36	n.a.	BM
2	5.99	n.a.	1157.3	98.90	87.71	n.a.	M
3	6.19	n.a.	7.5	0.69	0.61	n.a.	MB
**Total:**			1169.281	100.000	88.68	0.000	

Chromeleon (c) Dionex 1996-2006
Version 6.80 SR15 Build 4656 (243203)

Dr. Sven Rothemund, IZKF Leipzig, Liebigstrasse 21, 04103 Leipzig, Tel.: 0341-9715898, sven_r@yahoo.com

Comment 1	DL20_purified
Comment 2	MW: 4379,4(M+H)+

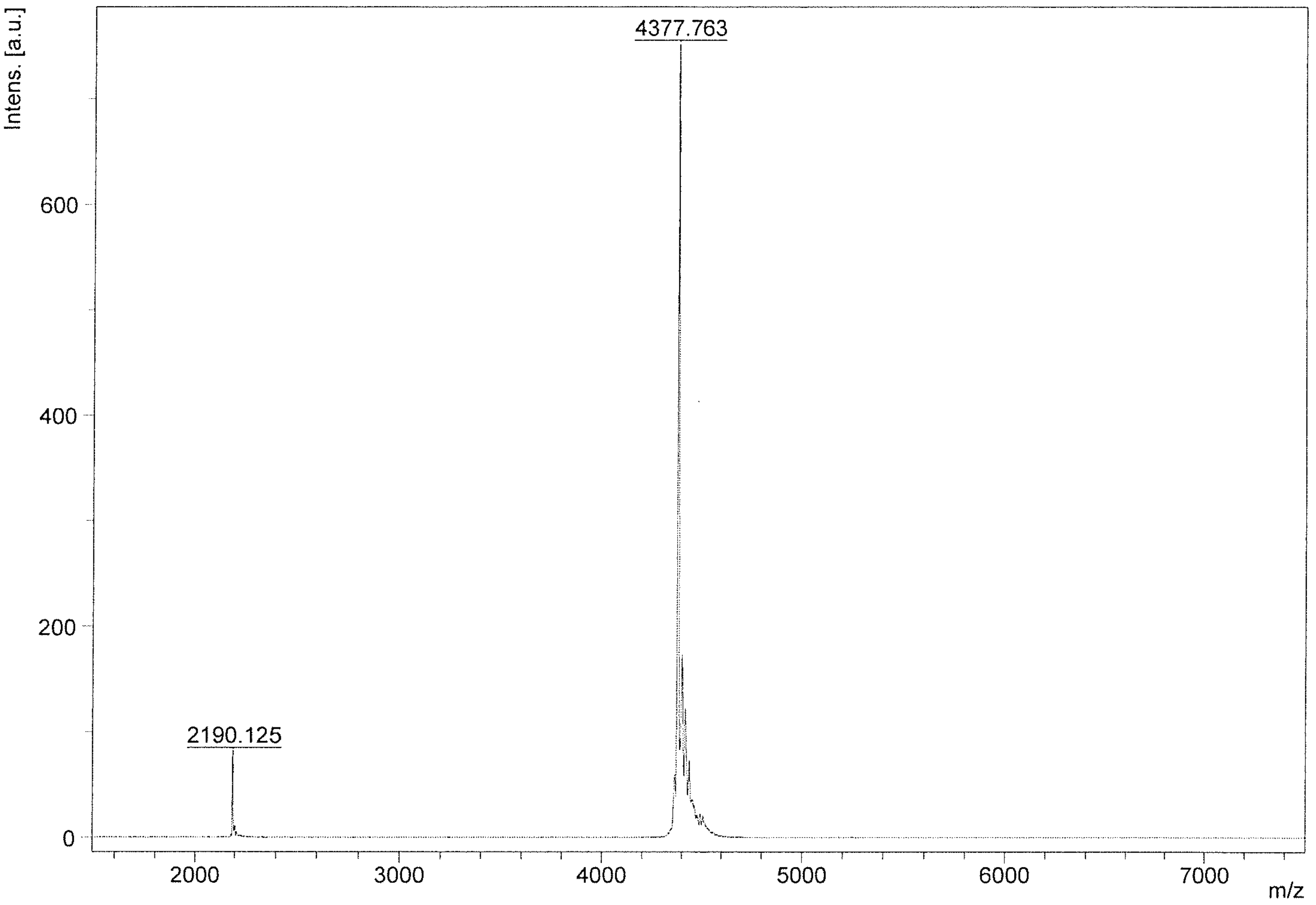

Acquisition Parameter

Date of acquisition	2020-09-15T12:30:43.954+02:00
Acquisition method name	D:\Methods\flexControlMethods\LP_PepMix.par
Aquisition operation mode	Linear
Voltage polarity	POS
Number of shots	34
Name of spectrum used for calibration	
Calibration reference list used	PeptideCalibStandardInsulin2 monoAv

Instrument Info

User	IZKF
Instrument	FLEX-PC

D:\Data\IZKF2020\DL20_purified\0_B10\1 printed: 15.09.2020 12:33:31

Dr. Sven Rothemund, IZKF Leipzig, Liebigstrasse 21, 04103 Leipzig, Tel.: 0341-9715898, sven_r@yahoo.com

Comment 1 DL20_purified
Comment 2 MW: 4379,4(M+H)+

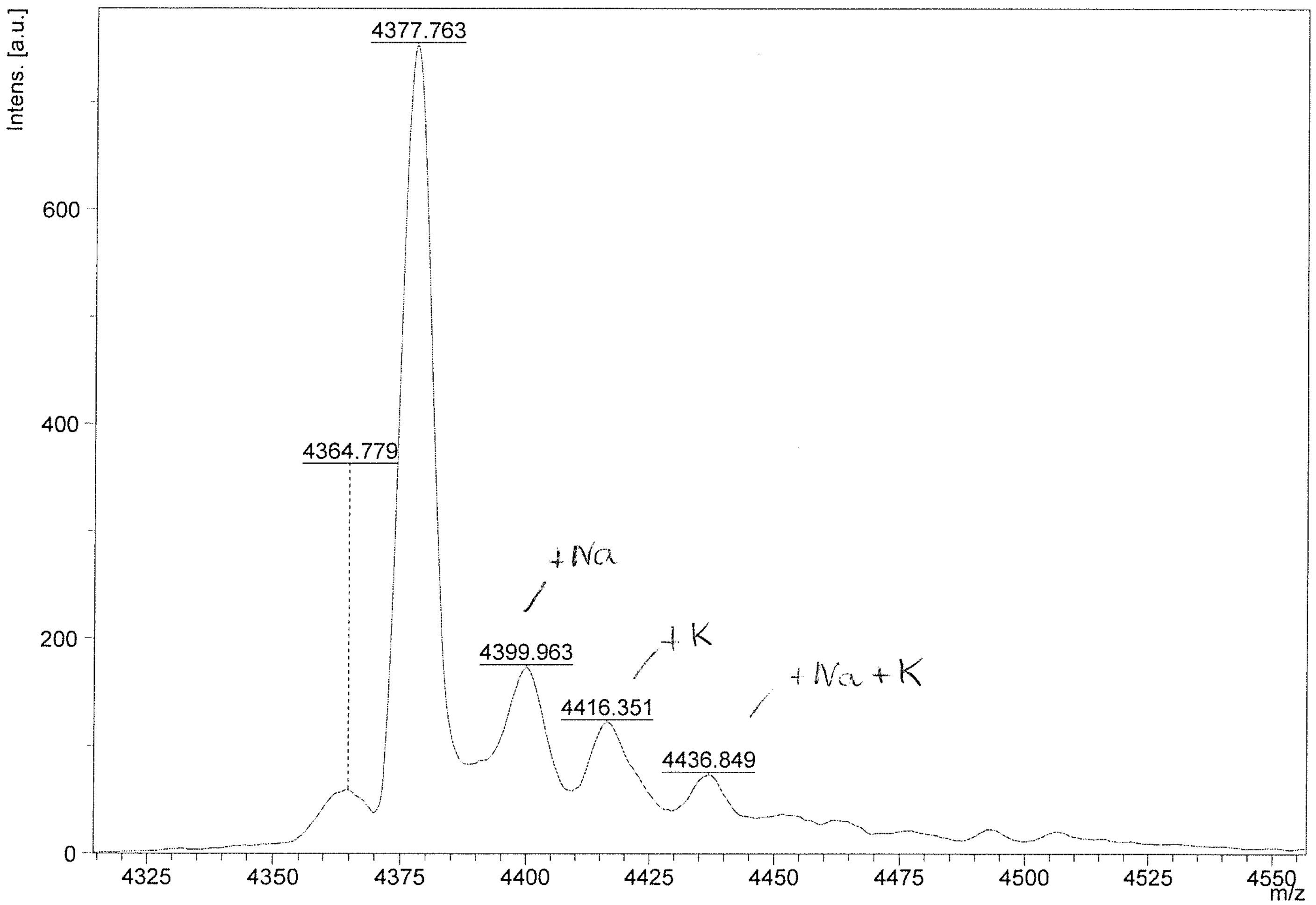

Acquisition Parameter

Date of acquisition	2020-09-15T12:30:43.954+02:00
Acquisition method name	D:\Methods\flexControlMethods\LP_PepMix.par
Aquisition operation mode	Linear
Voltage polarity	POS
Number of shots	34
Name of spectrum used for calibration	
Calibration reference list used	PeptideCalibStandardInsulin2 monoAv

Instrument Info

User	IZKF
Instrument	FLEX-PC

D:\Data\IZKF2020\DL20_purified\0_B10\1 printed: 15.09.2020 12:32:54

421 **IZKF Leipzig, Core Unit Peptid-Technologien**
Liebigstraße 21, 04103 Leipzig, e-mail: sven_r@yahoo.com, Tel.: 0341 - 9715898 / 897

Comment: Fr.37-46

Sample Name:	**CD20_purified2**	*Injection Volume:*	**20.0**
Vial Number:	**RC3**	*Channel:*	**UV_VIS_1**
Sample Type:	**unknown**	*Wavelength:*	**220.0**
Control Program:	**Peptide_3D_basic_short**	*Bandwidth:*	**4**
Quantif. Method:	**peptide_izkf**	*Dilution Factor:*	**1.0000**
Recording Time:	**24/6/2020 14:55**	*Operator:*	**KEYUSER**
Sample ID:		*Sample Amount:*	**1.0000**

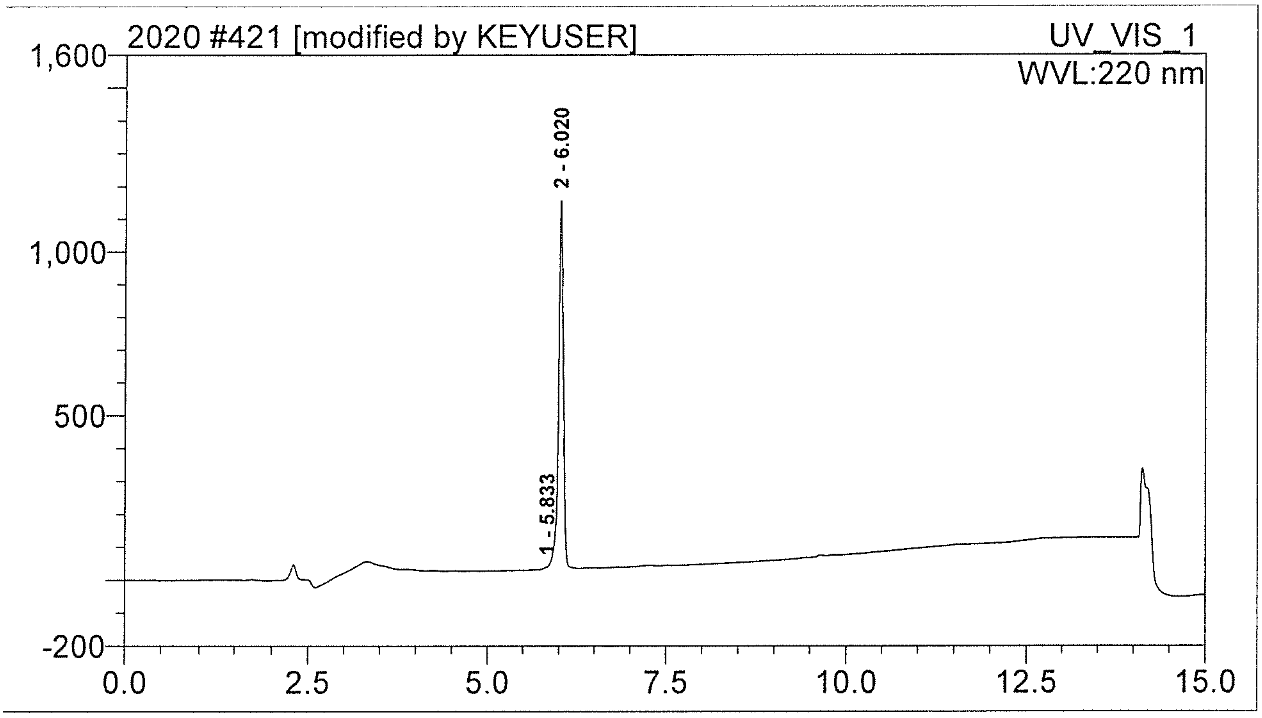

No.	Ret.Time min	Peak Name	Height mAU	Rel.Area %	Area mAU*min	Amount	Type
1	5.83	n.a.	7.8	0.51	0.45	n.a.	BM *
2	6.02	n.a.	1123.1	99.49	87.95	n.a.	MB*
**Total:**			1130.983	100.000	88.40	0.000	

Chromeleon (c) Dionex 1996-2006
Version 6.80 SR15 Build 4656 (243203)

Dr. Sven Rothemund, IZKF Leipzig, Liebigstrasse 21, 04103 Leipzig, Tel.: 0341-9715898, sven_r@yahoo.com

Comment 1 CD20_purified2_Fr.37-46
Comment 2 MW:4384,5(M+H)+

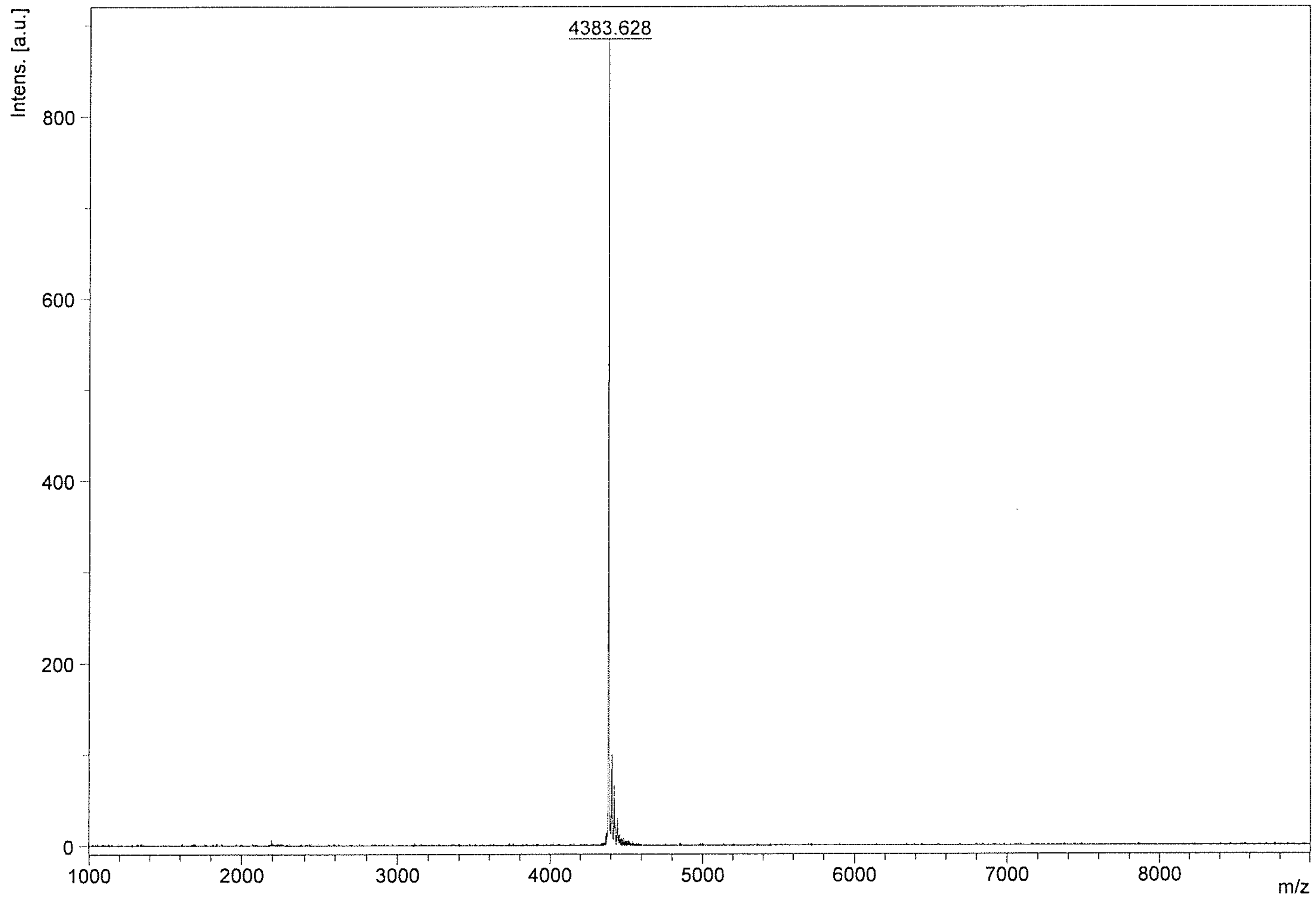

Acquisition Parameter

Date of acquisition	2020-06-24T15:07:30.214+02:00
Acquisition method name	D:\Methods\flexControlMethods\LP_PepMix.par
Aquisition operation mode	Linear
Voltage polarity	POS
Number of shots	82
Name of spectrum used for calibration	
Calibration reference list used	PeptideCalibStandardInsulin2 monoAv

Instrument Info

User	IZKF
Instrument	FLEX-PC

D:\Data\IZKF2020\CD20_purified2_Fr.37-46\0_B9\1 printed: 24.06.2020 15:07:57

604 **IZKF Leipzig, Core Unit Peptid-Technologien**
Liebigstraße 21, 04103 Leipzig, e-mail: sven_r@yahoo.com, Tel.: 0341 - 9715898 / 897

Comment:

Sample Name:	**DM20-purified**	*Injection Volume:*	**20.0**
Vial Number:	**GE2**	*Channel:*	**UV_VIS_1**
Sample Type:	**unknown**	*Wavelength:*	**220.0**
Control Program:	**Peptide_3D_basic_short**	*Bandwidth:*	**4**
Quantif. Method:	**peptide_izkf**	*Dilution Factor:*	**1.0000**
Recording Time:	**18/9/2020 14:21**	*Operator:*	**KEYUSER**
Sample ID:		*Sample Amount:*	**1.0000**

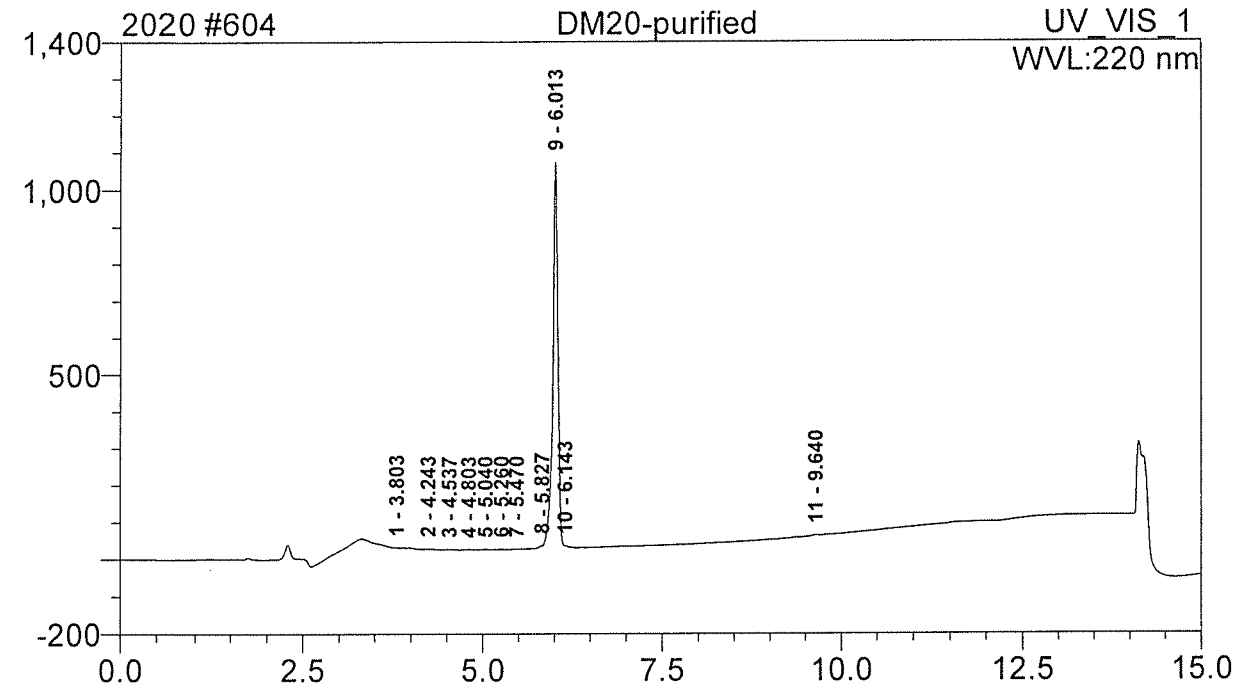

No.	Ret.Time min	Peak Name	Height mAU	Rel.Area %	Area mAU*min	Amount	Type
1	3.80	n.a.	0.0	0.32	0.28	n.a.	BMB
2	4.24	n.a.	0.9	0.15	0.13	n.a.	BMB
3	4.54	n.a.	0.9	0.13	0.12	n.a.	BMB
4	4.80	n.a.	0.9	0.12	0.11	n.a.	BM
5	5.04	n.a.	0.7	0.08	0.07	n.a.	M
6	5.26	n.a.	1.1	0.14	0.12	n.a.	MB
7	5.47	n.a.	0.7	0.07	0.06	n.a.	BMB
8	5.83	n.a.	7.2	0.41	0.37	n.a.	BM
9	6.01	n.a.	1043.6	97.91	86.63	n.a.	M
10	6.14	n.a.	5.8	0.43	0.38	n.a.	MB
11	9.64	n.a.	2.2	0.23	0.21	n.a.	BMB
**Total:**			1063.944	100.000	88.48	0.000	

Dr. Sven Rothemund, IZKF Leipzig, Liebigstrasse 21, 04103 Leipzig, Tel.: 0341-9715898, sven_r@yahoo.com

Comment 1	DM20_purified
Comment 2	MW:4407,5(M+H)+

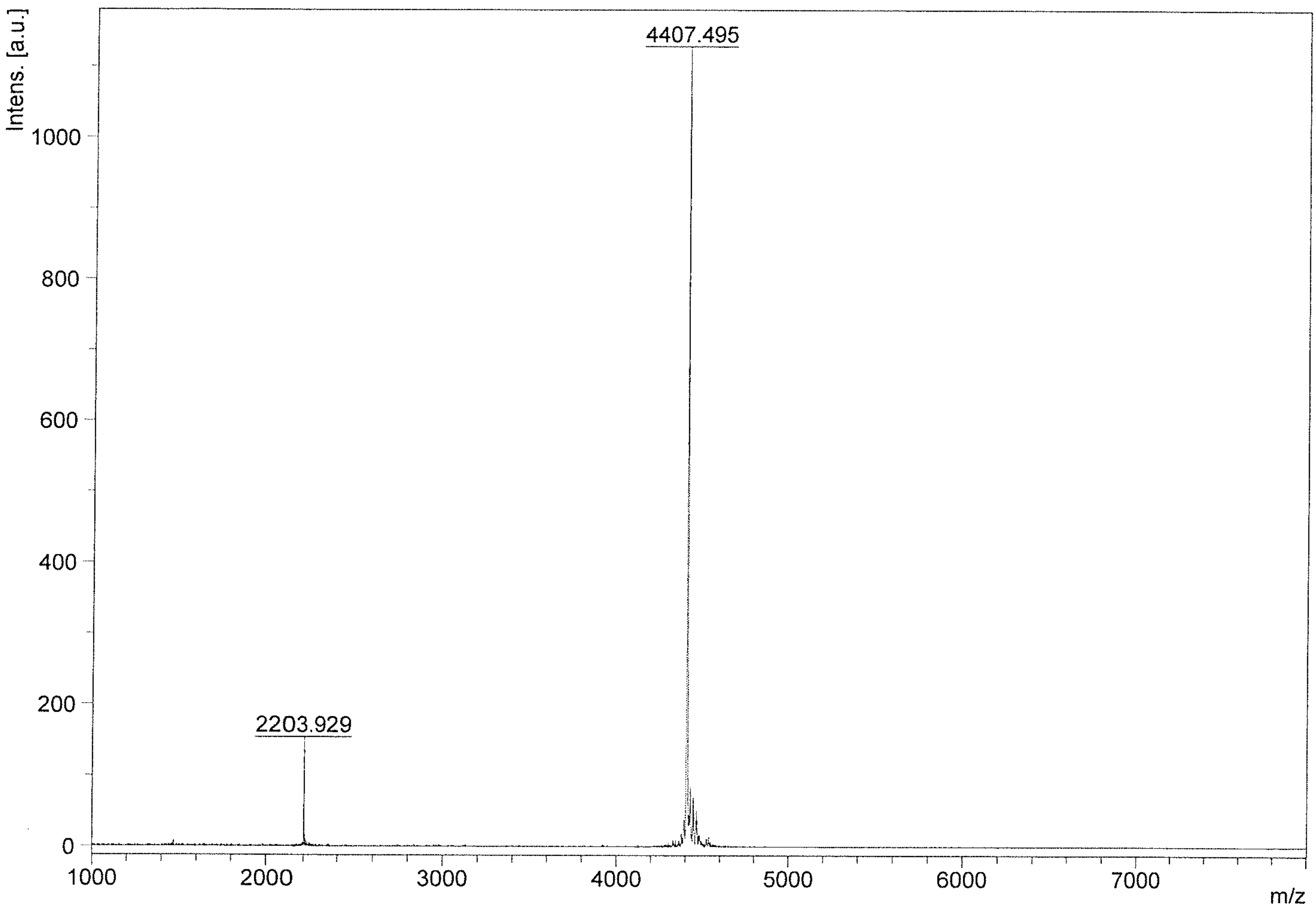

Acquisition Parameter

Date of acquisition	2020-09-18T14:35:37.520+02:00
Acquisition method name	D:\Methods\flexControlMethods\LP_PepMix.par
Aquisition operation mode	Linear
Voltage polarity	POS
Number of shots	78
Name of spectrum used for calibration	
Calibration reference list used	PeptideCalibStandardInsulin2 monoAv

Instrument Info

User	IZKF
Instrument	FLEX-PC

D:\Data\IZKF2020\DM20_purified\0_F8\1 printed: 18.09.2020 14:36:34

Dr. Sven Rothemund, IZKF Leipzig, Liebigstrasse 21, 04103 Leipzig, Tel.: 0341-9715898, sven_r@yahoo.com

Comment 1	DM20_purified
Comment 2	MW:4407,5(M+H)+

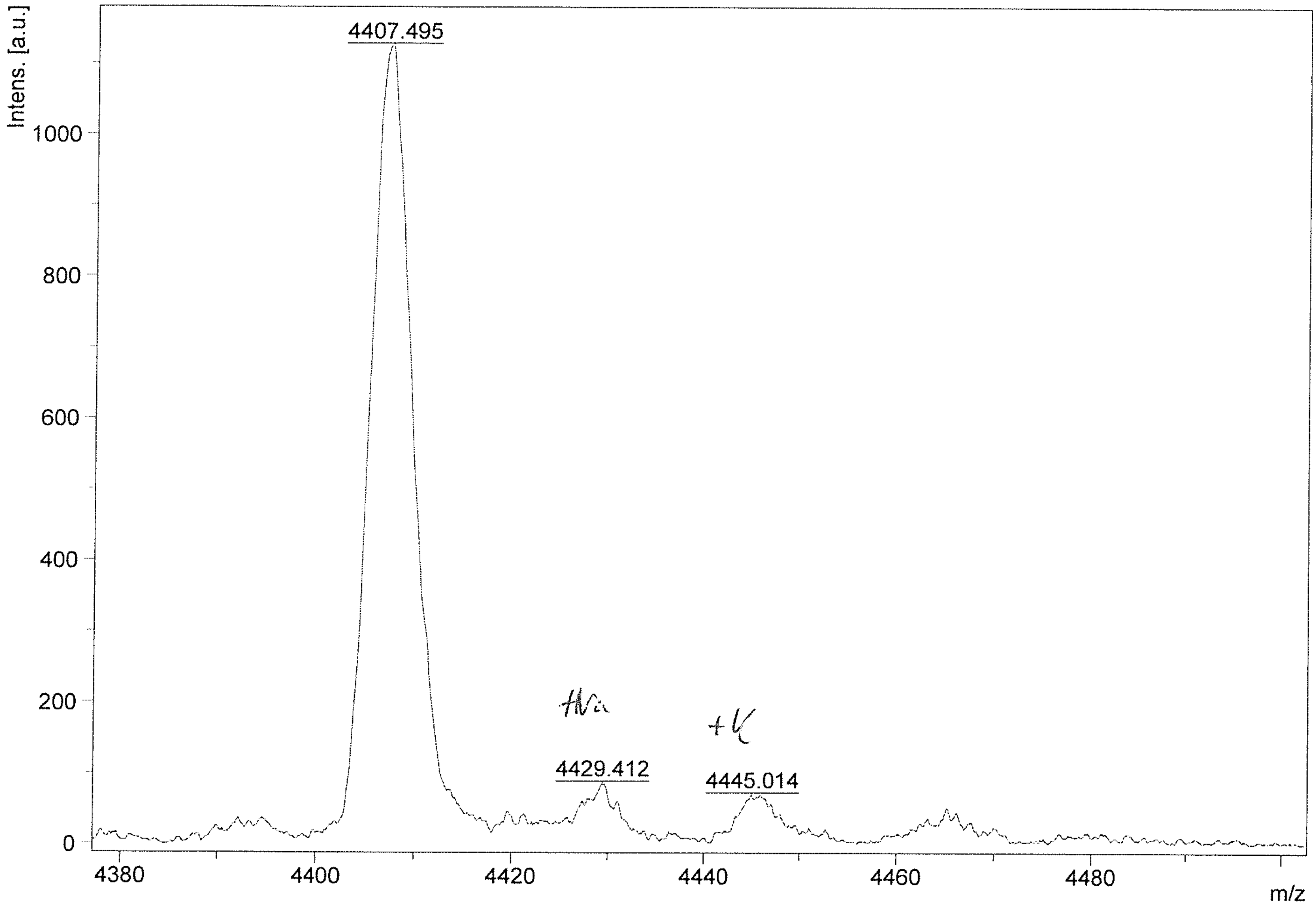

Acquisition Parameter

Date of acquisition	2020-09-18T14:35:37.520+02:00
Acquisition method name	D:\Methods\flexControlMethods\LP_PepMix.par
Aquisition operation mode	Linear
Voltage polarity	POS
Number of shots	78
Name of spectrum used for calibration	
Calibration reference list used	PeptideCalibStandardInsulin2 monoAv

Instrument Info

User	IZKF
Instrument	FLEX-PC

197 IZKF Leipzig, Core Unit Peptid-Technologien
Liebigstraße 21, 04103 Leipzig, e-mail: sven_r@yahoo.com, Tel.: 0341 - 9715898 / 897

Comment:

Sample Name:	**A20 purif**	*Injection Volume:*	**20.0**
Vial Number:	**BE7**	*Channel:*	**UV_VIS_1**
Sample Type:	**unknown**	*Wavelength:*	**220.0**
Control Program:	**Peptide_3D_basic_short**	*Bandwidth:*	**4**
Quantif. Method:	**peptide_izkf**	*Dilution Factor:*	**1.0000**
Recording Time:	**19/2/2020 11:58**	*Operator:*	**KEYUSER**
Sample ID:		*Sample Amount:*	**1.0000**

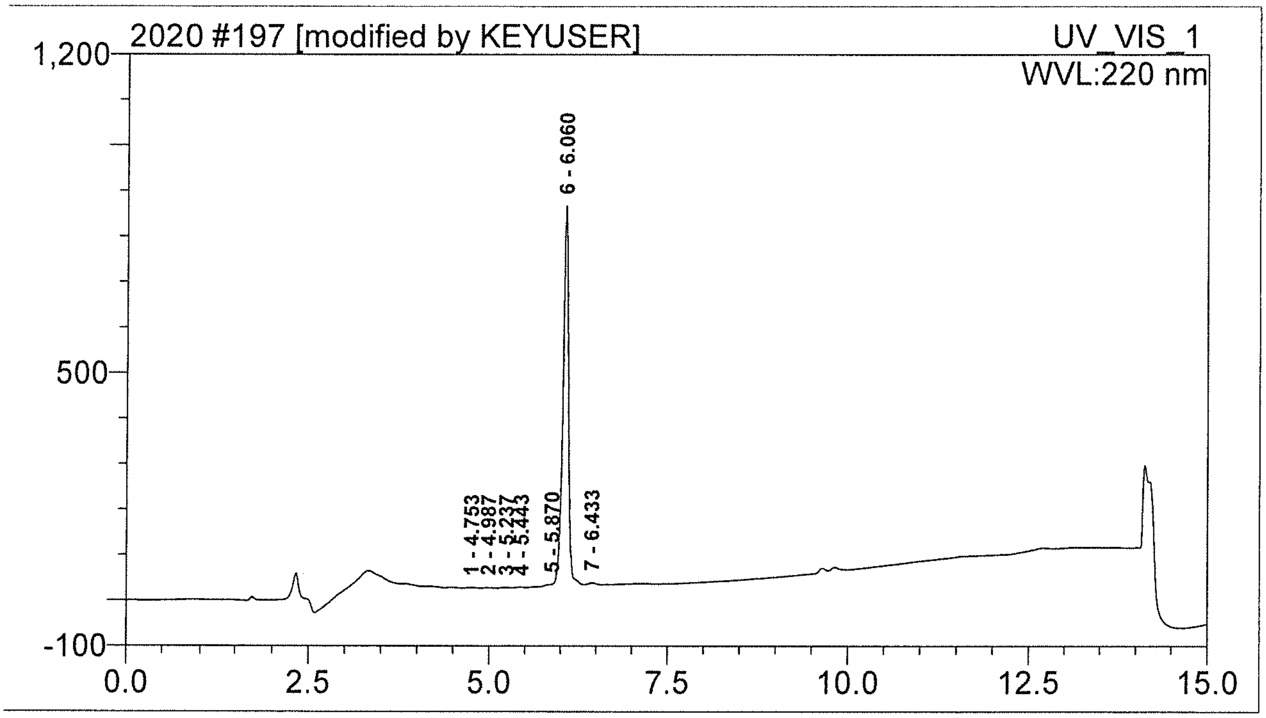

No.	Ret.Time min	Peak Name	Height mAU	Rel.Area %	Area mAU*min	Amount	Type
1	4.75	n.a.	1.0	0.16	0.13	n.a.	BMB
2	4.99	n.a.	0.9	0.12	0.09	n.a.	BMB
3	5.24	n.a.	0.9	0.13	0.10	n.a.	BM
4	5.44	n.a.	1.0	0.13	0.10	n.a.	MB
5	5.87	n.a.	4.1	0.53	0.41	n.a.	BM
6	6.06	n.a.	835.7	98.07	76.23	n.a.	M
7	6.43	n.a.	5.7	0.84	0.66	n.a.	MB
**Total:**			849.231	100.000	77.73	0.000	

Chromeleon (c) Dionex 1996-2006
Version 6.80 SR15 Build 4656 (243203)

Dr. Sven Rothemund, IZKF Leipzig, Liebigstrasse 21, 04103 Leipzig, Tel.: 0341-9715898, sven_r@yahoo.com

Comment 1 A20 purified
Comment 2 MW:4366,2(M+H)+

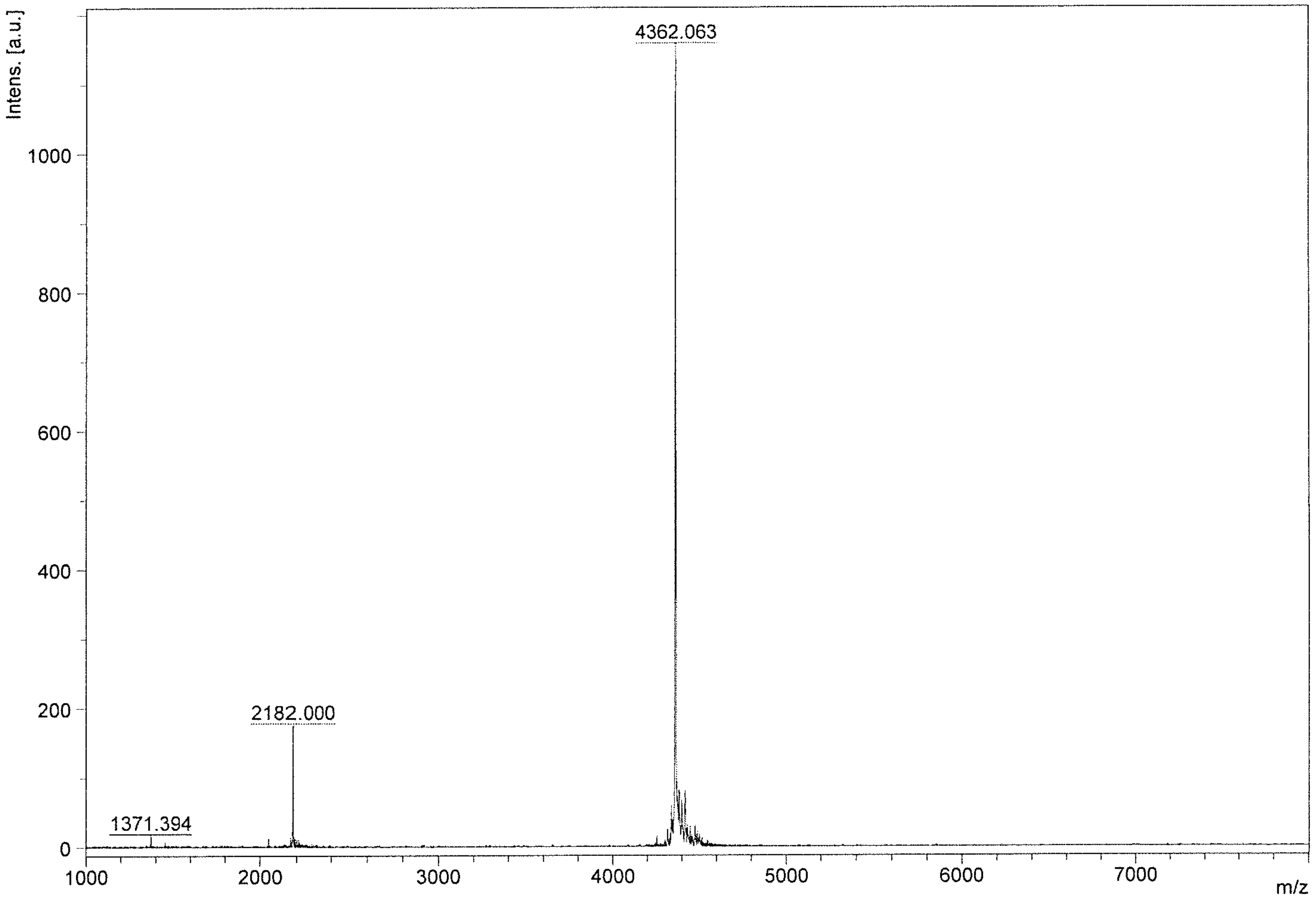

Acquisition Parameter

Date of acquisition	2020-02-19T12:18:47.759+01:00
Acquisition method name	D:\Methods\flexControlMethods\LP_PepMix.par
Aquisition operation mode	Linear
Voltage polarity	POS
Number of shots	100
Name of spectrum used for calibration	
Calibration reference list used	PeptideCalibStandardInsulin2 monoAv

Instrument Info

User	IZKF
Instrument	FLEX-PC

D:\Data\IZKF2020\A20 purified\0_A1\1 printed: 19.02.2020 12:29:49

206 IZKF Leipzig, Core Unit Peptid-Technologien
Liebigstraße 21, 04103 Leipzig, e-mail: sven_r@yahoo.com, Tel.: 0341 - 9715898 / 897

Comment: Fr.25-35

Sample Name:	**B20_purified**	*Injection Volume:*	**20.0**
Vial Number:	**GA2**	*Channel:*	**UV_VIS_1**
Sample Type:	**unknown**	*Wavelength:*	**220.0**
Control Program:	**Peptide_3D_basic_short**	*Bandwidth:*	**4**
Quantif. Method:	**peptide_izkf**	*Dilution Factor:*	**1.0000**
Recording Time:	**20/2/2020 10:56**	*Operator:*	**KEYUSER**
Sample ID:		*Sample Amount:*	**1.0000**

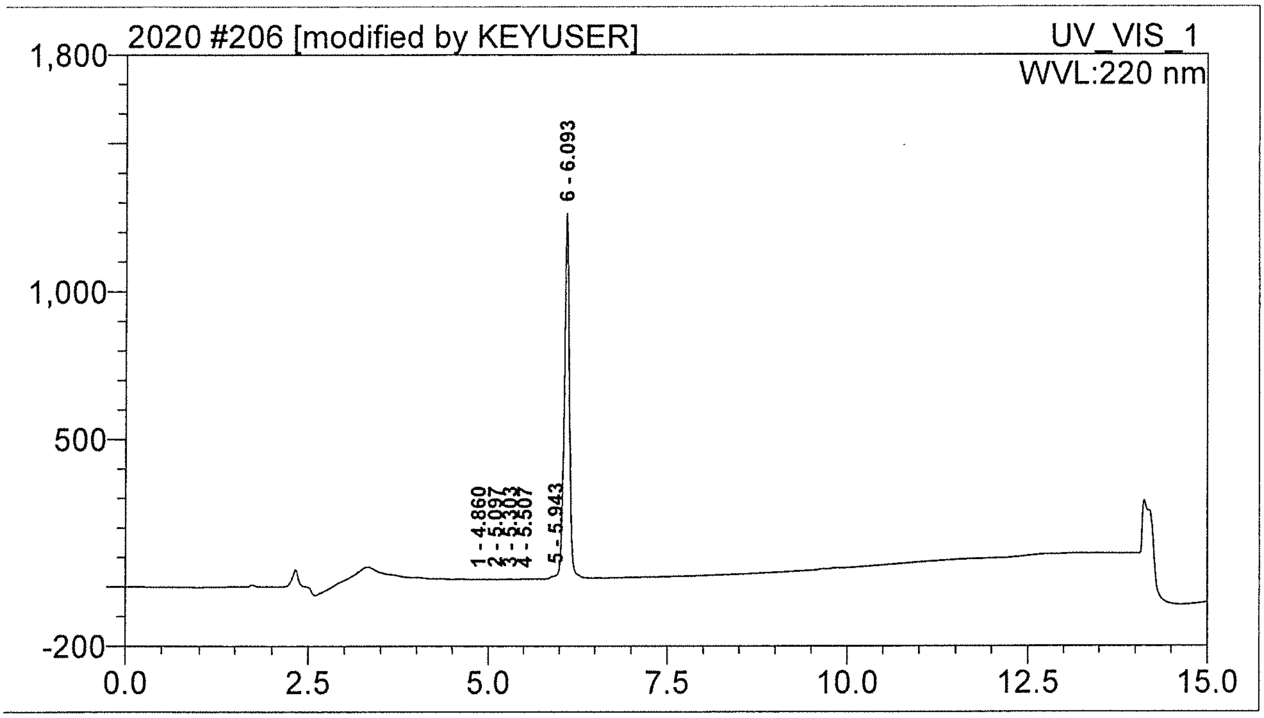

No.	Ret.Time min	Peak Name	Height mAU	Rel.Area %	Area mAU*min	Amount	Type
1	4.86	n.a.	1.0	0.11	0.12	n.a.	BMB
2	5.10	n.a.	0.8	0.08	0.09	n.a.	BMB
3	5.30	n.a.	0.9	0.09	0.09	n.a.	BMB
4	5.51	n.a.	0.6	0.06	0.06	n.a.	BMB
5	5.94	n.a.	10.3	0.65	0.69	n.a.	BM
6	6.09	n.a.	1235.3	99.01	104.76	n.a.	MB
Total:			1248.870	100.000	105.81	0.000	

Dr. Sven Rothemund, IZKF Leipzig, Liebigstrasse 21, 04103 Leipzig, Tel.: 0341-9715898, sven_r@yahoo.com

Comment 1	B20_purified
Comment 2	MWav4379,5.(M+H)+

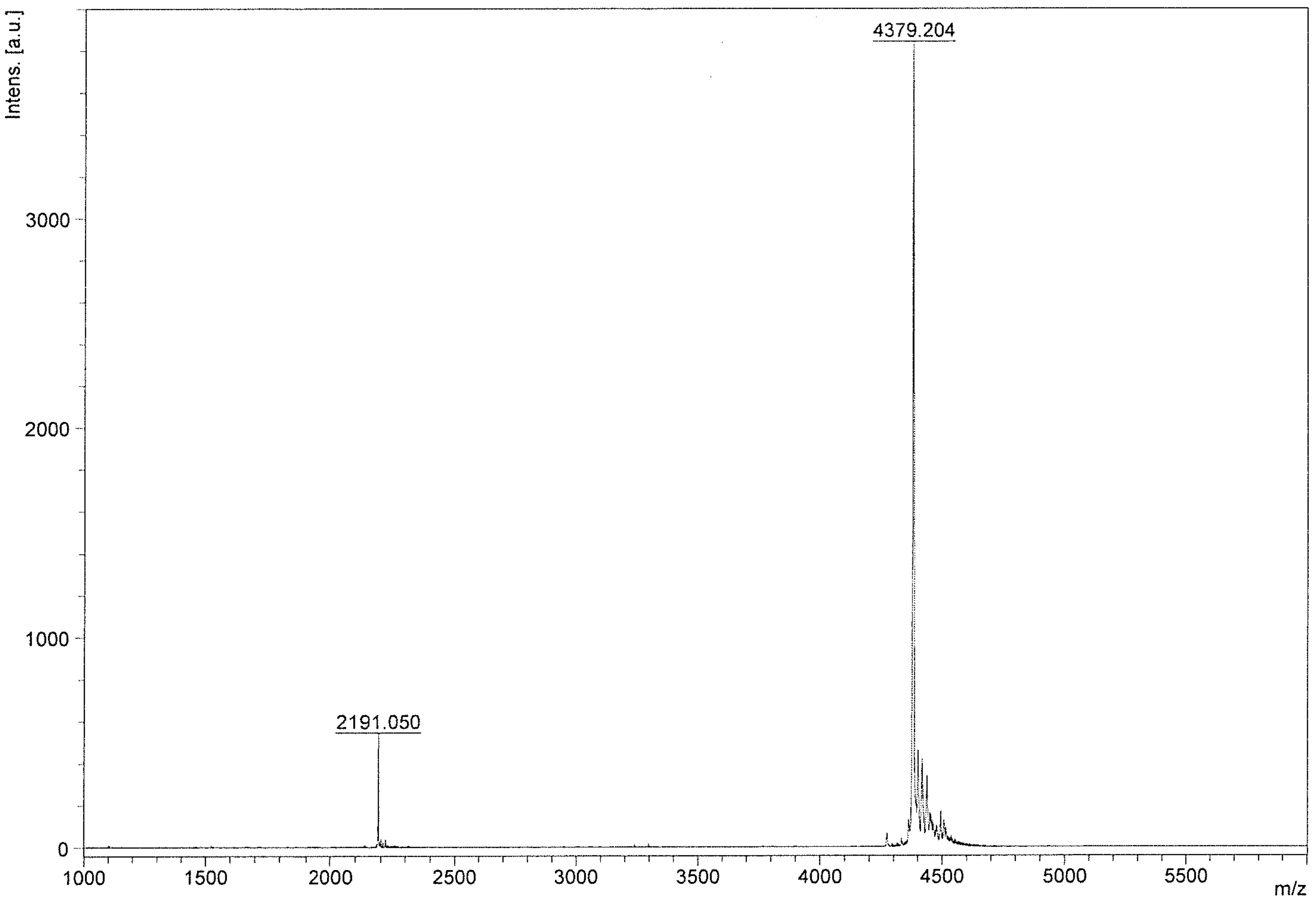

Acquisition Parameter

Date of acquisition	2020-02-20T10:40:22.000
Acquisition method name	D:\Methods\flexControlMethods\LP_PepMix.par
Aquisition operation mode	Linear
Voltage polarity	POS
Number of shots	100
Name of spectrum used for calibration	
Calibration reference list used	PeptideCalibStandardInsulin2 monoAv

Instrument Info

User	IZKF
Instrument	FLEX-PC

D:\Data\IZKF2020\B20_purified\0_H1\1 printed: 20.02.2020 11:42:09

398 **IZKF Leipzig, Core Unit Peptid-Technologien**

Liebigstraße 21, 04103 Leipzig, e-mail: sven_r@yahoo.com, Tel.: 0341 - 9715898 / 897

Comment: Fr.10-16

Sample Name:	**CB20_purified**	*Injection Volume:*	**20.0**
Vial Number:	**GB3**	*Channel:*	**UV_VIS_1**
Sample Type:	**unknown**	*Wavelength:*	**220.0**
Control Program:	**Peptide_3D_basic_short**	*Bandwidth:*	**4**
Quantif. Method:	**peptide_izkf**	*Dilution Factor:*	**1.0000**
Recording Time:	**16/6/2020 12:19**	*Operator:*	**KEYUSER**
Sample ID:		*Sample Amount:*	**1.0000**

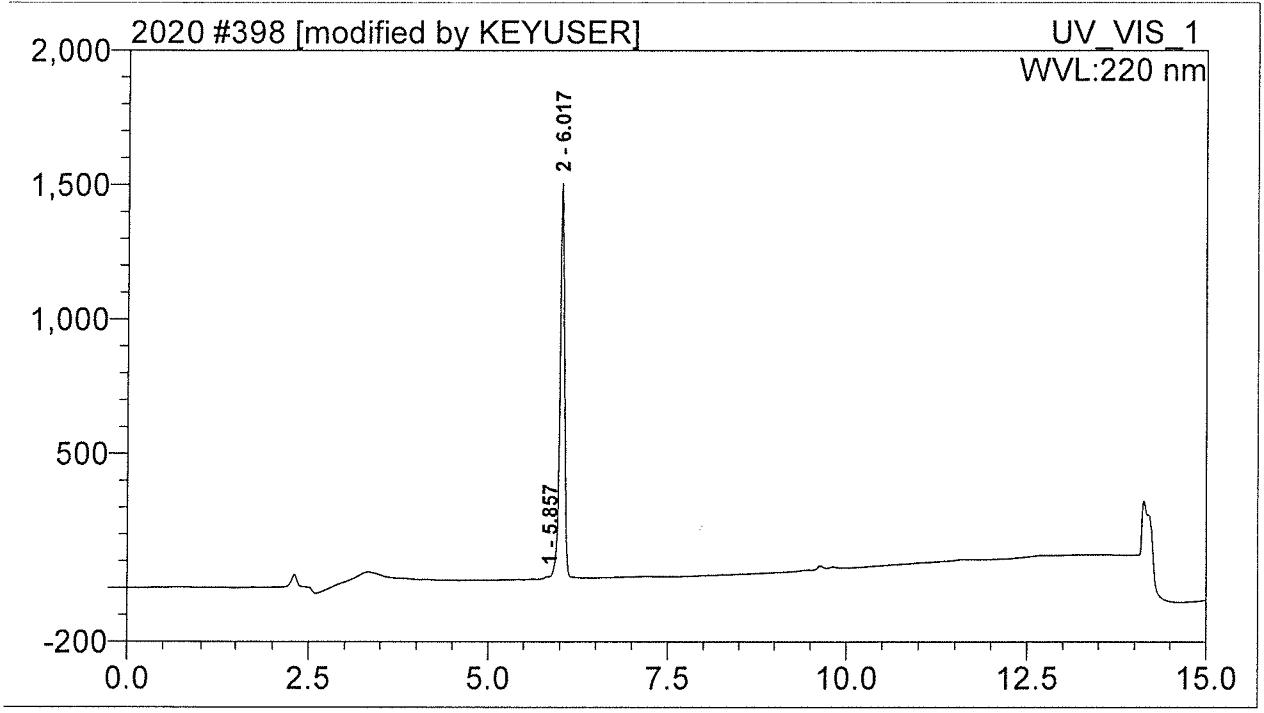

No.	Ret.Time min	Peak Name	Height mAU	Rel.Area %	Area mAU*min	Amount	Type
1	5.86	n.a.	8.6	0.57	0.63	n.a.	BM *
2	6.02	n.a.	1468.7	99.43	110.00	n.a.	MB*
**Total:**			1477.307	100.000	110.64	0.000	

Chromeleon (c) Dionex 1996-2006
Version 6.80 SR15 Build 4656 (243203)

Dr. Sven Rothemund, IZKF Leipzig, Liebigstrasse 21, 04103 Leipzig, Tel.: 0341-9715898, sven_r@yahoo.com

Comment 1 CB20_purified_Fr.10-16
Comment 2 MW:av.4343,1(M+H)+

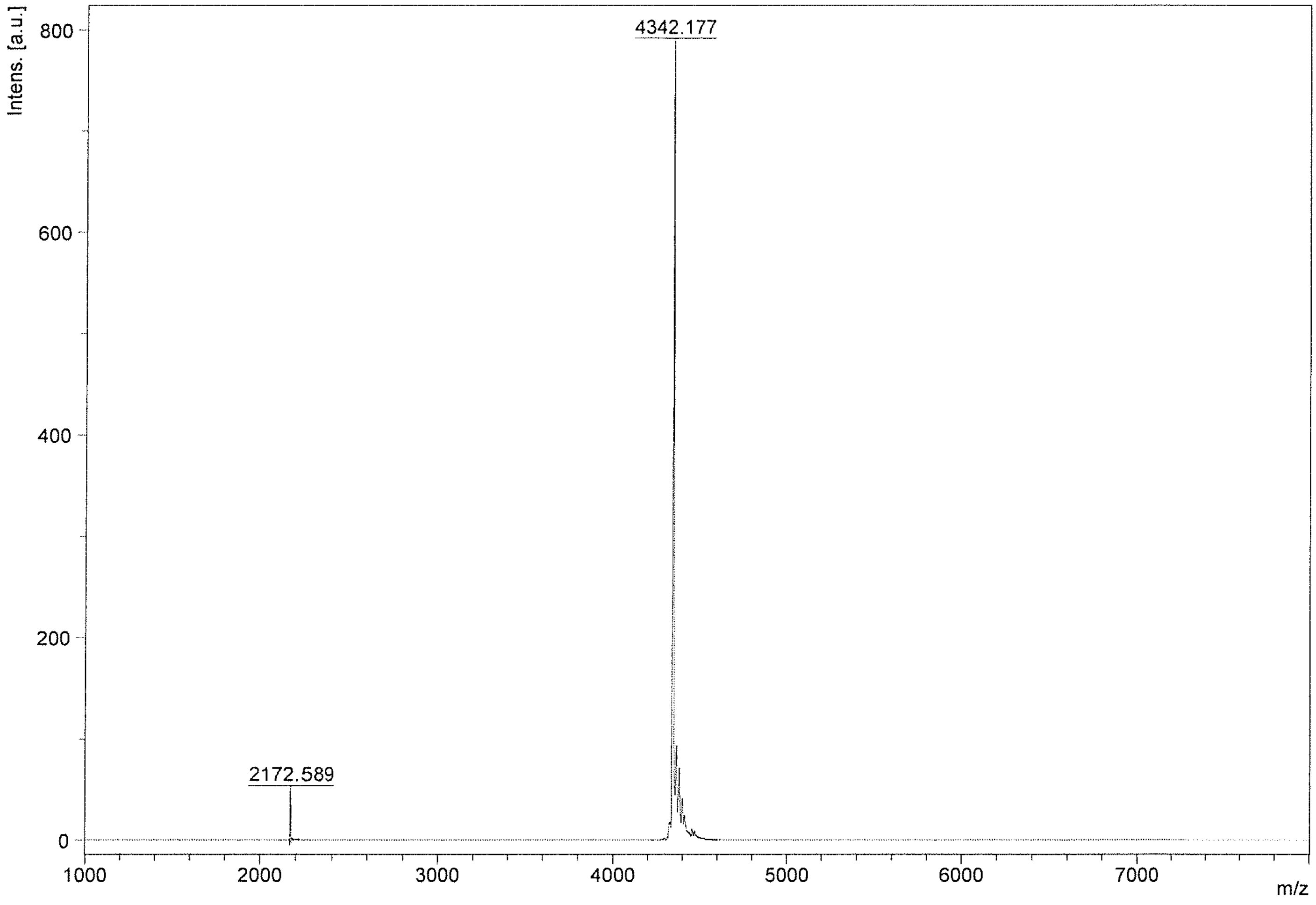

Acquisition Parameter

Date of acquisition	2020-06-16T12:49:15.719+02:00
Acquisition method name	D:\Methods\flexControlMethods\LP_PepMix.par
Aquisition operation mode	Linear
Voltage polarity	POS
Number of shots	86
Name of spectrum used for calibration	
Calibration reference list used	PeptideCalibStandardInsulin2 monoAv

Instrument Info

User	IZKF
Instrument	FLEX-PC

D:\Data\IZKF2020\CB20_purified_Fr.10-16\0_C5\1 printed: 16.06.2020 12:49:49

Dr. Sven Rothemund, IZKF Leipzig, Liebigstrasse 21, 04103 Leipzig, Tel.: 0341-9715898, sven_r@yahoo.com

Comment 1 CB20_purified_Fr.10-16
Comment 2 MW:av.4343,1(M+H)+

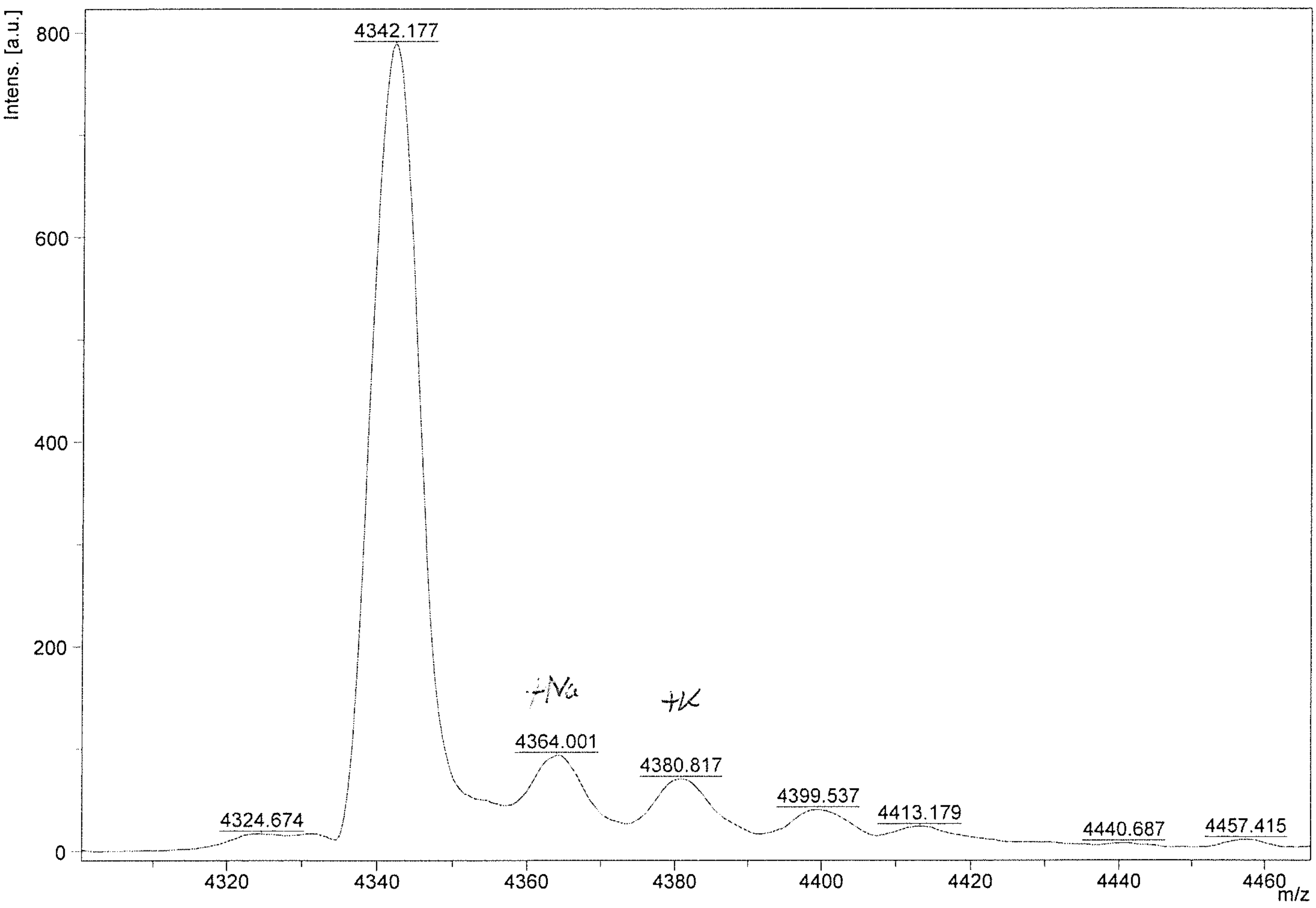

Acquisition Parameter

Date of acquisition	2020-06-16T12:49:15.719+02:00
Acquisition method name	D:\Methods\flexControlMethods\LP_PepMix.par
Aquisition operation mode	Linear
Voltage polarity	POS
Number of shots	86
Name of spectrum used for calibration	
Calibration reference list used	PeptideCalibStandardInsulin2 monoAv

Instrument Info

User	IZKF
Instrument	FLEX-PC

D:\Data\IZKF2020\CB20_purified_Fr.10-16\0_C5\1 printed: 16.06.2020 12:51:10

60 IZKF Leipzig, Core Unit Peptid-Technologien
Liebigstraße 21, 04103 Leipzig, e-mail: sven_r@yahoo.com, Tel.: 0341 - 9715898 / 897

Comment:

Sample Name:	**M21_purified**	*Injection Volume:*	**20.0**
Vial Number:	**RB1**	*Channel:*	**UV_VIS_1**
Sample Type:	**unknown**	*Wavelength:*	**220.0**
Control Program:	**Peptide_3D_basic_short**	*Bandwidth:*	**4**
Quantif. Method:	**peptide_izkf**	*Dilution Factor:*	**1.0000**
Recording Time:	**16/2/2021 12:33**	*Operator:*	**KEYUSER**
Sample ID:		*Sample Amount:*	**1.0000**

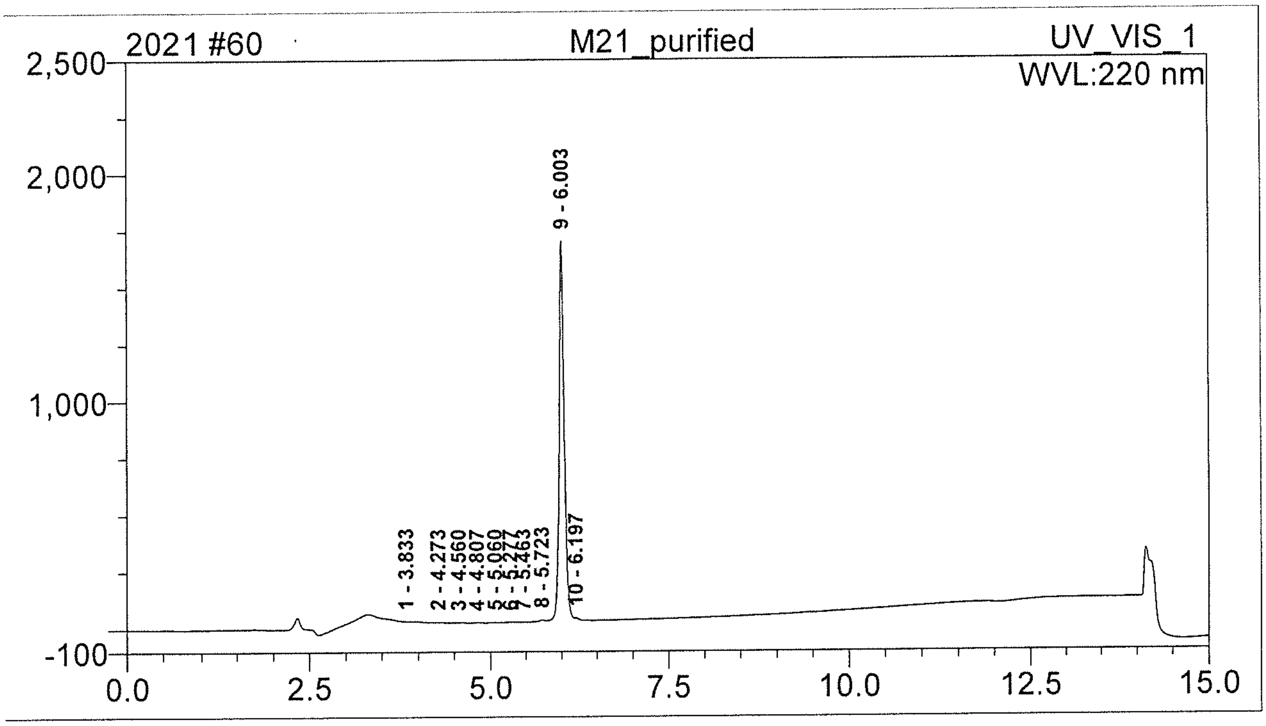

No.	Ret.Time min	Peak Name	Height mAU	Rel.Area %	Area mAU*min	Amount	Type
1	3.83	n.a.	0.0	0.19	0.27	n.a.	BMB
2	4.27	n.a.	1.4	0.13	0.19	n.a.	BMB
3	4.56	n.a.	1.0	0.09	0.13	n.a.	BMB
4	4.81	n.a.	1.4	0.11	0.15	n.a.	BMB
5	5.06	n.a.	0.9	0.07	0.10	n.a.	BMB
6	5.28	n.a.	0.9	0.07	0.10	n.a.	BMB
7	5.46	n.a.	0.7	0.04	0.06	n.a.	BMB
8	5.72	n.a.	7.2	0.48	0.69	n.a.	BM
9	6.00	n.a.	1670.1	97.94	139.18	n.a.	M
10	6.20	n.a.	13.8	0.88	1.25	n.a.	MB
Total:			1697.341	100.000	142.11	0.000	

Chromeleon (c) Dionex 1996-2006
Version 6.80 SR15 Build 4656 (243203)

Dr. Sven Rothemund, IZKF Leipzig, Liebigstrasse 21, 04103 Leipzig, Tel.: 0341-9715898, sven_r@yahoo.com

Comment 1	M21_purified
Comment 2	MW:4356,9(M+H)+

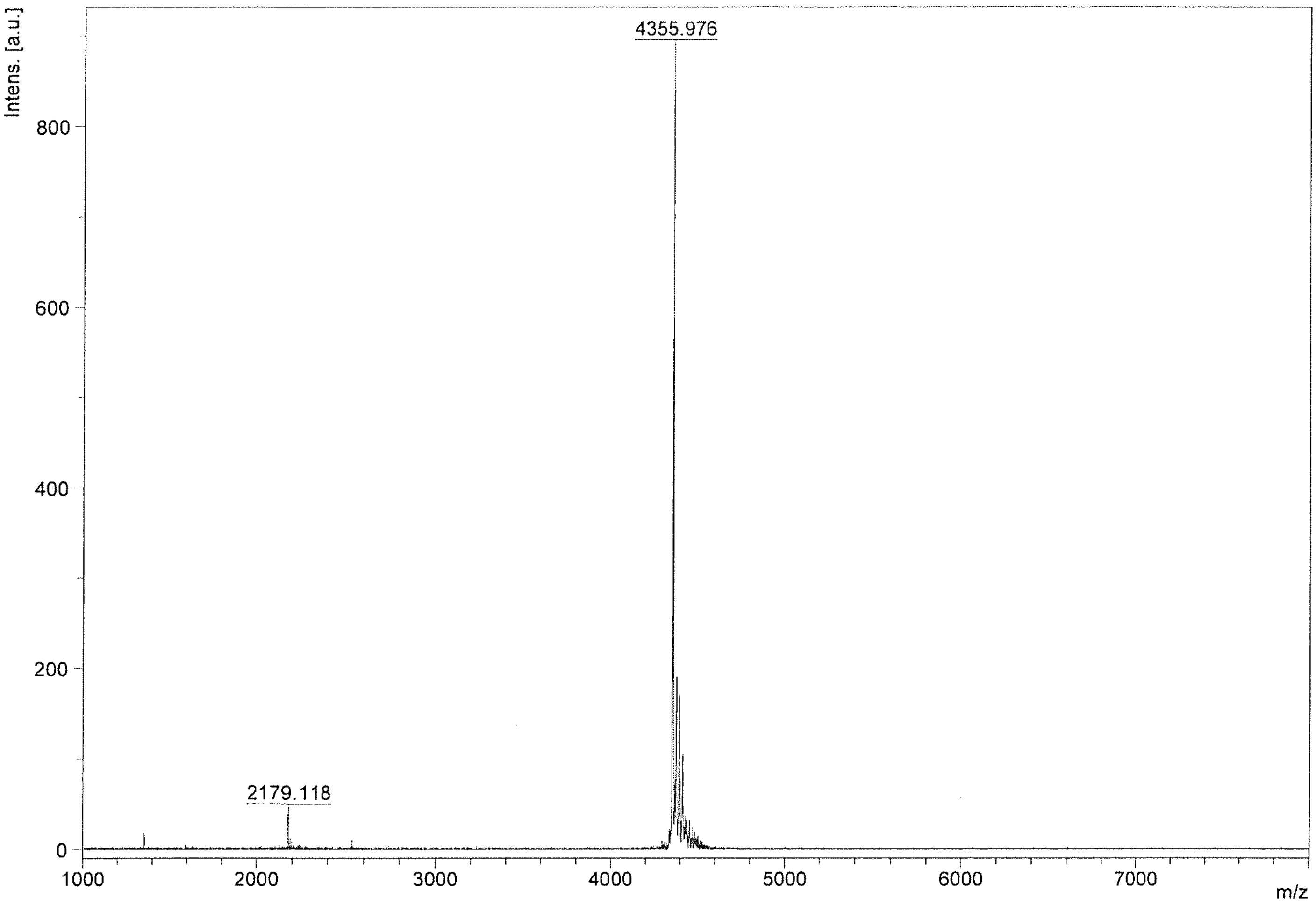

Acquisition Parameter

Date of acquisition	2021-02-16T13:02:03.489+01:00
Acquisition method name	D:\Methods\flexControlMethods\LP_PepMix.par
Aquisition operation mode	Linear
Voltage polarity	POS
Number of shots	73
Name of spectrum used for calibration	
Calibration reference list used	PeptideCalibStandardInsulin2 monoAv

Instrument Info

User	IZKF
Instrument	FLEX-PC

D:\Data\IZKF2021\M21_purified\0_C2\1 printed: 16.02.2021 13:02:48

Dr. Sven Rothemund, IZKF Leipzig, Liebigstrasse 21, 04103 Leipzig, Tel.: 0341-9715898, sven_r@yahoo.com

Comment 1 M21_purified
Comment 2 MW:4356,9(M+H)+

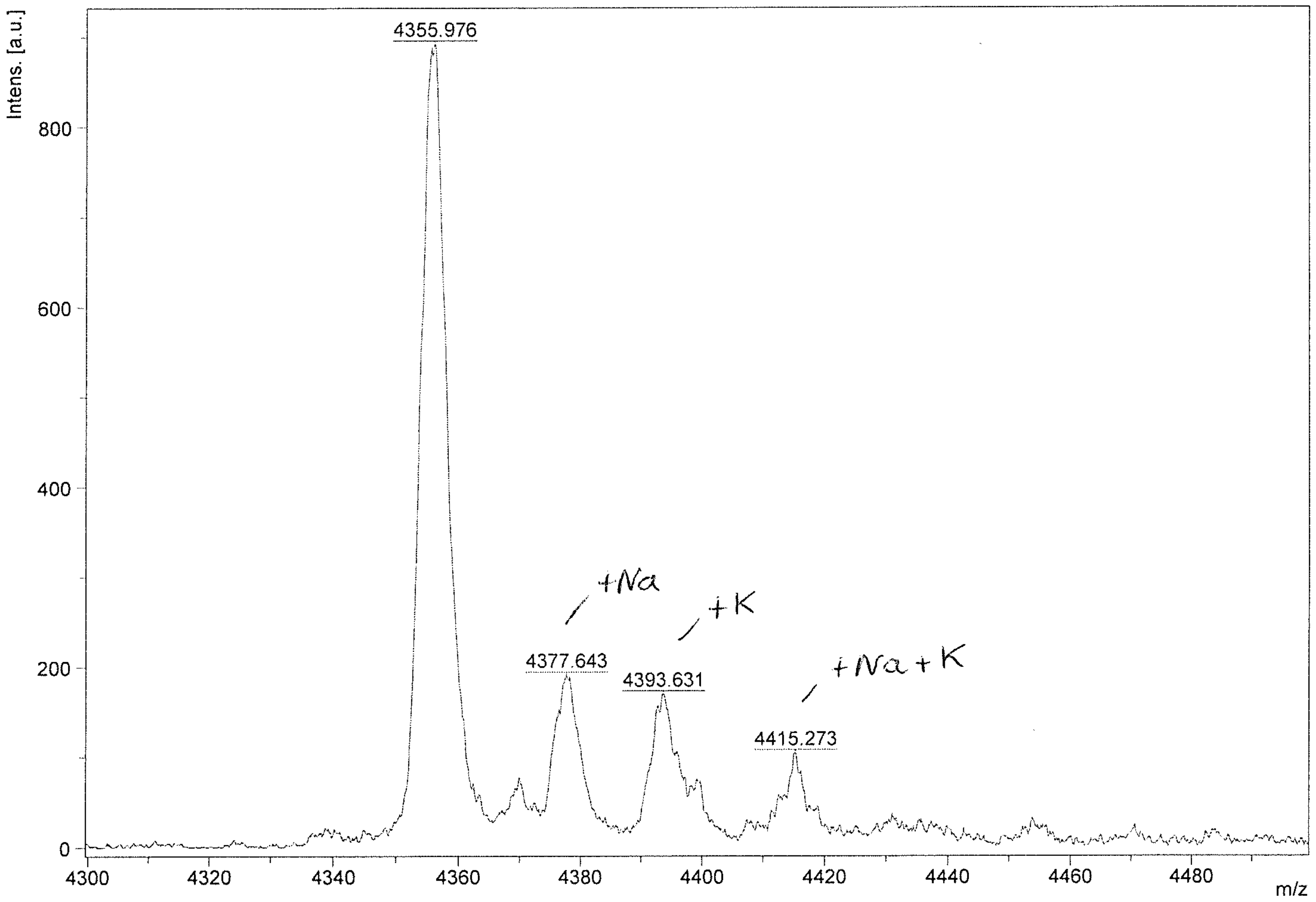

Acquisition Parameter

Date of acquisition	2021-02-16T13:02:03.489+01:00
Acquisition method name	D:\Methods\flexControlMethods\LP_PepMix.par
Aquisition operation mode	Linear
Voltage polarity	POS
Number of shots	73
Name of spectrum used for calibration	
Calibration reference list used	PeptideCalibStandardInsulin2 monoAv

Instrument Info

User	IZKF
Instrument	FLEX-PC

657 **IZKF Leipzig, Core Unit Peptid-Technologien**

Liebigstraße 21, 04103 Leipzig, e-mail: sven_r@yahoo.com, Tel.: 0341 - 9715898 / 897

Comment:

Sample Name:	**EC20_purified**	*Injection Volume:*	**20.0**
Vial Number:	**RB2**	*Channel:*	**UV_VIS_1**
Sample Type:	**unknown**	*Wavelength:*	**220.0**
Control Program:	**Peptide_3D_basic_short**	*Bandwidth:*	**4**
Quantif. Method:	**Peptide_3D_basic**	*Dilution Factor:*	**1.0000**
Recording Time:	**19/10/2020 12:16**	*Operator:*	**KEYUSER**
Sample ID:		*Sample Amount:*	**1.0000**

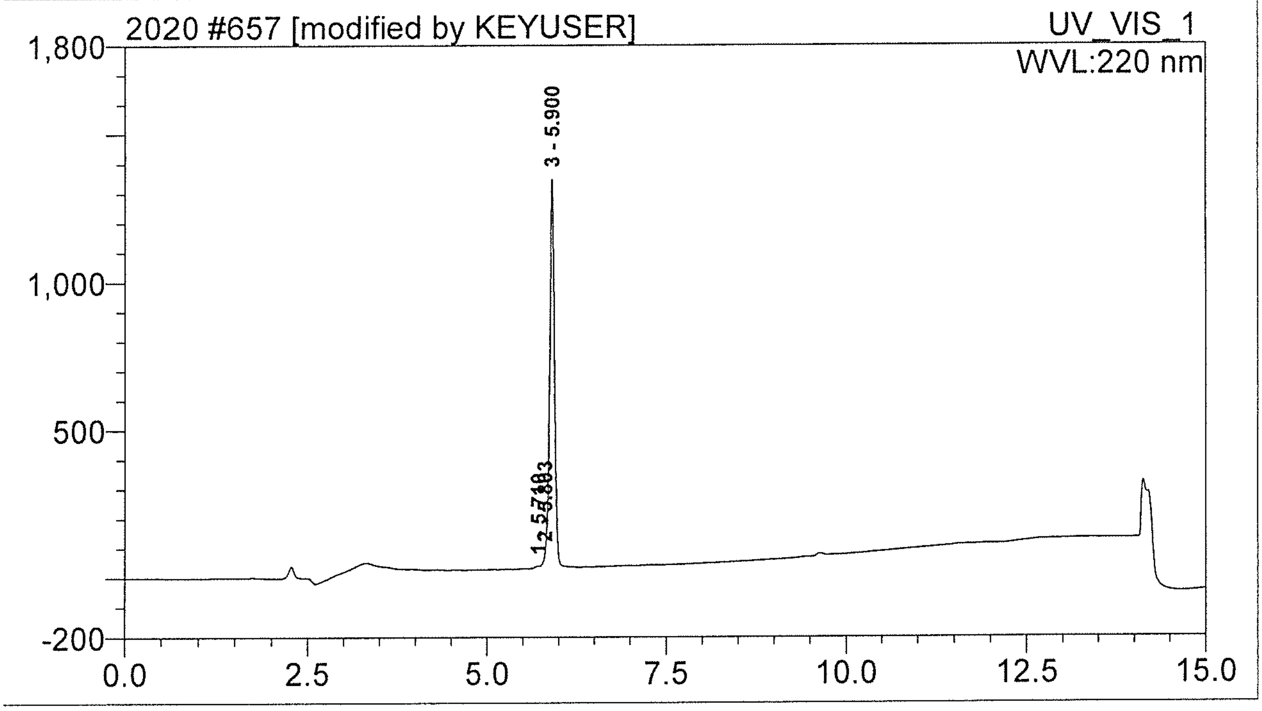

No.	Ret.Time min	Peak Name	Height mAU	Rel.Area %	Area mAU*min	Amount	Type
1	5.71	n.a.	6.8	0.38	0.39	n.a.	BM *
2	5.80	n.a.	48.1	1.43	1.50	n.a.	M *
3	5.90	n.a.	1313.5	98.19	102.41	n.a.	MB*
**Total:**			1368.496	100.000	104.30	0.000	

Dr. Sven Rothemund, IZKF Leipzig, Liebigstrasse 21, 04103 Leipzig, Tel.: 0341-9715898, sven_r@yahoo.com

Comment 1 EC20_purified

Comment 2 MW:4366,1(M+H)+

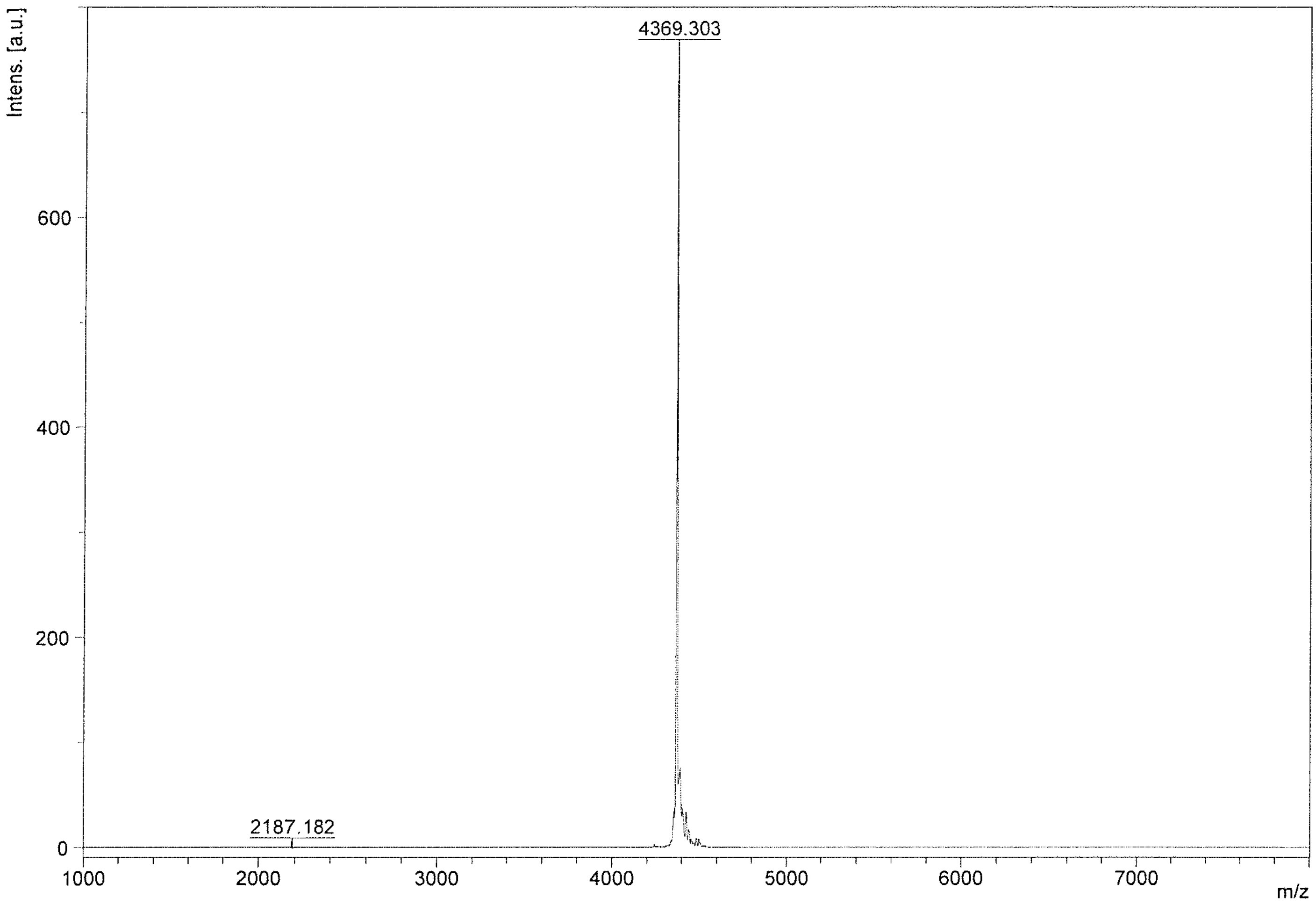

Acquisition Parameter

Date of acquisition	2020-10-19T12:50:50.188+02:00
Acquisition method name	D:\Methods\flexControlMethods\LP_PepMix.par
Aquisition operation mode	Linear
Voltage polarity	POS
Number of shots	21
Name of spectrum used for calibration	
Calibration reference list used	PeptideCalibStandardInsulin2 monoAv

Instrument Info

User	IZKF
Instrument	FLEX-PC

D:\Data\IZKF2020\EC20_purified\0_D11\1 printed: 19.10.2020 12:51:49

64 IZKF Leipzig, Core Unit Peptid-Technologien

Liebigstraße 21, 04103 Leipzig, e-mail: sven_r@yahoo.com, Tel.: 0341 - 9715898 / 897

Comment:

Sample Name:	**N21_purified**	*Injection Volume:*	**20.0**
Vial Number:	**RC3**	*Channel:*	**UV_VIS_1**
Sample Type:	**unknown**	*Wavelength:*	**220.0**
Control Program:	**Peptide_3D_basic_short**	*Bandwidth:*	**4**
Quantif. Method:	**peptide_izkf**	*Dilution Factor:*	**1.0000**
Recording Time:	**17/2/2021 12:38**	*Operator:*	**KEYUSER**
Sample ID:		*Sample Amount:*	**1.0000**

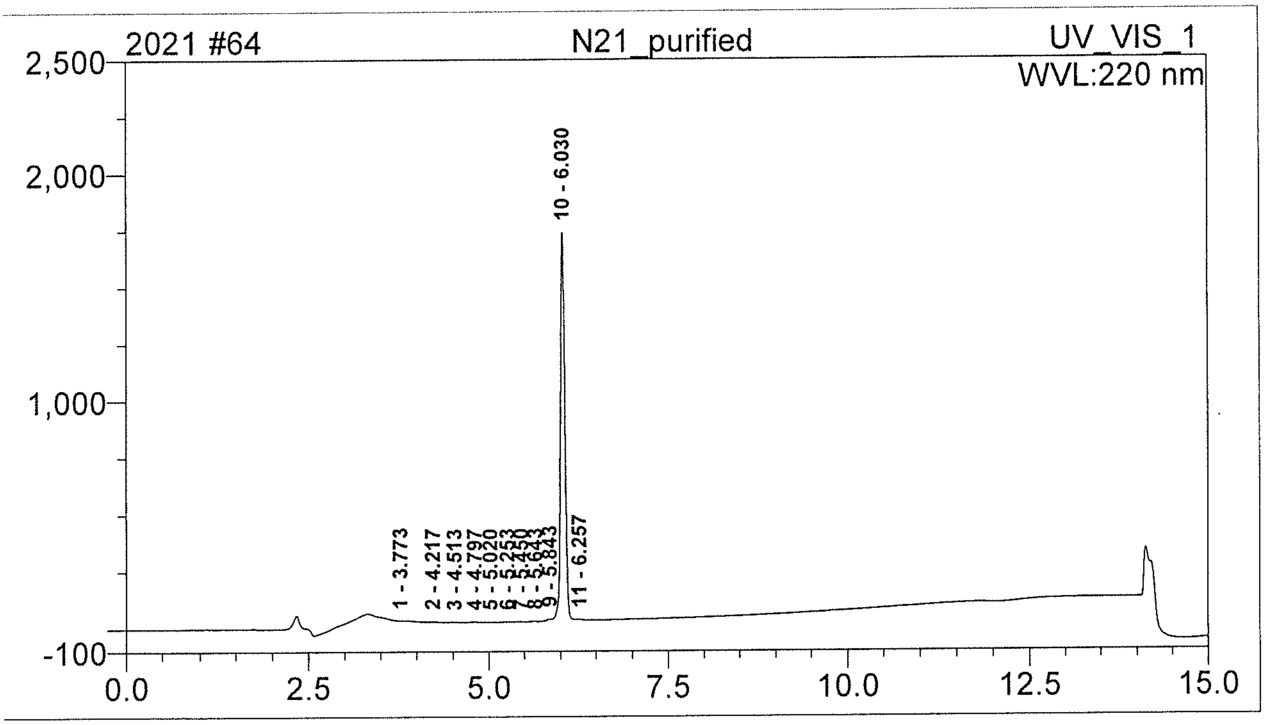

No.	Ret.Time min	Peak Name	Height mAU	Rel.Area %	Area mAU*min	Amount	Type
1	3.77	n.a.	0.0	0.20	0.26	n.a.	BMB
2	4.22	n.a.	1.4	0.16	0.20	n.a.	BMB
3	4.51	n.a.	1.1	0.11	0.14	n.a.	BMB
4	4.80	n.a.	2.6	0.20	0.25	n.a.	BM
5	5.02	n.a.	0.9	0.08	0.10	n.a.	MB
6	5.25	n.a.	1.0	0.08	0.10	n.a.	BMB
7	5.45	n.a.	1.2	0.08	0.11	n.a.	BMB
8	5.64	n.a.	0.9	0.08	0.10	n.a.	BMB
9	5.84	n.a.	8.8	0.51	0.64	n.a.	BM
10	6.03	n.a.	1707.9	98.08	123.46	n.a.	M
11	6.26	n.a.	5.6	0.42	0.53	n.a.	MB
**Total:**			1731.274	100.000	125.88	0.000	

Chromeleon (c) Dionex 1996-2006
Version 6.80 SR15 Build 4656 (243203)

Dr. Sven Rothemund, IZKF Leipzig, Liebigstrasse 21, 04103 Leipzig, Tel.: 0341-9715898, sven_r@yahoo.com

Comment 1	N21_purified
Comment 2	MW:4370,1(M+H)+

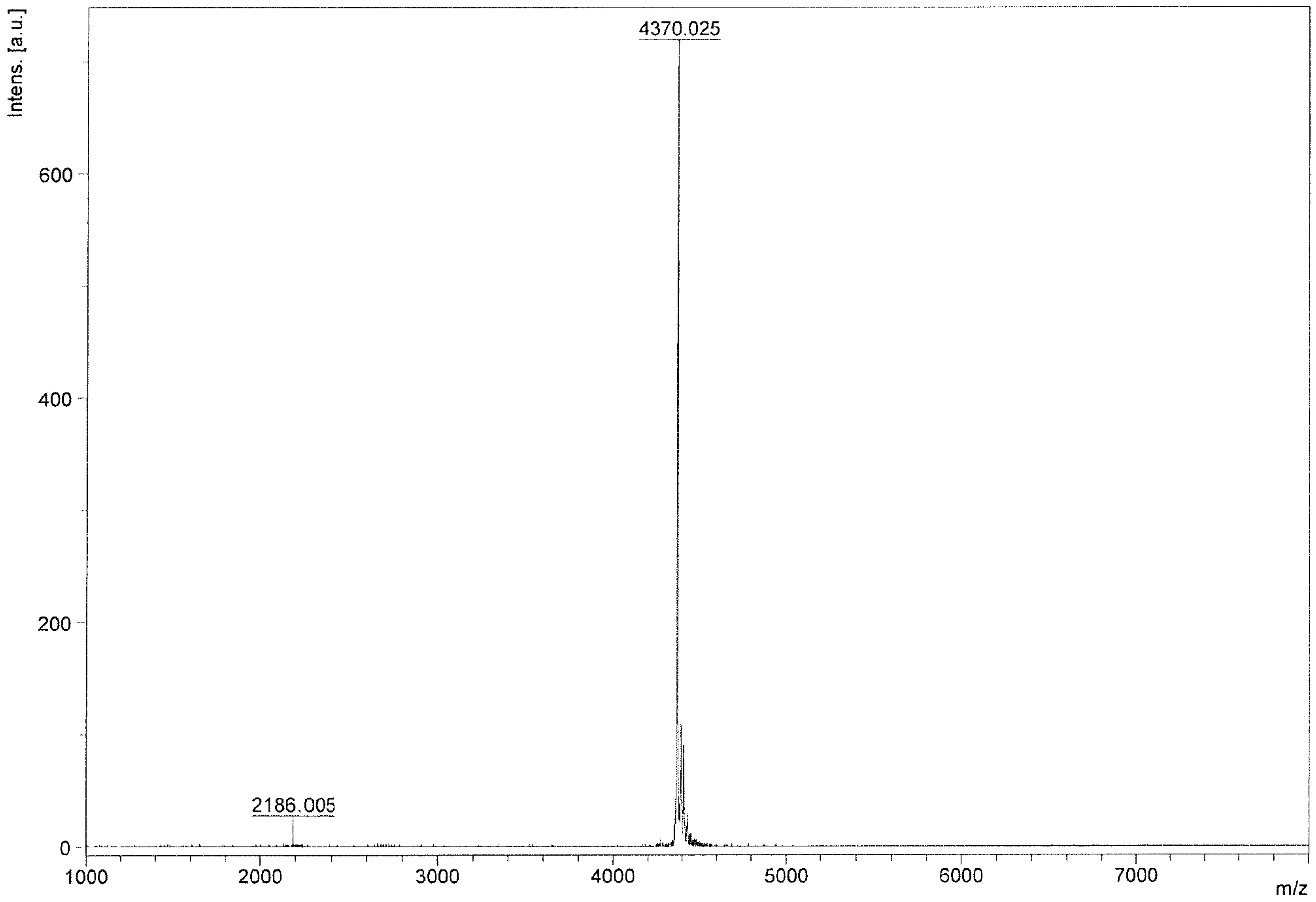

Acquisition Parameter

Date of acquisition	2021-02-17T13:00:50.428+01:00
Acquisition method name	D:\Methods\flexControlMethods\LP_PepMix.par
Aquisition operation mode	Linear
Voltage polarity	POS
Number of shots	44
Name of spectrum used for calibration	
Calibration reference list used	PeptideCalibStandardInsulin2 monoAv

Instrument Info

User	IZKF
Instrument	FLEX-PC

D:\Data\IZKF2021\N21_purified\0_D3\1 printed: 17.02.2021 13:01:53

Dr. Sven Rothemund, IZKF Leipzig, Liebigstrasse 21, 04103 Leipzig, Tel.: 0341-9715898, sven_r@yahoo.com

Comment 1	N21_purified
Comment 2	MW:4370,1(M+H)+

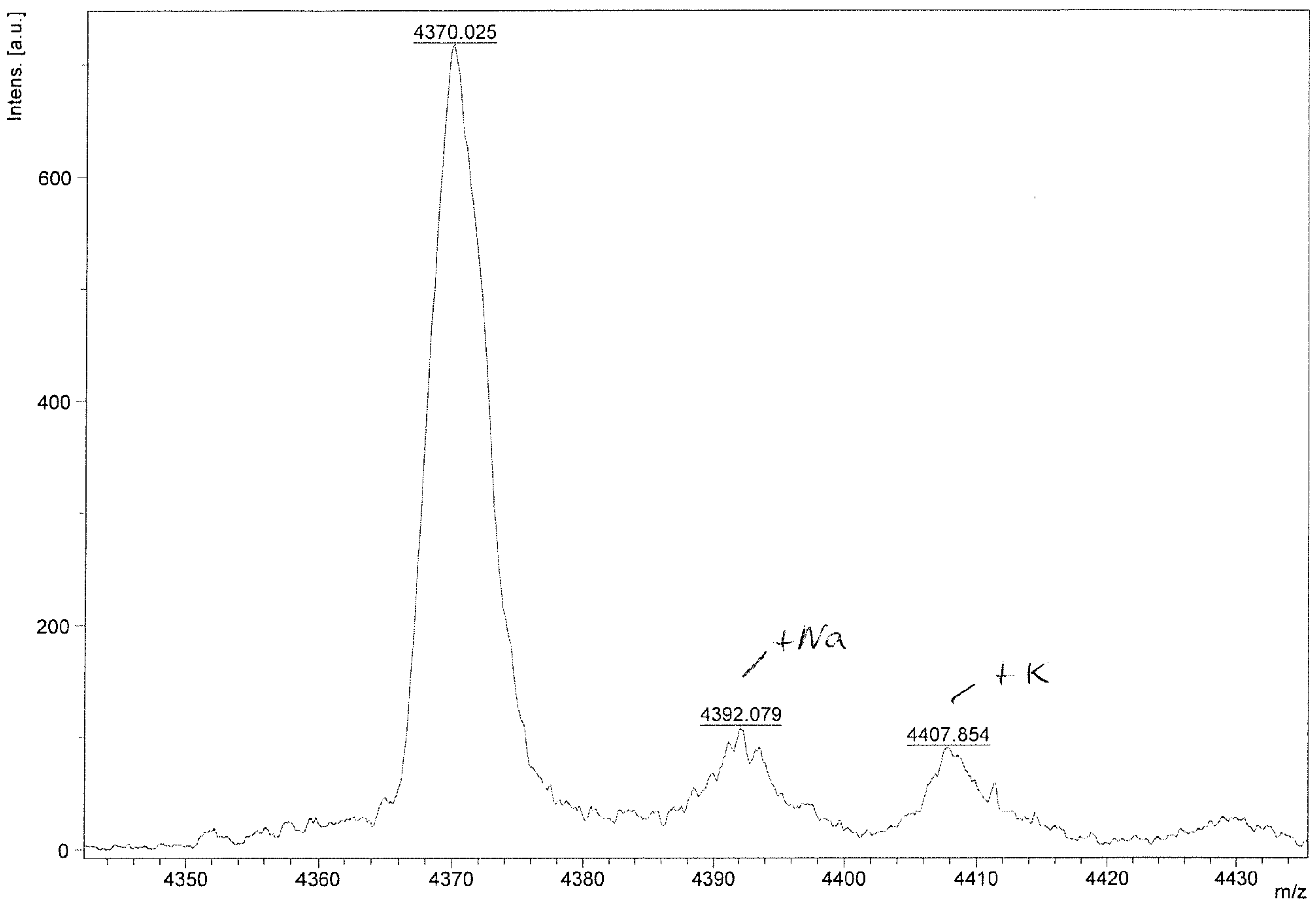

Acquisition Parameter

Date of acquisition	2021-02-17T13:00:50.428+01:00
Acquisition method name	D:\Methods\flexControlMethods\LP_PepMix.par
Aquisition operation mode	Linear
Voltage polarity	POS
Number of shots	44
Name of spectrum used for calibration	
Calibration reference list used	PeptideCalibStandardInsulin2 monoAv

Instrument Info

User	IZKF
Instrument	FLEX-PC

References

1. Korn, A., Surendran, D., Krueger, M., Maiti, S. & Huster, D. Ring structure modifications of phenylalanine 19 increase fibrillation kinetics and reduce toxicity of amyloid β (1-40). *Chem. Commun.* **54,** 5430–5433; 10.1039/c8cc01733f (2018).
